# Structural basis of transcription of the hachimoji eight-letter alphabet by *E. coli* RNA polymerase

**DOI:** 10.1038/s41467-026-76668-0

**Published:** 2026-09-02

**Authors:** Qingrong Li, Hyo-Joong Kim, Yan Liu, Juntaek Oh, Peini Hou, Shuichi Hoshika, Grigore Pintilie, Sriram Aiyer, Jenny Chong, Dmitry Lyumkis, Steven A. Benner, Dong Wang

**Affiliations:** 1 Department of Pharmaceutical Sciences, Skaggs School of Pharmacy and Pharmaceutical Sciences, University of California San Diego, La Jolla, CA USA; 2 Foundation for Applied Molecular Evolution, Alachua, FL USA; 3 Division of CryoEM and Bioimaging, Stanford Synchrotron Radiation Lightsource, SLAC National Accelerator Laboratory, Stanford University, Menlo Park, CA USA; 4 Department of Bioengineering, James H. Clark Center, Stanford University, Stanford, CA USA; 5 The Salk Institute for Biological Studies, La Jolla, CA USA; 6 Department of Integrative Structural and Computational Biology, The Scripps Research Institute, La Jolla, CA USA; 7 Graduate School of Biological Sciences, Section of Molecular Biology, University of California San Diego, La Jolla, CA USA; 8 Department of Molecular and Cellular Medicine, School of Medicine, University of California San Diego, La Jolla, CA USA; 9 Department of Biochemistry and Molecular Biophysics, University of California San Diego, La Jolla, CA USA; 10 Present Address: Department of Regulatory Science, Graduate School, Kyung Hee University, Seoul, Republic of Korea; 11 Present Address: Institute of Regulatory Innovation through Science, Kyung Hee University, Seoul, Republic of Korea; 12 Present Address: Institute of Integrated Pharmaceutical Sciences, Kyung Hee University, Seoul, Republic of Korea

**Keywords:** Cryoelectron microscopy, Synthetic biology, Bacterial transcription

## Abstract

Expanded genetic alphabets with synthetic nucleotides can greatly increase the chemical diversity of nucleic acids, enabling new molecular functions. Because cellular transcription is executed by multi-subunit RNA polymerases, the compatibility of unnatural base pairs with this machinery is essential for engineering expanded genetic systems. Here we demonstrate that *Escherichia coli* RNA polymerase efficiently transcribes an eight-letter genetic alphabet with two orthogonal unnatural base pairs: P:Z and B:S pairs. To overcome G:Z misincorporation, we synthesize a higher-fidelity analogue, termed Z*, in which the C5 nitro group is replaced with a carboxamide. To elucidate substrate-recognition mechanisms, we determine four cryo-electron microscopy structures of RNA polymerase incorporating dZ:PTP or dP:Z*TP at 2.42–2.75 Å resolution. These structures, together with our early work on S:B pair, show that *E. coli* RNA polymerase is able to efficiently recognize these unnatural base pairs in the same manner as natural base pairs. Collectively, these results establish the feasibility of an eight-letter genetic alphabet for transcription.

## Abstract (web summary)

Expanded genetic alphabets can increase the functional diversity of nucleic acids, but their compatibility with cellular transcription is uncertain. Here, the authors show that bacterial RNA polymerase faithfully transcribes an eight‑letter genetic alphabet and recognises unnatural base pairs through mechanisms similar to those used for natural DNA.

## Introduction

All known life on Earth relies on a four-letter genetic alphabet—adenine (A), thymine or uracil (T or U), cytosine (C), and guanine (G)—to store and express genetic information. A long-standing goal in synthetic biology has been to create orthogonal nucleotides capable of forming unnatural base pairs (UBPs) that expand this genetic alphabet^1,2^. Such expansion would greatly increase the chemical diversity of nucleic acids, enabling new molecular functions and properties.

**Fig. 1:**
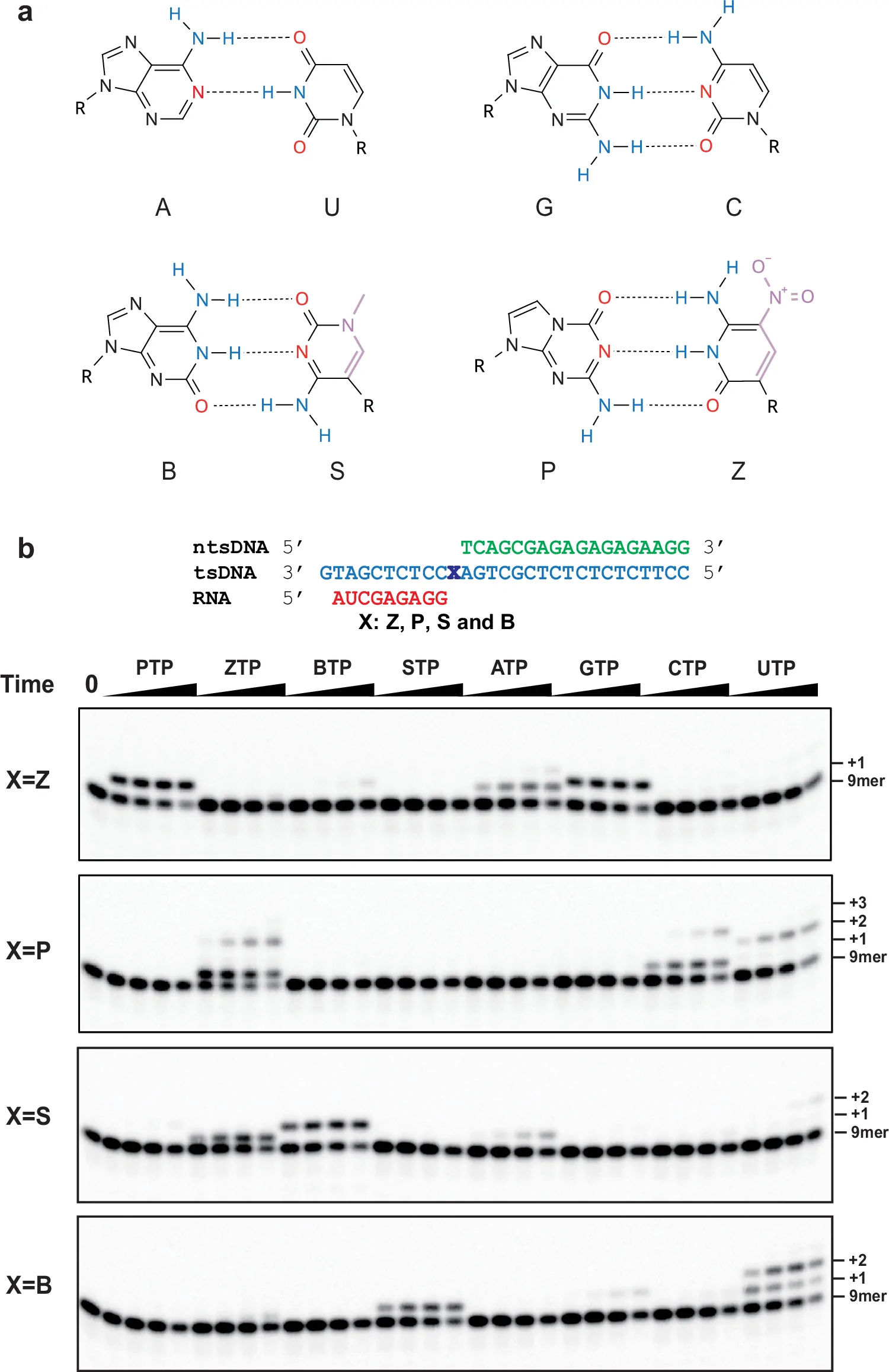
Single-nucleotide transcription assay demonstrating recognition of the P:Z base pair by *E. coli* RNA polymerase. **a** Chemical structures of the Hachimoji eight-letter base pairs used in this study. Hydrogen-bond donors and acceptors are colored in blue and red, respectively. Regions highlighted in pink denote the functional groups that distinguish these unnatural nucleotides from natural bases. **b** Transcription assay testing all eight Hachimoji nucleotide triphosphates against four nucleic acid scaffolds containing either dZ, dP, dS or dB at the template i + 1 position. The upper panel shows the nucleic acid scaffold composed of template-strand DNA (tsDNA, cyan), non-template-strand DNA (ntsDNA, green), and RNA (red). “X” marks the templating position (dZ, dP, dS, or dB) for nucleotide incorporation. The lower panel shows denaturing PAGE analysis of transcription products. Each reaction contained 20 μM of the indicated nucleotide triphosphate and was sampled at 0, 15 s, 1 min, 5 min, and 30 min. The 0-s band corresponds to the p^32^-labeled 9-mer RNA (5′-AUGGAGAGG-3′), which serves as an internal molecular-weight marker. Bands labeled +1, +2, and +3 represent transcription products extended by one, two, and three nucleotides (10-, 11-, and 12-nt products), respectively. All assays were performed in two independent replicates.

Multiple laboratories have developed UBPs based on different strategies, including altered hydrogen-bonding patterns, the absence of hydrogen bonds, or purely shape-complementary designs^3–5^. Among these efforts, the Artificially Expanded Genetic Information System (AEGIS) introduced UBPs that rearrange hydrogen-bonding groups while preserving overall Watson–Crick geometry, generating an eight-letter genetic alphabet (Hachimoji) composed of four natural and four unnatural nucleotides^6^ (Fig. 1a).

To integrate AEGIS UBPs into cellular biosynthesis, it is essential to elucidate how they are recognized during the central dogma processes. AEGIS nucleotides are well accepted by Taq DNA polymerase during DNA replication and by the single-subunit bacteriophage T7 RNA polymerase during transcription^7,8^. For transcription by cellular multi-subunit RNA polymerases, the RNA polymerase elongation complex containing the AEGIS base pair B:S has been structurally characterized, revealing that B:S pair adopts a Watson–Crick–like geometry in the active site^9^. The B:S pair has also been used to expand the amino acid lexicon in in vitro protein translation^10^.

In contrast, the transcriptional mechanism of the AEGIS base pair P:Z by multi-subunit RNA polymerases has remained uncharacterized. Here, we investigate the recognition and incorporation of the P:Z base pair by *E. coli* RNA polymerase (RNAP) using a combined biochemistry and structural biology approach. We showed that the eight-letter genetic system functions robustly in transcription by *E. coli* RNAP, and the two UBPs, P:Z and B:S, are orthogonal. Because Z carrying a nitro group can undergo deprotonation and then mispair with G, we synthesized oligonucleotides containing a ribonucleoside analogue (Z*) that replaced the C5 nitro group with a carboxamide group and introduced a 2′-fluoro substituent to manage tautomerism^9^. By increasing the pK_a_ of the nucleobase, this analog markedly reduced G:Z* mispairing during transcription.

To further elucidate the structural basis of P:Z and P:Z* transcription recognition, we reconstituted *E. coli* RNAP elongation complexes containing dZ:PTP or dP:Z*TP at the i + 1 position and determined four cryo-EM structures at resolutions of 2.42–2.75 Å. In all structures, both P:Z and P:Z* adopt canonical Watson–Crick geometries in the active site and induce trigger loop (TL) folding during substrate loading, closely paralleling the behavior of natural base pairs. These structural observations demonstrate that multi-subunit RNAP can faithfully accommodate P:Z and P:Z*, establishing a mechanistic foundation for the transcription of an expanded genetic alphabet. Together with our earlier work on S:B, our findings show that P:Z and S:B operate as a native-like base pair in *E. coli* RNAP and supports the feasibility of stably expanding the genetic alphabet to eight letters.

## Results

### Base pairing of 8-letter genetic alphabet in *E. coli* RNAP transcription

The P:Z and B:S base pairs expand the canonical four-letter genetic alphabet by introducing alternative hydrogen-bonding patterns that remain compatible with Watson-Crick geometry in DNA duplex (Fig. 1a). Prior work has shown that B:S pair can serve as 5^th^ and 6^th^ bases to support efficient *E. coli* RNAP transcription^9^. Here, we sought to test whether we can expand to eight-letter genetic alphabet and determine how *E. coli* RNAP selects and incorporates nucleotides opposite the P:Z pair.

We performed single-nucleotide transcription assays using DNA/RNA hybrid scaffolds containing a site-specific unnatural base at the +1 position of the template strand (dP, dZ, dB, dS) (Fig. 1b). Eight nucleotide triphosphates—including all four natural NTPs and four unnatural triphosphates—were tested for incorporation opposite the templating base by *E. coli* RNAP. In all scaffolds tested, we found that addition of the cognate partner nucleotide effectively produced a clear band shift from the 9-mer RNA to the n + 1 product within 15 s incubation, demonstrating that both P:Z and S:B are efficiently and selectively incorporated as orthogonal unnatural base pairs during transcription (Fig. 1b; Supplementary Fig. 1). In addition, single-turnover kinetics measurements further showed that k_obs_ values for dZ:PTP and dP:ZTP are only approximately two-fold lower than those of the natural dG:CTP pair in the same conditions, demonstrating that P:Z incorporation is comparable to natural nucleotide incorporation for natural base pairs (Supplementary Fig. 2). Thus, the combined use of P:Z and B:S supports the feasibility of an expanded eight-letter genetic alphabet in which all four unnatural nucleotides pair selectively and independently, preserving informational integrity in transcription processes.

To comprehensively evaluate substrate recognition selectivity, we quantified band intensities across all 32 template-NTP combinations (four scaffolds × eight NTPs (ATP, GTP, CTP, UTP, PTP, ZTP, BTP, and STP)) at the 15-s time point (Supplementary Fig. 1). For all the template-NTP combinations tested, the cognate unnatural substrate incorporations are among the most efficient ones as expected. Substrate discrimination between cognate and mismatch substrates is most pronounced at the early time point, indicating misincorporation is very slow in comparison with cognate substrate incorporation for most cases.

dB:UTP and dZ:GTP misincorporation stand out as two major misincorporations (Fig. 1b, Supplementary Fig. 1). We have previously kinetically characterized dB:UTP misincorporation and revealed the structural basis of dB:UTP transcription recognition. These studies showed that the isoguanine heterocycle in the dB:UTP mismatch adopts rare tautomer to form Watson–Crick like base pair at the active site of *E. coli* RNAP^9^.

**Fig. 2:**
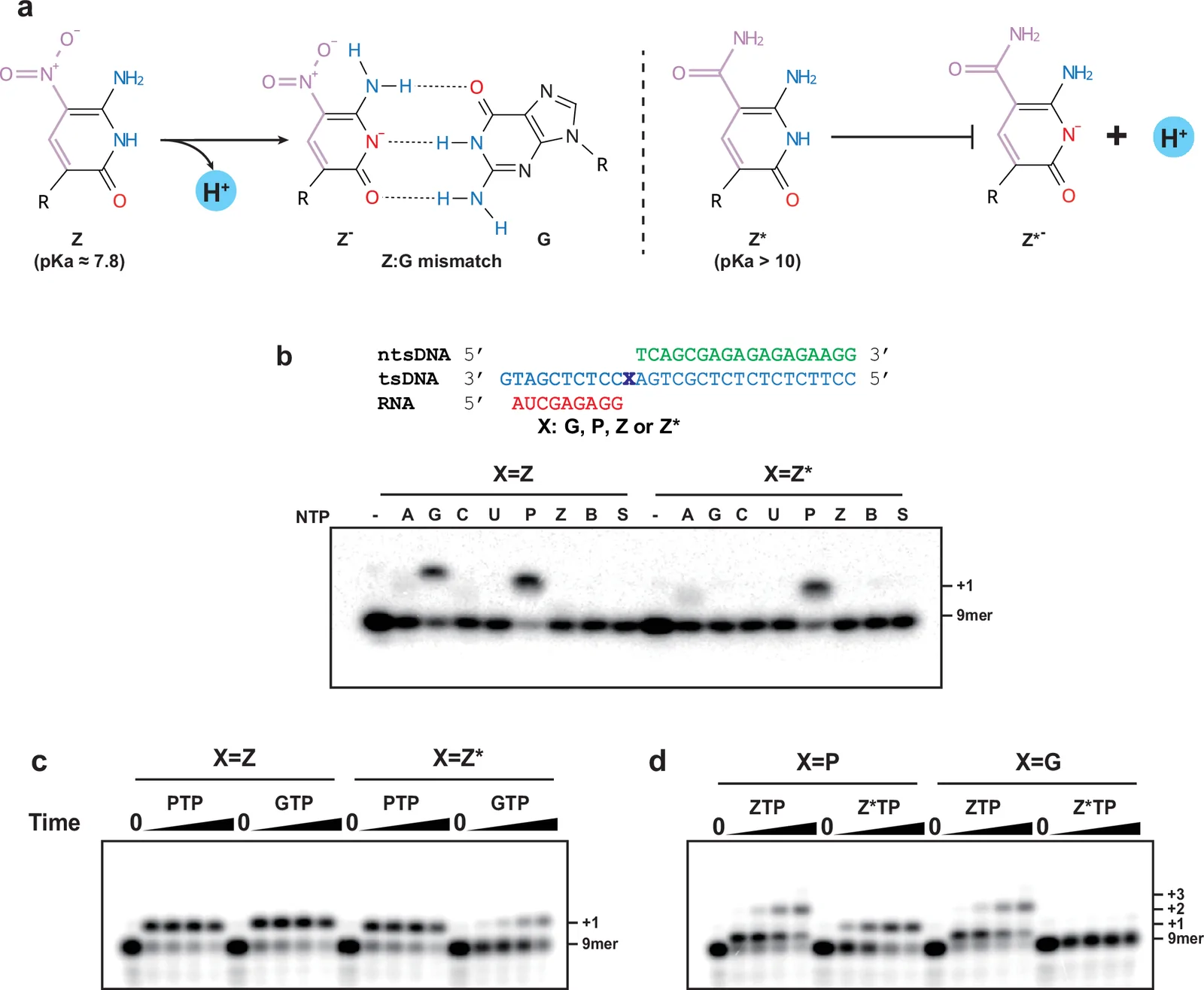
Z* suppresses Z:G mismatch incorporation by *E. coli* RNA polymerase. **a** Chemical mechanism underlying Z deprotonation. The left panel illustrates how Z undergoes deprotonation to form Z^-^, which can mispair with guanine to generate a Z^-^:G base pair. The right panel shows the structure of 2′-F-α-carboxamide-Z (Z*), in which substitution of the nitro group with a carboxamide raises the pK_a_ above 10, thereby preventing deprotonation and reducing G mispairing. Hydrogen-bond donors and acceptors are colored blue and red, respectively. Regions highlighted in pink denote functional groups that distinguish these unnatural nucleotides from natural bases. **b** Single-nucleotide incorporation assays for comparing scaffolds containing dZ or dZ* at the template i + 1 position. The nucleic acid scaffold used in **b**–**d**—comprising template-strand DNA (tsDNA, cyan), non-template-strand DNA (ntsDNA, green), and RNA (red)—is shown in the upper panel of (**b**). “X” marks the template i + 1 position. For the assay in (**b**), *E. coli* RNAP elongation complexes were assembled on each scaffold and treated with 1 μM of the indicated NTP substrate, including all four natural NTPs (ATP, GTP, CTP, and UTP) and all four unnatural NTPs (PTP, ZTP, BTP, and STP). Reactions were sampled at 10 s, resolved by 12% denaturing urea-PAGE, and visualized by autoradiography of p^32^-labeled RNA. These assays were performed in three independent replicates. **c**, **d** Single-nucleotide incorporation assays comparing the mismatch tendencies of Z and Z* opposite guanine. The left **c** shows PTP and GTP incorporation opposite dZ or dZ*. The right **d** shows ZTP and Z*TP incorporation opposite dP or dG. Each reaction contained 20 μM of the indicated nucleotide triphosphate and was sampled at 0, 30 s, 1 min, 10 min, and 30 min. The “9-mer” band corresponds to the p^32^-labeled RNA primer, while “+1” through “+3” indicate extended transcription products (10-nt to 12-nt products). These assays were performed in two independent replicates.

For the P:Z system, dZ:GTP misincorporation is substantially higher than that of any other non-cognate pair. Similar Z:G mismatch behaviors have previously been observed during DNA replication and PCR. Benner and coworkers reported that deprotonated Z (Z⁻) can pair with G through a complementary hydrogen-bonding pattern, forming a Watson–Crick-like geometry (Fig. 2a)^7,11^. Consistent with this, the fidelity of Z:P has been shown to be pH-dependent in PCR reactions with Taq polymerase, supporting the involvement of Z deprotonation rather than polymerase-specific interactions^7^.

By analogy, the Z:G mismatch observed in our transcription assay most likely arises from the intrinsic hydrogen-bonding properties of the Z:P system—specifically, the ability of Z to deprotonate and adopt a complementary pattern that pairs with G—rather than from any unusual or idiosyncratic interaction between P:Z base pair and *E. coli* RNAP. In addition, we also noted other minor mismatches, including dP:UTP, dP:CTP, dZ:ATP, dS:ZTP, and dS:ATP. However, cognate incorporation proceeds with substantially higher efficiency than misincorporation under the same conditions, enabling kinetic discrimination. Note that as an intrinsic limitation in these single-nucleotide transcription assays, we “force” the misincorporation of single substrate in the absence of competition by the cognate complementary nucleoside triphosphate. Therefore, the misincorporation frequencies observed here are likely overestimates of the actual error rates when the cognate substrate (and all of the other NTPs) are present and compete simultaneously. The correctly complementary cognate substrates would kinetically outcompete these rare and slow mismatches in such complete systems.

### A higher selective Z base analog: Z* for transcription

To reduce the intrinsic mismatch propensity of Z, the central strategy is to suppress formation of its deprotonated form, which is Watson-Crick complementary to G. The nitro group of Z, positioned in the major groove, is a strong electron-withdrawing substituent that lowers the pK_a_ of the nucleobase to approximately 7.8, thereby favoring deprotonation under physiological conditions. To mitigate this effect, we employed the recently developed 2′-F-α-carboxamide-Z (Z*)^12^, which replaced the nitro group with a carboxamide moiety (the complete chemical structures of P, Z, and Z* in their NTP substrate and template DNA forms are shown in Supplementary Fig. 3). The pK_a_ of Z* exceeds 10 and thus greatly disfavors deprotonation at neutral pH (Fig. 2a).

To systematically assess how the Z* modification affects the full substrate selectivity profile of *E. coli* RNAP, we tested all eight Hachimoji NTPs against dZ and dZ* template scaffolds at 1 μM substrate concentration and a 10-s reaction time (Fig. 2b; Supplementary Fig. 4). Cognate incorporation of PTP was clearly detected opposite both dZ and dZ*, confirming that Z* retains functional P:Z pairing capacity. In contrast, misincorporation of GTP detectable with the dZ scaffold was substantially reduced with dZ*, indicating that the carboxamide substitution substantially improves substrate selectivity. No other major misincorporation were observed for the dZ* template. A more complete characterization using higher substrate concentrations (20 μM) and extended reaction times (up to 30 min), including band intensity quantification, is provided in Fig. 2c, further confirming the improved selectivity for the dZ* template over the dZ template.

We next assessed the selectivity of incoming ZTP and Z*TP opposite dP in the template (Fig. 2d). Both nucleotides supported efficient cognate incorporation opposite P (*n* + 1 band). However, we also observed higher bands indicating further extension up to the *n* + 3 position due to ZTP misincorporation against dA and dG. In contrast, we observed the *n* + 1 product only for Z*TP incorporation opposite dP template. Similarly, we also observed multiple bands of ZTP misincorporation for the dG template (*n* + 1 and *n* + 3 bands). In contrast, no misincorporation bands were observed with Z*TP, indicating substantial improvement of selectivity. Notably, we also observed a substantial reduction in Z*TP misincorporation on the dS scaffold compared with ZTP (Supplementary Fig. 4c).

To test whether *E. coli* RNAP can continue elongation beyond the PZ site, we performed NTP-chase assays (Supplementary Fig. 5). Among all of the tested P:Z and P:Z* configurations, we found that *E. coli* RNAP can extend from *n* + 1 position and generate long RNA transcript efficiently. We observed no pausing at *n* + 2 site, indicating that *E. coli* RNAP can efficiently translocate from UBP site and add next nucleotide.

Together, these results demonstrate that the carboxamide substituent (Z*) significantly enhances the transcriptional fidelity of the P:Z pair and reduces dZ:GTP and dS:ZTP misincorporation. Importantly, neither P:Z nor P:Z* incorporation impairs subsequent processive elongation by *E. coli* RNAP.

### Cryo-EM structure of *E. coli* elongation complex with dZ:PTP

**Fig. 3:**
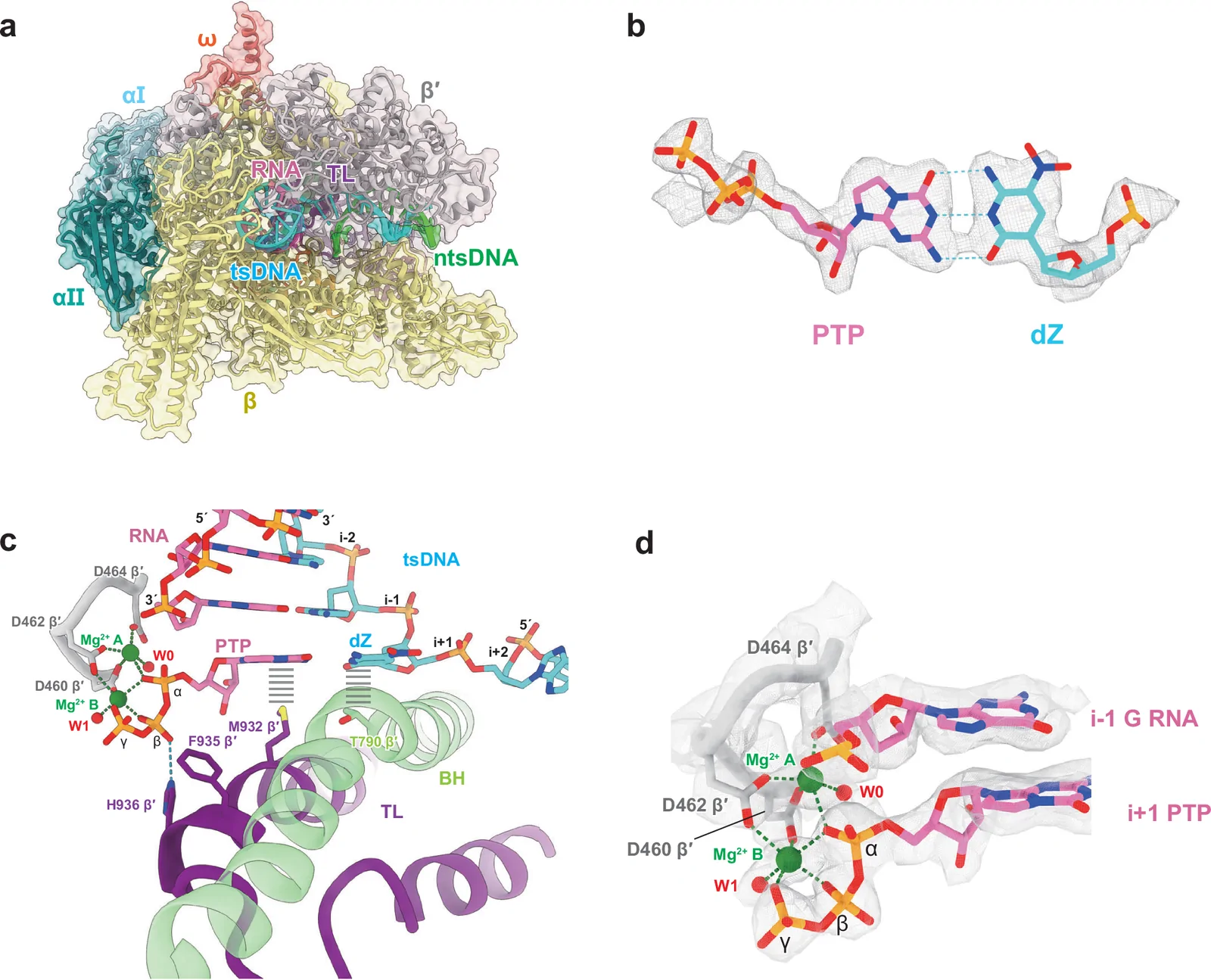
Cryo-EM structure of the *E. coli* RNA polymerase elongation complex containing dZ:PTP. **a** Overview of the cryo-EM structure of the *E. coli* RNAP elongation complex (EC) assembled with a dZ template and the incoming PTP substrate. The EC contains five RNAP subunits—αI (light blue), αII (teal), β (yellow), β′ (silver), and ω (salmon)—together with the nucleic-acid scaffold composed of tsDNA (cyan), ntsDNA (green), and RNA (hot pink). The trigger loop (TL) is highlighted in purple. **b** The incoming PTP (hot pink) forms a canonical Watson–Crick base pair with templating dZ (cyan). The cryo-EM density (contoured at 8-σ) is shown together with the atomic model. Hydrogen bonds are indicated by blue dashed lines. **c** Architecture of the active site in the dZ:PTP EC. Key structural elements include RNA, tsDNA, the P-loop (silver), TL (purple), two catalytic magnesium ions (green), and the bridge helix (BH; light green). Two water molecules coordinating the metals—W0 for metal A and W1 for metal B—are depicted as red spheres. Critical residues involved in substrate stabilization are shown as sticks: TL residues M932, F935, and H936, and BH residue T790. The hydrogen bond between H936 and the β-phosphate of PTP is shown as a blue dashed line. Hydrophobic interactions between TL/BH residues and the nucleobase moieties are depicted as black bold dashed lines. **d** Detailed view of the two-metal coordination. Both atomic model and cryo-EM density (8-σ) are shown. The two Mg²^+^ ions (green) coordinate the three catalytic aspartates of the P-loop (silver), the triphosphate of PTP (hot pink), and two ordered water molecules (red), forming a complete two-metal-ion geometry characteristic of a pre-catalytic RNAP active site.

To elucidate how a multi-subunit RNA polymerase recognizes and incorporates the P:Z base pair, we determined the cryo-EM structure of an *E. coli* RNAP elongation complex (EC) containing a dZ template paired with the incoming PTP nucleotide at the i + 1 position (Fig. 3; Supplementary Fig. 6; Supplementary Table 1). To trap a pre-catalytic state, we reconstituted the EC with a 3′-dOH RNA, which allows substrate binding but prevents phosphodiester bond formation, thereby stabilizing pre-incorporation intermediates. The cryo-EM reconstruction reached 2.42 Å resolution, with all five RNAP subunits—αI, αII, β, β′, and ω—clearly resolved (Fig. 3A).

The resulting structure captures a well-defined PTP substrate positioned in the active site together with a fully folded trigger loop (TL; residues 916–1146 of β′), a hallmark of the catalytically competent, pre-catalytic conformation (Supplementary Fig. 6). The substrate PTP forms a canonical Watson–Crick base pair with the templating dZ (Fig. 3b), and its triphosphate adopts a characteristic chair-like conformation precisely coordinated by two catalytic magnesium ions (Fig. 3c, d).

The folded trigger loop (TL) was captured to sense the substrate PTP loading. In specific, TL residue M932 and bridge helix (BH) residue T790 jointly stabilize the nucleobases of PTP and dZ through hydrophobic interactions, while TL residue H936 forms a hydrogen bond with the β-phosphate of PTP (Fig. 3c). Comparison with published structures of RNAP ECs containing natural base pairs—including *E. coli* RNAP (PDB: 6RH3)—reveals that TL conformation and substrate positioning in our dZ:PTP complex closely resemble those in the native systems. Key TL residues adopt similar orientations across all structures, with only minor rotamer differences (Supplementary Fig. 7). These observations demonstrate that the P:Z base pair behaves analogously to a natural Watson-Crick pair during substrate loading and TL closure.

The high-resolution map further reveals a fully formed two-metal-ion catalytic configuration. Metal B (Mg²^+^) exhibits clear octahedral geometry, coordinating the α-, β-, and γ-phosphate groups of PTP in a tridentate arrangement, with a water molecule (W1) completing the coordination sphere (Fig. 3d). Asp460 and Asp462 (β′) bridge metals A and B, while metal A is further coordinated by the substrate α-phosphate, Asp464 (β′), an ordered water molecule (W0), and presumably the RNA 3′-OH (Fig. 3d). In previously reported RNA polymerase structures, the geometry of the catalytic metal coordination is often less well defined, largely because of limited map quality, and in some cases the density for metal B is not observed. To better assess the accuracy of our assignment, we therefore compared our active-site configuration to high-resolution structures of native RNA^13^ and DNA polymerases^14,15^. The metal coordination geometry and the chair-like triphosphate conformation in our EC are nearly identical to those observed in native polymerases (Supplementary Fig. 8).

Together, these results demonstrate that the P:Z base pair is accommodated by *E. coli* RNAP in a manner highly similar to natural substrates, supporting accurate Watson–Crick pairing, proper alignment of catalytic metals, and a fully folded TL conformation characteristic of a bona fide pre-catalytic state.

### Cryo-EM structure of *E. coli* elongation complex with dP:Z*TP

**Fig. 4:**
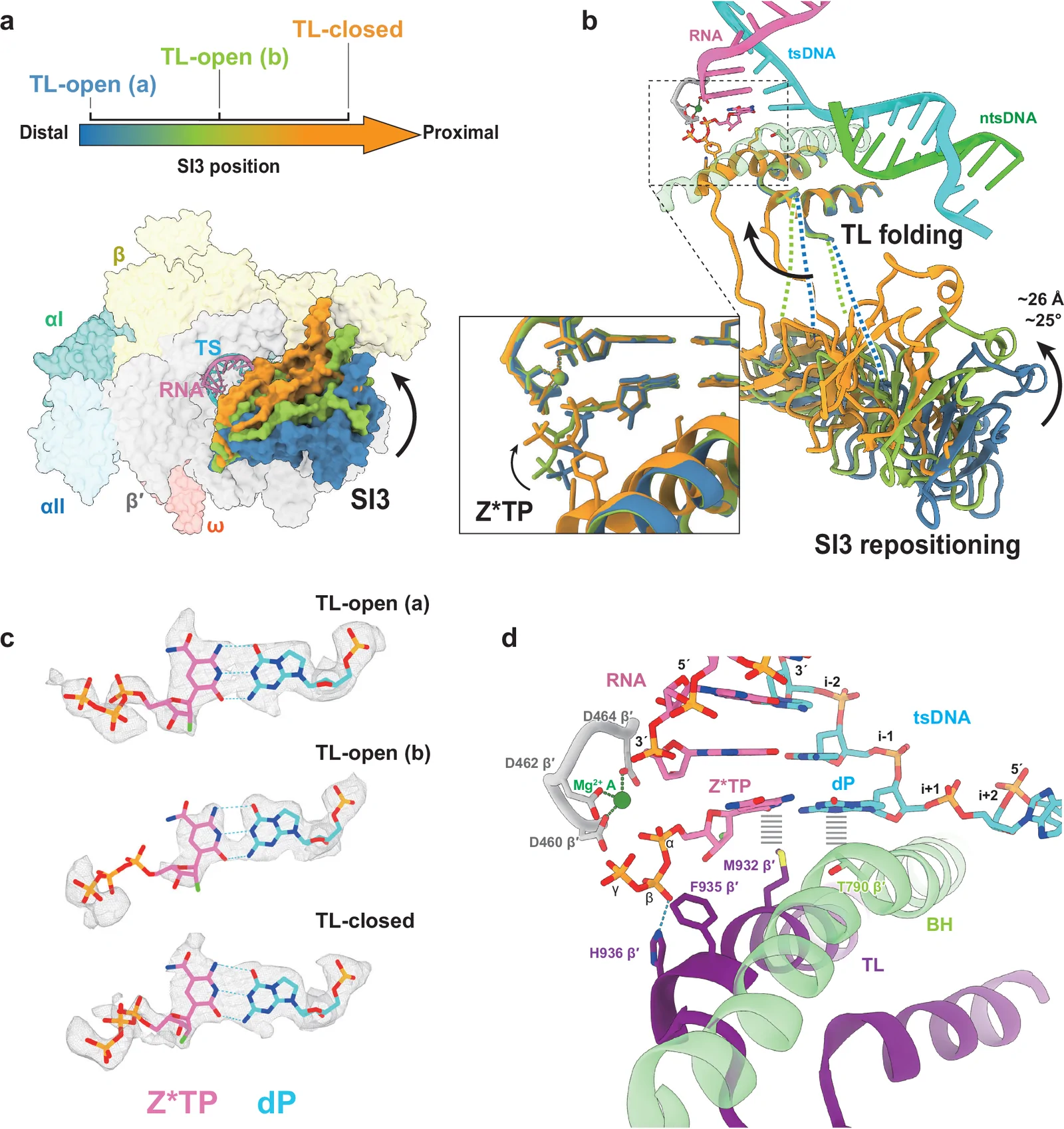
Cryo-EM structure of the *E. coli* RNA polymerase elongation complex containing dP:Z*TP. **a** Overview of the cryo-EM structures of the *E. coli* RNAP EC assembled with a dP template and the incoming Z*TP substrate. Three conformational states were resolved from this dataset based on the position of the SI3 domain and the conformation of the TL: TL-open (a) (steel blue), TL-open (b) (light green), and TL-closed (orange). The upper panel shows a conformational spectrum illustrating SI3 movement from a distal position (steel blue) to a proximal position (orange) relative to the active site, with the three structures mapped onto this trajectory. The lower panel shows the overall structure of the dP:Z*TP EC in the TL-closed state. The five RNAP subunits are rendered as transparent surfaces, colored and labeled as indicated. The three SI3 conformations corresponding to the states above are superimposed for comparison. **b** Structural comparison of the TL and SI3 domain among the three states. The active site of the TL-closed state is shown as a cartoon and includes RNA (hot pink), tsDNA (cyan), ntsDNA (lime), the P-loop (silver), the catalytic magnesium ion (green), the BH (transparent light green), TL, and SI3. TL and SI3 from all three states are superimposed using the color scheme from panel **a**. The inset highlights the conformation of the incoming Z*TP. Black arrows indicate the repositioning of SI3, TL folding, and loading of the Z*TP triphosphate moiety. **c** Base-pairing geometry of dP:Z*TP across all three states. In each structure, the template dP (cyan) forms a canonical Watson–Crick base pair with Z*TP. Atomic models are shown together with cryo-EM densities contoured at 6-σ. The fluorine atom at the C2′ position of Z*TP is colored in lime green. **d** Architecture of the active site in the TL-closed state of the dP:Z*TP EC. Key structural elements include RNA, tsDNA, the P-loop (silver), TL (purple), the catalytic magnesium ion (metal A, green), and BH (light green). Critical residues involved in substrate stabilization are shown as sticks: TL residues M932, F935, and H936, and BH residue T790. The hydrogen bond between H936 and the β-phosphate of Z*TP is shown as a blue dashed line. Hydrophobic interactions between TL/BH residues and nucleobase moieties are depicted as black bold dashed lines.

To further understand how the P:Z* base pair is recognized during transcription by a multi-subunit RNA polymerase, we applied the same strategy used for the dZ:PTP complex and reconstituted an *E. coli* RNAP EC containing a dP template paired with the incoming Z*TP nucleotide (Fig. 4; Supplementary Fig. 9; Supplementary Table 1). Because P is now on the template strand and Z* is the incoming nucleotide, this dataset provides a strand-switched counterpart to the dZ:PTP complex, enabling direct comparison of their substrate-recognition mechanisms.

In contrast to the relatively homogeneous TL conformation observed in dZ:PTP dataset, the dP:Z*TP dataset displayed substantial higher conformational heterogeneity in the trigger loop (TL) and SI3 domain (residues 943–1130 of β′), allowing us to resolve three distinct EC states (Fig. 4; Supplementary Fig. 9). Based on TL conformation, these structures were classified as TL-open (a) (2.60 Å), TL-open (b) (2.64 Å), and TL-closed (2.75 Å).

The three structures exhibit coordinated movements of the SI3 domain and the TL as substrate loading progresses (Fig. 4a, b). SI3 is a large insertion within the TL, and its repositioning reflects the early steps of TL engagement. SI3 resides in its most distal position relative to the active site in the TL-open (a) state. In the TL-open (b) state, SI3 shifts toward the active site, representing an intermediate position in the repositioning pathway while the TL remains fully open. SI3 then continues the repositioning event that occurs in concert with TL folding into helices. Across this transition—from the TL-open (a) state to the TL-closed state—SI3 rotates by ~25° and shifts by ~26 Å toward the active site. A similar SI3 repositioning pathway has been reported for RNAP ECs containing natural base pairs^16^, indicating that Z* does not perturb this native conformational mechanism.

Across all three states, Z*TP is clearly resolved in the active site and forms a Watson–Crick base pair with the templating dP (Fig. 4c). Remarkably, correct P:Z pairing is achieved even in the TL-open states, demonstrating that accurate base-pairing geometry forms early—prior to TL folding and independent of assistance from the closed TL conformation. As the TL transitions to the closed state, the triphosphate moiety of Z*TP undergoes accommodation and adopts the characteristic chair-like conformation also observed in the dZ:PTP structure (Fig. 4b, inset). Key TL residues (H936, F935, M932) adopt similar conformations as in natural systems and as in the dZ:PTP pre-catalytic complex, stabilizing the incoming Z*TP (Fig. 4d).

Together with the dZ:PTP dataset, these results demonstrate that both P:Z and P:Z* base pairs are recognized by *E. coli* RNAP in a manner highly similar to natural Watson–Crick pairs (Supplementary Fig. 7). Z*TP achieves correct pairing geometry even before TL folding, and the subsequent folding of the TL, repositioning of SI3, and accommodation of the triphosphate proceed following the same sequence of structural transitions described for native substrates. Thus, P:Z and P:Z* behave as native-like base pairs during the early steps of substrate loading, with the major distinction occurring later in the catalytic trajectory, as elaborated in the following section.

### A nitro-dependent π-hole interaction stabilizes BH bending and shifts the TL-folding equilibrium

**Fig. 5:**
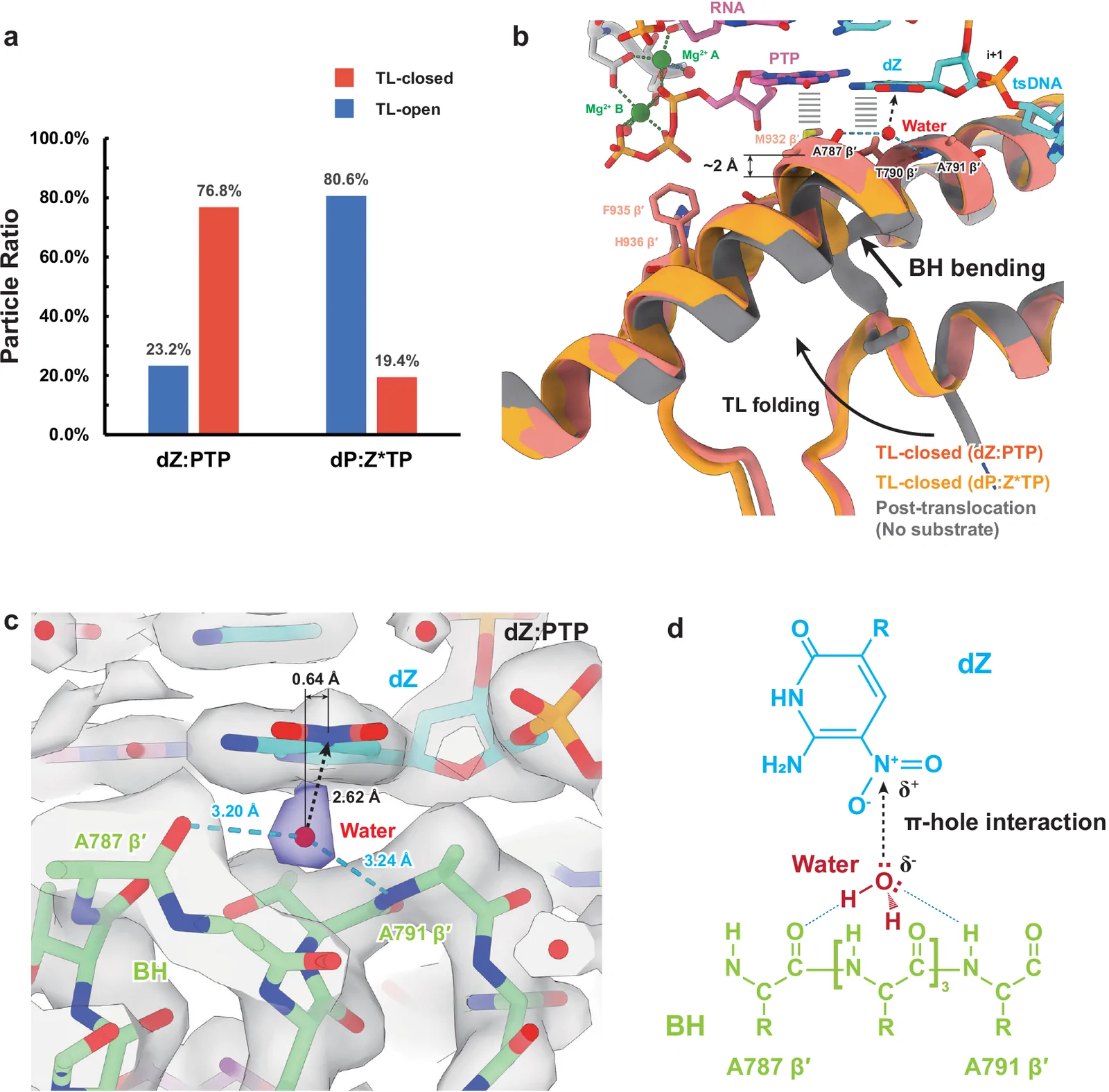
A nitro-dependent π-hole interaction stabilizes BH bending and shifts the TL-folding equilibrium. **a** Particle distributions of TL-open (steel blue) and TL-closed (orange red) states in the dZ:PTP and dP:Z*TP cryo-EM datasets. The y-axis indicates the fraction of particles in each TL conformation relative to the total particle set, and the x-axis corresponds to the two datasets. **b** Structural comparison of the bridge helix (BH) and trigger loop (TL) among three cryo-EM structures: the dZ:PTP EC (salmon), the TL-closed state of the dP:Z*TP EC (orange), and the post-translocated *E. coli* RNAP EC (gray; PDB: 8FVR^17^). In the absence of an incoming substrate in the post-translocated state, the BH adopts a straight conformation, whereas both TL-closed states exhibit a substantially bent BH. The maximal deviation of the bent BH—measured at residue A787—is ~2 Å relative to the straight BH (indicated). Surrounding active-site elements from the dZ:PTP EC are shown, including RNA (hot pink), tsDNA (cyan), the P-loop (silver), and two catalytic magnesium ions (green). The water molecule (chain T, residue 104 in the dZ:PTP EC structure determined in this study; PDB 9ZP4), which mediates the interaction between the dZ nitro group and BH, is shown as a red sphere. Hydrogen bonds between the water molecule and BH residues are shown as blue dashed lines. The π-hole interaction between the dZ nitro group and the water molecule is indicated by black dashed arrows, pointing from the water lone pair toward the electron-deficient nitro nitrogen. Black arrows depict the directions of TL folding and BH bending. **c** Detailed view of the water-mediated interaction between BH and the nitro group of dZ. The π-hole-interacting water molecule is highlighted as a red sphere. Hydrogen bonds and the π-hole interaction are shown as blue dashed lines and black dashed arrows, respectively. Distances for each interaction are labeled. The lateral displacement of the water molecule relative to the normal axis of the nitro plane is 0.64 Å. The color scheme for tsDNA and BH follows (**b**). Cryo-EM density (contoured at 6-σ) is shown and the density for the π-hole-interacting water molecule is highlighted in slate blue. **d** Chemical schematic illustrating the water-mediated network between the BH and the dZ nitro group, corresponding to the structural features shown in (**c**).

Comparison of the dZ:PTP and dP:Z*TP cryo-EM datasets revealed strikingly different particle distributions between TL-open and TL-closed states. In the dZ:PTP dataset, 76.8% of particles adopted the TL-closed conformation, whereas only 23.2% remained TL-open (Fig. 5a). In contrast, the dP:Z*TP dataset displayed the opposite trend: 80.6% of particles were TL-open and only 19.4% were TL-closed (Fig. 5a). These results demonstrate that the TL-open/closed equilibrium is markedly shifted between the two systems, with the dZ:PTP EC strongly favoring the TL-closed state. Single-turnover incorporation assays confirmed that this TL equilibrium shift has direct kinetic consequences: dZ:PTP supports substantially faster PTP incorporation than dP:Z*TP (Supplementary Fig. 2e, f), consistent with the interpretation that a higher proportion of TL-closed particles reflects more productive active-site closure. Furthermore, we found that the dZ scaffold supports measurably faster PTP incorporation than the dZ* scaffold under otherwise identical conditions (Supplementary Fig. 2c, d), indicating that the nitro group specifically contributes to the rate enhancement of PTP incorporation at the i + 1 position. This effect does not propagate to downstream elongation steps as we observed no measurable difference on the kinetics of subsequent nucleotide addition by chase assay (Supplementary Fig. 5b).

Furthermore, examination of the active sites across both TL-closed structures revealed that the dZ:PTP EC adopts a conformation much closer to the catalytic transition than any state captured in the dP:Z*TP dataset. The TL density in dP:Z*TP is noticeably weaker (Supplementary Figs. 6d, 9i), suggesting incomplete TL folding or increased conformational flexibility or heterogeneity. In addition, the catalytic metal coordination differs: in dP:Z*TP, only metal A is observed; the density corresponding to metal B is too weak to model reliably (Fig. 4c; Supplementary Figs. 6, 9). Moreover, the distance between metal A and the α-phosphate of Z*TP ( ~ 3.4 Å) is incompatible with coordination (Fig. 4d). These features indicate that, despite adopting a TL-closed conformation, the dP:Z*TP EC is not as catalytically advanced as the dZ:PTP EC. Together, these observations suggest that an additional stabilizing interaction is present in the dZ:PTP complex that promotes a more mature TL-closed configuration.

Closer inspection of the UBP environment at the i + 1 position revealed a previously undiscovered interaction present only in the dZ:PTP EC, mediated by a specifically positioned water molecule (chain T, residue 104 in the dZ:PTP EC structure determined in this study; PDB 9ZP4) (Fig. 5b, c). This water molecule bridges the bridge-helix (BH) residues A787 and A791 to the nitro group of dZ in the template strand, forming a three-way interaction network (Fig. 5b). This interaction helps stabilize the bent conformation of the BH, a conformation required to accommodate TL folding into the helical closed state.

Relative to the straight BH conformation seen in the post-translocated EC (PDB: 8FVR^17^), the BH in the dZ:PTP TL-closed structure is substantially bent, with the greatest deviation ( ~ 2 Å) occurring at A787 (Fig. 5b). Notably, the water molecule is positioned precisely at this hinge point, supporting the idea that it contributes to maintaining BH bending and thereby facilitates TL folding.

Detailed geometric analysis shows that this water molecule does not hydrogen-bond directly to the nitro group. Instead, it donates a lone pair toward the electron-deficient π-hole located above the nitro nitrogen (Fig. 5c, d). Nitro groups are known to generate a positive electrostatic region along the normal axis of the nitro plane, created by electron withdrawal through its two oxygen atoms^18^. In our structure, this π-hole-interacting water molecule lies 2.62 Å from the nitro nitrogen, with a lateral displacement of only 0.64 Å from the normal axis of the nitro plane, a geometry characteristic of π-hole interactions (Fig. 5c). In addition, the water molecule forms two hydrogen bonds with the backbone carbonyl of A787 and the backbone amide of A791, producing a highly stable three-directional network (Fig. 5c, d). In contrast to the dP:Z*TP EC, this interaction is entirely absent. At the position corresponding to the dZ nitro group, the C7 atom of dP lacks an electron-deficient substituent and does not engage in direct or water-mediated interactions.

It is important to note that the nitro-dependent π-hole interaction is unlikely to be the sole determinant of the TL-equilibrium shift. Other factors—such as differences in nucleobase size (e.g., the purine PTP in dZ:PTP potentially stacking more strongly with TL residue M932 than the pyrimidine Z*TP)—may also influence the equilibrium. However, the water-mediated π-hole interaction provides a compelling and structurally explicit mechanism by which template-base chemistry can stabilize BH bending and thereby promote TL folding.

These observations highlight that modifications at the C5 position of pyrimidines or the N7 position of purines on the template strand can modulate the TL-open/closed equilibrium of the RNAP EC. This provides a conceptual basis for designing nucleotide analogues to tune RNAP conformational dynamics and potentially regulate transcriptional outcomes.

## Discussion

### Transcription recognition of an eight-letter genetic alphabet by cellular RNA polymerases

The eight-letter “Hachimoji” genetic system represents a landmark expansion of the genetic alphabet^6^. Originally designed to strictly adhere to Watson–Crick geometric parameters through rearranged hydrogen-bonding patterns, these unnatural base pairs (UBPs)—including P:Z and B:S—have been successfully validated in DNA replication assays^11,19^. Furthermore, the orthogonality of these four independent base pairs has been shown in transcription driven by the single-subunit T7 RNA polymerase^6,8^, limited primarily by a mismatching of T/U with a minor tautomer of isoguanosine when it is used to implement the B hydrogen bonding pattern^20–22^. However, the transcription of Hachimoji UBPs by multi-subunit polymerases—the complex enzymatic machinery responsible for gene expression in all cellular life—has remained largely unexplored.

**Fig. 6:**
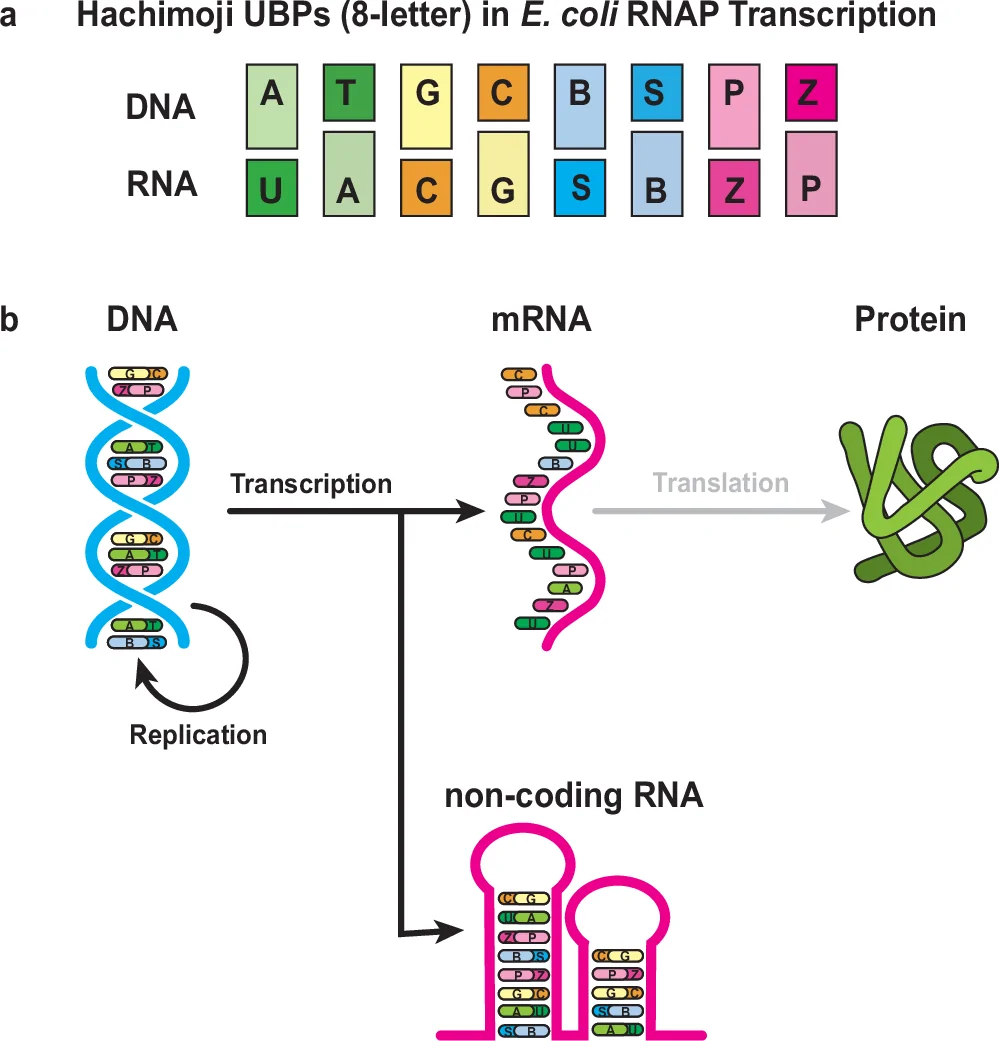
Integration of the Hachimoji eight-letter genetic system into *E. coli* RNA polymerase transcription. **a** The Hachimoji eight-letter genetic alphabet used in *E. coli* RNA polymerase transcription, consisting of the natural base pairs A:T (U) and G:C, and the unnatural base pairs P:Z and B:S. **b** Schematic overview of the transcriptional fate of Hachimoji DNA within a cellular-style central dogma framework. *E. coli* RNAP transcribes an eight-letter DNA template into an expanded RNA alphabet, generating either mRNA or non-coding RNA. The mRNA pathway (upper branch) represents the prerequisite step for the future goal of translating Hachimoji codons into protein, indicated by transparent arrows and labels to denote that translation has not yet been achieved. In contrast, the non-coding RNA pathway (lower branch) enables the design and evolution of functional RNAs—such as aptamers—leveraging the expanded chemical diversity of the eight-letter system. Replication of Hachimoji DNA is shown for conceptual completeness.

In this work, we expanded the investigation of the Hachimoji alphabet by incorporating the P:Z pair into the *E. coli* RNAP system. As illustrated in the upper panel of Fig. 6, we successfully established a robust 8-letter transcription system where *E. coli* RNAP reads a DNA template containing A, T, G, C, P, Z, B, and S, and effectively incorporates the corresponding RNA building blocks (U, A, C, G, Z, P, S, B). Together with our previous study^9^, our studies reveal that both B:S and P:Z pairs mimic the behavior of native base pairs: these UBPs form a standard Watson–Crick geometry within the active site and induce the TL to fold into its helical, catalytically active conformation to sense nucleotide loading. This confirms that the Hachimoji system is stereochemically compatible with the rigorous checkpoints of cellular transcription machinery. Beyond structural compatibility, kinetics measurements showed that P:Z incorporation rates are comparable to those of natural substrates (Supplementary Fig. 2), and NTP-chase assays demonstrated that *E. coli* RNAP resumes processive elongation efficiently after UBP incorporation (Supplementary Fig. 5), together establishing that the Hachimoji system meets the kinetic and mechanistic requirements of cellular transcription, not merely the geometric ones.

### Fidelity considerations and pK_a_ engineering in P:Z pairs

The construction of unnatural base pairs (UBPs) requires not only geometric compatibility but also high replication fidelity, which is often dictated by subtle physicochemical properties. Misincorporation represents a major and persistent challenge for AEGIS engineering. Designing a next-generation UBPs that reduces misincorporation has been a major ongoing effort.

For the P:Z pair, the key challenge arises from the intrinsic propensity of the Z base to mispair with guanine (G). This phenomenon is known to result from the Z heterocycle’s relatively low pK_a_ (approximately 7.8), caused by its electron-withdrawing nitro group. Under physiological pH, this favors the formation of the deprotonated anion Z^-^, which structurally mimics cytosine and leads to highly efficient G:Z^-^ mispairing^23^.

Our study decisively confirms that this deprotonation-driven mechanism is universal and enzyme-independent, extending the fidelity challenge from DNA replication (as shown previously) to the complex machinery of multi-subunit *E. coli* RNA Polymerase (RNAP) transcription. The observed misincorporation of GTP opposite the dZ template (and vice-versa) by *E. coli* RNAP confirms that the enzyme’s stringent active site checkpoints are insufficient to overcome this fundamental chemical vulnerability.

Importantly, we show here an effective strategy for improving Z fidelity through pK_a_ engineering. We employed the recently developed 2′-F-α-carboxamide-Z (Z*)^12^, in which the nitro group of Z is replaced by a carboxamide moiety. This modification raises the pK_a_ of the heterocycle to above 10, thereby suppressing formation of the mispair-prone deprotonated Z^-^ tautomer at neutral pH. As a result, Z* markedly reduces Z:G misincorporation during *E. coli* RNAP transcription. These findings indicate that tuning pK_a_ is an important and tractable strategy for optimizing the transcriptional fidelity of Z, particularly in cases where mispairing stems from deprotonation-driven tautomeric states. We view the present work (Z*) as an initial step toward a fully optimized P:Z pair, and further refinement of the Z scaffold will be required to achieve this goal.

An alternative strategy takes current knowledge of mismatched profile into consideration when we design new expanded genetic codons. Just like the natural genetic code, which allows multiple codons for the same amino acid (such as CUU, CUC, CUA, CUG for Leu), we could design XXS and XXU coded for same amino acid (given B can based pair with S or U). These experiments are underway.

### Exploiting π-hole interactions to tune multi-subunit RNAP trigger loop dynamics for expanded genetic alphabets

Our structures illuminate how chemical features at the i + 1 template position can directly influence the conformational landscape of multi-subunit RNA polymerase. In the dZ:PTP elongation complex (EC), we identified a previously undiscovered interaction network in which a precisely positioned water molecule bridges the backbone atoms of BH residues A787 and A791 to the nitro group of the dZ template. This interaction forms a three-way network consisting of two canonical hydrogen bonds to the BH and a π-hole interaction with the electron-deficient nitro nitrogen. Such π-hole contacts—which arise from the electropositive region above the nitro plane—are well described in protein–ligand complexes and frequently contribute to conformational preference and local rigidity^18^. Here, the π-hole geometry observed in our structure places the water molecule at the hinge of the BH bend, positioning it ideally to stabilize the bent-BH conformation that favors TL folding. This structural mechanism is supported by biochemical evidence: single-turnover kinetics measurements showed that the dZ scaffold supports faster PTP incorporation than the dZ* scaffold, and that dZ:PTP is markedly more efficient than dP:Z*TP (Supplementary Fig. 2c–f), directly linking the presence of the nitro-dependent π-hole interaction to enhanced catalytic output.

Beyond its structural role, this interaction reveals an unexpected recognition principle embedded within RNAP. Because the nitro-dependent π-hole acceptor lies at a position that corresponds to the C5 of pyrimidines and the N7 of purines, the same water molecule would engage with template purines differently. If the i + 1 base were a purine, its N7 could satisfy a direct hydrogen-bonding interaction with this water molecule, effectively generating an alternative BH-water-base network. In contrast, a canonical pyrimidine C5 lacks a comparable hydrogen-bond acceptor and therefore cannot form such a stabilizing bridge. This suggests that the polymerase is capable of sensing substituent identity at these positions, not through direct protein–nucleobase contacts, but through water-mediated interaction. The nitro group on Z thus acts as a chemically encoded handle that strengthens BH–template connectivity in a manner unavailable to natural pyrimidines.

Collectively, these results elevate the role of template-strand functional groups as active participants in RNAP conformational gating. Our findings demonstrate that introducing such functionalities—whether nitro groups, carboxamides, or other π-acidic motifs—can fine tune the conformational equilibrium of the EC by stabilizing specific RNAP microstates. This principle provides a conceptual framework for designing next-generation AEGIS nucleobases with tailored transcriptional behaviors. These insights also point toward rational design strategies for engineering UBP-specific polymerases.

### Future directions for an eight-letter genetic alphabet within the central dogma

Our findings extend the Hachimoji system beyond simplified single-subunit T7 models to multi-subunit polymerases that govern the cellular central dogma. This transition opens two distinct pathways for future applications (lower panel of Fig. 6).

The first pathway leads to mRNA and protein translation (gray arrow, Fig. 6). While translation of Hachimoji codons into proteins remains a future goal, our work establishes the essential prerequisite by showing that cellular RNA polymerase can synthesize Hachimoji mRNA. Notably, ribosomal incorporation of non-standard amino acids using an expanded genetic alphabet was achieved previously with the S:B pair^10^, but has not yet been demonstrated for the full eight-letter Hachimoji system.

The second pathway leads to functional non-coding 8-letter RNAs (ncRNAs), where the expanded alphabet enables the evolution of aptamer analogs (AegisBinders) with capabilities beyond those of standard 4-letter RNA. A prime example is the Hachimoji “Spinach” AegisBinder, which utilizes the expanded alphabet to bind fluorophores with high affinity, enabling enhanced cellular imaging^6^.

Furthermore, the potential for clinical application is supported by previous work where 6-letter AEGIS-DNA aptamers (incorporating P and Z) were engineered to target liver cancer cells^24^. This precedent suggests that analogous, high-affinity RNA AegisBinders can be developed using the Hachimoji system. By validating the synthesis of these transcripts in *E. coli* RNAP, our work lays out the foundation for evolving the next generation of catalytic and functional RNA molecules inside living systems.

## Methods

### Oligonucleotides and nucleotides used in this study

RNA oligonucleotides (5′-AUCGAGAGG), 3′-deoxy RNA oligonucleotides (5′-AUCGAGAG/3′dG/), template strand (ts) DNA oligonucleotides (5′-CCTTCTCTCTCTCGCTGATCCTCTCGATG), and non-template strand (nts) DNA oligonucleotides (5′-TCAGCGAGAGAGAGAAGG) were purchased from Integrated DNA Technologies (IDT). AEGIS ribotriphosphates (rPTP, rZTP, rBTP, rSTP and 2′-F-α-carboxamide-rZTP(rZ*TP)) were obtained from Firebird Biomolecular Sciences LLC (Alachua, FL). Standard phosphoramidites (Bz-dA, Ac-dC, dmf-dG and dT Phosphoramidite), dB phosphoramidite (dmf-isodG-CE Phosphoramidite), and controlled pore glass (CPG) with standard residues were purchased from Glen Research (Sterling, VA). AEGIS phosphoramidites (dP, dZ, dS and 2′-F-α-carboxamide-dZ (dZ*)) were also acquired from Firebird Biomolecular Sciences LLC (Alachua, FL) (chemical structures shown in Supplementary Fig. 3)

Oligonucleotides containing dP, dZ, dB and dS were synthesized on an ABI 394 DNA Synthesizer using standard phosphoramidite chemistry as previously reported^6^. To remove the NPE group on the Z nucleobase, the CPG supporting the synthetic oligonucleotides was treated with 2.0 mL of 1 M DBU in anhydrous acetonitrile at room temperature for 24 h. Following this, the CPGs were filtered, dried, and then treated with concentrated ammonium hydroxide at 55 °C for 16 h. After the ammonium hydroxide was removed, the oligonucleotides were purified via ion-exchange high-performance liquid chromatography (HPLC) and desalted using Sep-Pak® Plus C18 cartridges (Waters). Oligonucleotides containing 2′-F-α-carboxamide-dZ (dZ*) were synthesized on a Mermade 12 DNA Synthesizer using standard phosphoramidite chemistry. The CPG with the oligonucleotide containing dZ* was treated with 1.0 mL of 1 M DBU in anhydrous acetonitrile at room temperature for 24 h. The DBU/acetonitrile solution was removed from CPG. The CPG was dried and then treated with 1:1 solution of ammonium hydroxide:methylamine (40%). After the removal of volatiles, the oligonucleotides were purified via ion-exchange high-performance liquid chromatography (HPLC) with a mobile phase of 5 mM NaOH and 1.5 M sodium chloride gradient. The appropriate fractions were collected and desalted using Sep-Pak® Plus C18 cartridges (Waters).

### Purification of *E. coli* RNA polymerase

The expression plasmids for *E. coli* RNA polymerase, deposited by the Seth Darst lab, were purchased from Addgene, specifically the pEcRNAP6 (Addgene #128940) and pACYCDuet-LEc-rpoZ (Addgene #128837) plasmids^25^. *E. coli* RNA polymerase was overexpressed and purified following previously described methods^9^. Briefly, the two plasmids were co-transformed into BL21 (DE3) competent cells (Novagen). When the optical density (O.D.) reached 0.6, 0.25 mM IPTG was added to the cell culture, and protein expression was induced overnight at 20 °C. The cells were harvested and resuspended in Buffer A [50 mM Tris (pH 7.4), 500 mM NaCl, 5% (v/v) glycerol, 2 mM β-mercaptoethanol (BME), and 1 × EDTA-free protease inhibitor (Sigma-Aldrich)]. Cell lysis was performed using Microfluidics, followed by clarification through centrifugation. The target protein was purified from the cell lysate using NiNTA resin and eluted with 300 mM imidazole in Buffer A. To improve purity, the protein was further purified using a HiTrap Heparin column and an anion exchange column (HiTrap Q column). The fractions containing *E. coli* RNA polymerase (α2β′βω) were collected, combined, and concentrated. During concentration, the buffer was exchanged to the storage buffer [20 mM HEPES pH 8.0, 150 mM NaCl, 5% (v/v) glycerol, 1 mM DTT]. The final protein was flash-frozen in liquid nitrogen and stored at −80 °C for future use.

### In vitro transcription assay

Transcription assays were conducted using previously described methods with minor modifications^9,26^. A mini-scaffold containing 200 nM RNA (p^32^-labeled), 400 nM template strand DNA (tsDNA), and 600 nM non-template strand DNA (ntsDNA) was prepared in elongation buffer (EB) consisting of 20 mM Tris-HCl pH 8.0, 40 mM KCl, 5 mM DTT, and 5 mM MgCl_2_. The scaffold was annealed by heating at 80 °C for 5 min, then slowly cooled to room temperature for at least 2 h. To assemble the elongation complex, 240 nM *E. coli* RNAP and 40 nM mini scaffold were mixed in 1x EB and incubated at 30 °C for 20 min. The reaction was initiated by adding the elongation complex to each nucleotide triphosphate. Consequently, the reaction sample contained 20 nM mini-scaffold, 120 nM *E. coli* RNAP, and 20 μM of each nucleotide triphosphate in 1x EB. The reaction mixture was sampled by pipetting 1.5 μl of the sample into 6 μl of stop buffer [90% (v/v) formamide, 50 mM EDTA, 0.05% (w/v) xylene cyanol, and 0.05% (w/v) bromophenol blue] at time intervals of 0, 15 s, 1 min, 5 min, and 30 min. All samples were denatured by heating at 95 °C for 10 min and analyzed using 12% denaturing urea-PAGE gel. Band intensities were quantified from gel images using ImageJ (v6.0, NIH). For each lane, the intensity of the extension product band ( + 1 or higher) was divided by the sum of all band intensities in the lane to calculate incorporation efficiency (%). To account for batch-to-batch variation in RNAP activity, the raw incorporation efficiency of the cognate substrate at the 30 min time point was defined as 100%, and all values within each experimental replicate were normalized accordingly. All assays were performed in at least two independent replicates, and incorporation efficiency values are reported as mean ± standard deviation. For the detailed quantitative data and raw gel images, please refer to Source Data.

Single-turnover kinetics assays (k_obs_ measurement) were performed using the same elongation complex assembly procedure described above, with the following modifications. For comparison of incorporation rates across scaffold types (dP, dG, dZ, and dZ*), elongation complexes were treated with 0.1 μM of the indicated NTP substrate (ZTP, CTP, or PTP) and reactions were sampled at 0, 5, 10, 15, 30, 45, 60, 90 s and 2, 5, 10, 30 min. For determination of k_obs_ values for the cryo-EM scaffold-substrate pairs dZ:PTP and dP:Z*TP, elongation complexes were treated with 1 μM of the cognate NTP substrate. Given the substantially different incorporation rates of these two configurations, distinct time-course protocols were applied: reactions with dZ:PTP were sampled at 0, 5, 15, 25, 40, 60, 90 s and 2, 4, 8, 15, 30, 45, 60 min, while reactions with dP:Z*TP were sampled at 0, 2, 4, 8, 15, 30, 120, 240 min. All reactions were quenched in stop buffer, denatured at 95 °C for 10 min, and resolved by 12% denaturing urea-PAGE. Band intensities were quantified as described above, and the fraction of extended product was plotted as a function of time. k_obs_ values were derived by fitting the data to a single-exponential equation. For the detailed kinetic data, please refer to Source Data.

Chase assays were performed using elongation complexes assembled as described above. For the NTP-chase assay (Supplementary Fig. 5a), four scaffold-substrate configurations were tested: dZ:PTP, dP:ZTP, dZ*:PTP, and dP:Z*TP. For each configuration, the cognate UBP substrate was added to the elongation complex and incubated at 30 °C for 5 min to allow single-nucleotide extension at the i + 1 position. All four natural NTPs (ATP, GTP, CTP, and UTP) were then added as a chase and incubated for a further 5 min. Three samples were collected for each configuration: the initial elongation complex prior to substrate addition (S1), the complex after UBP incorporation (S2), and the complex after NTP chase (S3). For the UTP-chase assay (Supplementary Fig. 5b), three scaffold-substrate configurations were tested: dZ:PTP, dP:Z*TP, and dP:ZTP. For each configuration, the cognate UBP substrate was added and incubated for 5 min, after which UTP was added to a final concentration of 20 μM. Reactions were sampled at 15 s, 1, 5, and 30 min after UTP addition. For both assays, all samples were quenched in stop buffer, denatured at 95 °C for 10 min, and resolved by 12% denaturing urea-PAGE.

### Cryo-EM sample preparation

A mini-scaffold containing 3′-deoxy RNA, tsDNA, and ntsDNA in a molar ratio of 1.2:1:1.2 was annealed in 1× EB. To form the elongation complex, *E. coli* RNAP was mixed with the prepared mini scaffold in a molar ratio of 1:1.3 and incubated at room temperature for 30 min. The final concentration of *E. coli* RNAP was approximately 50 μM. After complex assembly, 5 mM MgCl_2_ and 8 mM CHAPSO were added. The detergent CHAPSO significantly improved the reduction of particle orientation bias^27^. Subsequently, 5 mM rPTP or 2′-F-α-carboxamide-rZTP (Z*TP) was mixed with the elongation complex, and the samples were kept at room temperature for 10 min. The samples were then kept on ice until they were applied to grids.

For grid freezing, 4 μl of the elongation sample with rPTP or 2′-F-α-carboxamide-rZTP was loaded onto a freshly plasma-cleaned (10 s, 100% argon) holey grid (Quantifoil® Carbon R 1.2/1.3, 2 nm Carbon (UTC) on Copper, mesh 300). The sample was incubated on the grids for 30 s before manually blotting for 6 s and subsequently plunged into liquid ethane. The vitrified grids were transferred to liquid nitrogen for storage and data collection.

### Cryo-EM data collection and processing

Both cryo-EM datasets for dZ:PTP and dP:Z*TP were collected at the Stanford-SLAC Cryo-EM Center. Both datasets were acquired using a Titan Krios transmission electron microscope (Thermo Fisher Scientific) operating at 300 keV and equipped with a Falcon 4i direct electron detector. Data collection was performed using the EPU (v3.3) software^28–31^. Finally, we collected 4128 images for dZ:PTP dataset and 12400 images for dP:Z*TP dataset without stage tilt. For the dZ:PTP dataset, we combined the dataset collected in this study with raw movies from our previously published structure (PDB entry: 8TXO; EMPIAR-11797)^32^, which contains 527 images with 60° stage tilt in total 1146 images (other 619 images are without stage tilt). Comprehensive imaging parameters for the dataset are summarized in Supplementary Table 1.

For the dZ:PTP complex, raw movies from two cryo-EM datasets were resampled to a pixel size of 0.83 Å/pixel, merged, and imported into CryoSPARC v4.5.3^33^. Motion correction and contrast transfer function (CTF) estimation were performed, followed by manual curation to remove low-quality micrographs (for example, those with CTF fit resolutions worse than 4.5 Å or relative ice thickness values outside 1.0–1.15). The curated micrographs were subjected to automated particle picking using the Blob Picker. Particles were extracted with a 400-pixel box size and Fourier-cropped to bin 4 (100-pixel box). Multiple rounds of 2D classification were performed, and classes containing well-defined *Ec*RNAP views were selected for ab-initio reconstruction and heterogeneous refinement. Particles from high-quality 3D classes were then selected for re-extraction at bin 1 using a 400-pixel box size. Several additional heterogeneous refinement steps were performed to further purify the dataset. A local 3D classification focused on the SI3 domain (mask applied around SI3; region highlighted in purple in Supplementary Fig. 6c) was used to separate *Ec*RNAP EC in different TL states. The best-resolved class representing the TL-closed state was subsequently refined by defocus refinement, global CTF refinement, and non-uniform refinement, yielding a 2.42 Å reconstruction.

For the dP:Z*TP dataset, image processing followed the same general workflow described for the dZ:PTP dataset in CryoSPARC, with several minor differences. After particle curation, refinement, and re-extraction at bin 1, a local 3D classification focused on the SI3–TL region was performed. In contrast to the single TL-closed state isolated from the dZ:PTP dataset, this classification revealed three distinct TL–SI3 conformational states: TL-open (a), TL-open (b), and TL-closed. Each class was independently subjected to defocus refinement, global CTF refinement, and non-uniform refinement. This yielded three final cryo-EM reconstructions at resolutions of 2.60 Å (TL-open a), 2.64 Å (TL-open b), and 2.75 Å (TL-closed).

### Model building

The atomic models for each conformation from both datasets were generated using a similar workflow. The published structure of the *Ec*RNAP elongation complex (PDB entry: 6RH3^34^) was used as the initial model and rigid body docked into the density maps using UCSF ChimeraX (v1.8)^35^. For the unnatural nucleotide triphosphates (rZ*TP and rPTP) and the unnatural DNA templates (dZ and dP), coordinate and restraint files were generated using Phenix Elbow (v1.21)^36^. The atomic models of UBP were fitted into the density maps and merged into the elongation complex model using Coot (v0.9.8)^37^. Water molecules were modeled following previously published procedures^38^. Briefly, sharpened cryo-EM maps were segmented using a 4-σ density threshold with the Segger plugin in UCSF Chimera (v1.17)^39^ to identify discrete density features. Candidate water sites were automatically detected using SWIM^38^, applying a distance criterion of 2.40 Å <x < 3.45 Å to nearby protein or nucleic-acid atoms. To minimize false positives and suppress noise, we applied a Q-score threshold of 0.7^40,41^ and required that each candidate peak be validated against both sharpened half-maps (half-A and half-B). Only peaks with Q-scores ≥0.7 consistently across all three maps (full map and two half-maps) were retained as water molecules. The final model was generated through iterative model adjustment in Coot and real-space refinement in Phenix with restraint weights. All structural figures were generated using ChimeraX.

Map-to-model FSC curves were computed for all four cryo-EM structures using phenix.mtriage (Phenix v1.21). For each structure, the sharpened full map, half-map A, half-map B, and the refined atomic model were provided as inputs, with the nominal resolution of each structure supplied as the resolution parameter. The resulting FSC curves are shown in Supplementary Fig. 10. Each panel displays the gold-standard FSC between the two half-maps and the map-to-model FSC against the sharpened map; the resolution at which the map-to-model FSC reaches 0.5 is reported for each structure.

### Reporting summary

Further information on research design is available in the Nature Portfolio Reporting Summary linked to this article.

## Supplementary information

## Source data

## Data Availability

All data supporting the findings of this study are available within the paper and its Supplementary Information. Source Data are provided with this paper. The cryo-EM density maps generated in this study have been deposited in the Electron Microscopy Data Bank under accession codes EMD-74516 (dZ:PTP EC), EMD-74513 (dP:Z*TP EC, TL-open (a)), EMD-74514 (dP:Z*TP EC, TL-open (b)), and EMD-74515 (dP:Z*TP EC, TL-closed). The corresponding atomic coordinates have been deposited in the Protein Data Bank (PDB) under accession codes 9ZP4 (dZ:PTP EC), 9ZP1 (dP:Z*TP EC, TL-open (a)), 9ZP2 (dP:Z*TP EC, TL-open (b)), and 9ZP3 (dP:Z*TP EC, TL-closed). All other materials, reagents, and data are available from the corresponding authors upon reasonable request. Source data are provided with this paper.
