# Supplementary material for: Structural basis of transcription of the hachimoji eight-letter alphabet by E. coli RNA polymerase
Source: Nat Commun. 2026 Sep 2;17:9060. doi: 10.1038/s41467-026-76668-0 (PMC13538358; doi:10.1038/s41467-026-76668-0)
Supplement: Supplementary file 1 [file 41467_2026_76668_MOESM1_ESM.pdf]

Supplementary information

Supplementary information: The online version contains supplementary material.

Structural Basis of Transcription of the Hachimoji Eight-Letter Alphabet by *E. coli* RNA Polymerase

Qingrong Li[1], Hyo-Joong Kim[2], Yan Liu[3], Juntaek Oh[1,10,11,12], Peini Hou[1], Shuichi Hoshika[2], Grigore Pintilie[3,4], Sriram Aiyer[5], Jenny Chong[1], Dmitry Lyumkis[5,6,7], Steven A. Benner[2*], Dong Wang[1,8,9*]

[1] *Department of Pharmaceutical Sciences, Skaggs School of Pharmacy and Pharmaceutical Sciences, University of California San Diego, La Jolla, CA 92093, USA.*

[2] *Foundation for Applied Molecular Evolution, 13709 Progress Blvd Box 7, Alachua, FL 32615, USA.*

[3] *Division of CryoEM and Bioimaging, Stanford Synchrotron Radiation Lightsource, SLAC National Accelerator Laboratory, Stanford University, Menlo Park, CA 94025, USA*

[4] *Department of Bioengineering, James H. Clark Center, Stanford University, Stanford, CA 94305, USA.*

[5] *The Salk Institute for Biological Studies, La Jolla, CA 92037, USA.*

[6] *Department of Integrative Structural and Computational Biology, The Scripps Research Institute 10550 N Torrey Pines Road, La Jolla, CA 92037, USA.*

[7] *Graduate School of Biological Sciences, Section of Molecular Biology, University of California San Diego, La Jolla, CA 92093, USA.*

[8] *Department of Molecular and Cellular Medicine, School of Medicine, University of California San Diego, La Jolla, CA 92093, USA.*

[9] *Department of Biochemistry and Molecular Biophysics, University of California San Diego, La Jolla, CA 92093, USA.*

[10] *Current Address: Department of Regulatory Science, Graduate School, Kyung Hee University, Seoul, 02447, Republic of Korea*

[11] *Current Address: Institute of Regulatory Innovation through Science, Kyung Hee University, Seoul, 02447, Republic of Korea*

[12] *Current Address: Institute of Integrated Pharmaceutical Sciences. Kyung Hee University, Seoul, 02447, Republic of Korea*

[*]Corresponding authors: sbenner@ffame.org, dongwang@ucsd.edu

Supplementary Figures 1 to 10

Supplementary Figure 1 (related to Fig. 1b)

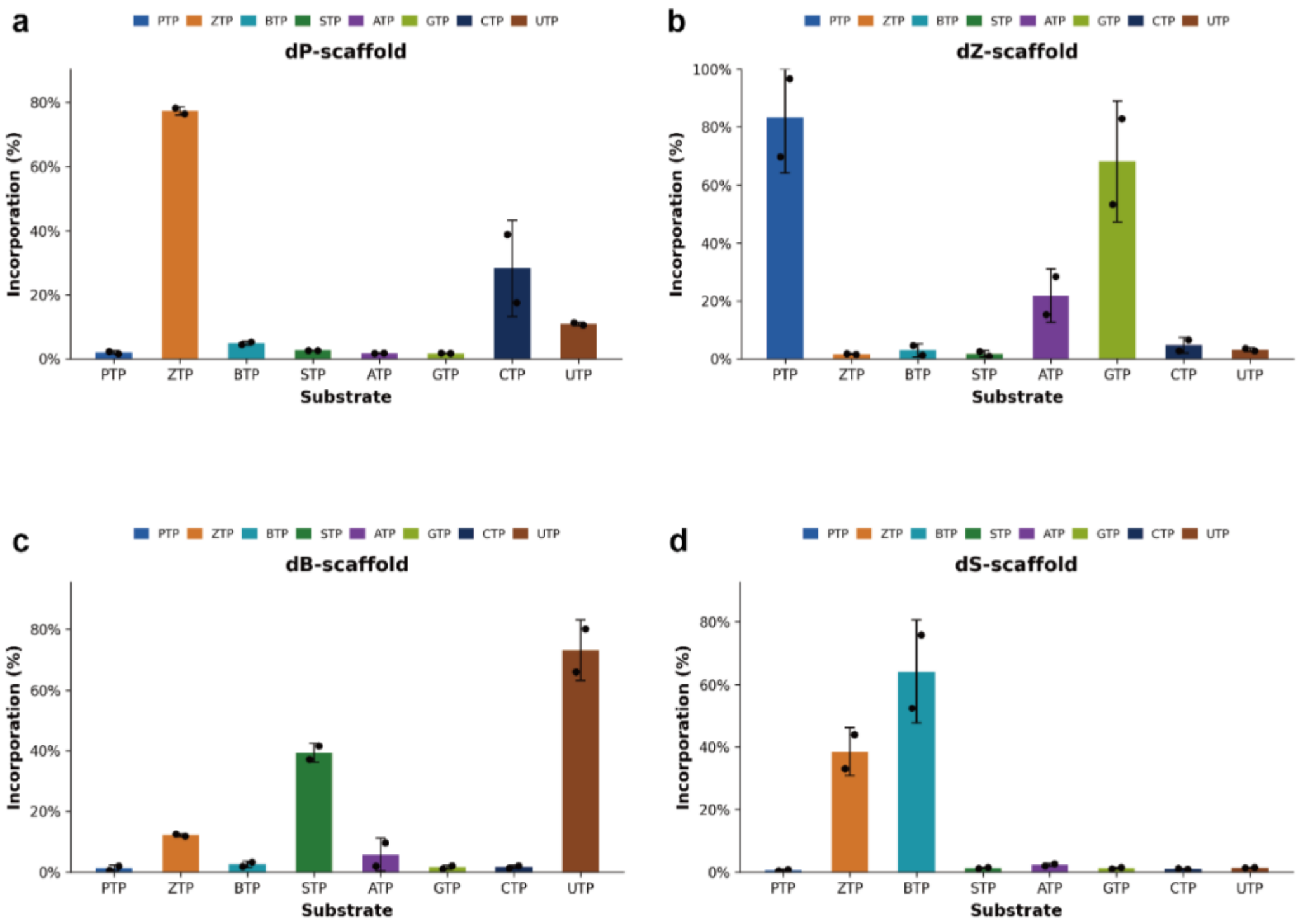

Supplementary Figure 1. Incorporation efficiency of all eight Hachimoji NTP substrates across four UBP template scaffolds.

a–d. Band intensities from single-nucleotide transcription assays (Fig. 1b) were quantified and displayed as bar plots for each template scaffold: dP (**a**), dZ (**b**), dS (**c**), and dB (**d**). Incorporation efficiency (%) was calculated from band intensities at the 15-s time point, at which substrate discrimination between cognate and non-cognate nucleotides is most pronounced, before misincorporation products have accumulated to significant levels. The eight NTP substrates are shown along the x-axis and colored as indicated in the legend. Data are presented as mean ± SD from two independent experiments (n = 2).

Supplementary Figure 2 (related to Figs. 1, 2)

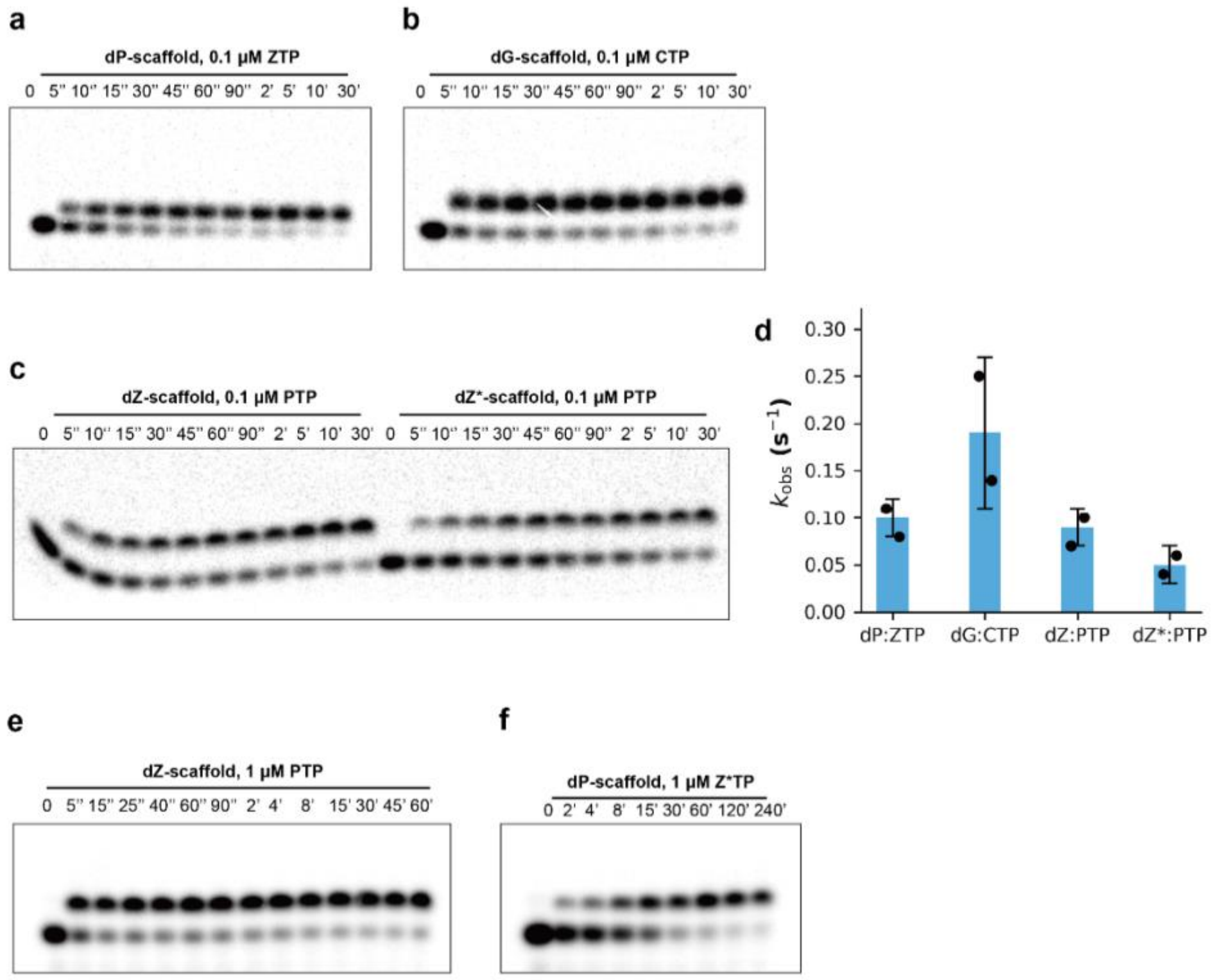

Supplementary Figure 2. Single-nucleotide incorporation kinetics for dP, dZ, dZ* and dG scaffolds. a–c Single-nucleotide incorporation assay measuring observed rate constants (k_{obs}) for four scaffold-substrate combinations. *E. coli* RNAP elongation complexes were assembled on scaffolds containing dP (**a**), dG (**b**), dZ or dZ* (**c**) at the template i+1 position and treated with 0.1 μM of the indicated NTP substrate (ZTP, CTP, or PTP). Reactions were sampled at 0 s, 5 s, 10 s, 15 s, 30 s, 45 s, 60 s, 90 s, 2 min, 5 min, 10 min, and 30 min, resolved by 12% denaturing urea-PAGE, and visualized by autoradiography of p^{32}-labeled RNA. These assays were performed in two independent replicates. **d** Bar plot of k_{obs} (s^{-1}) was derived by single-exponential fitting (Panel **a–c**). Data are presented as mean ± SD from two independent experiments (n = 2). **e–f** Single-nucleotide incorporation assay measuring k_{obs} for the dZ:PTP (**e**) and dP:Z*TP (**f**) scaffold-substrate pairs used in the cryo-EM elongation complexes reported in this study. *E. coli* RNAP elongation complexes were assembled on scaffolds containing dZ or dP at the template i+1 position and treated with 1 μM PTP or Z*TP, respectively. Given their differing incorporation rates, distinct time-course protocols were applied: reactions with dZ:PTP were sampled at 0 s, 5 s, 15 s, 25 s, 40 s, 60 s, 90 s, 2 min, 4 min, 8 min, 15 min, 30 min, 45 min, and 60 min, while reactions with dP:Z*TP were sampled at 0 s, 2 min, 4 min, 8 min, 15 min, 30 min, 120 min, and 240 min. These assays were performed in two independent replicates.

Supplementary Figure 3 (related to Fig. 2a)

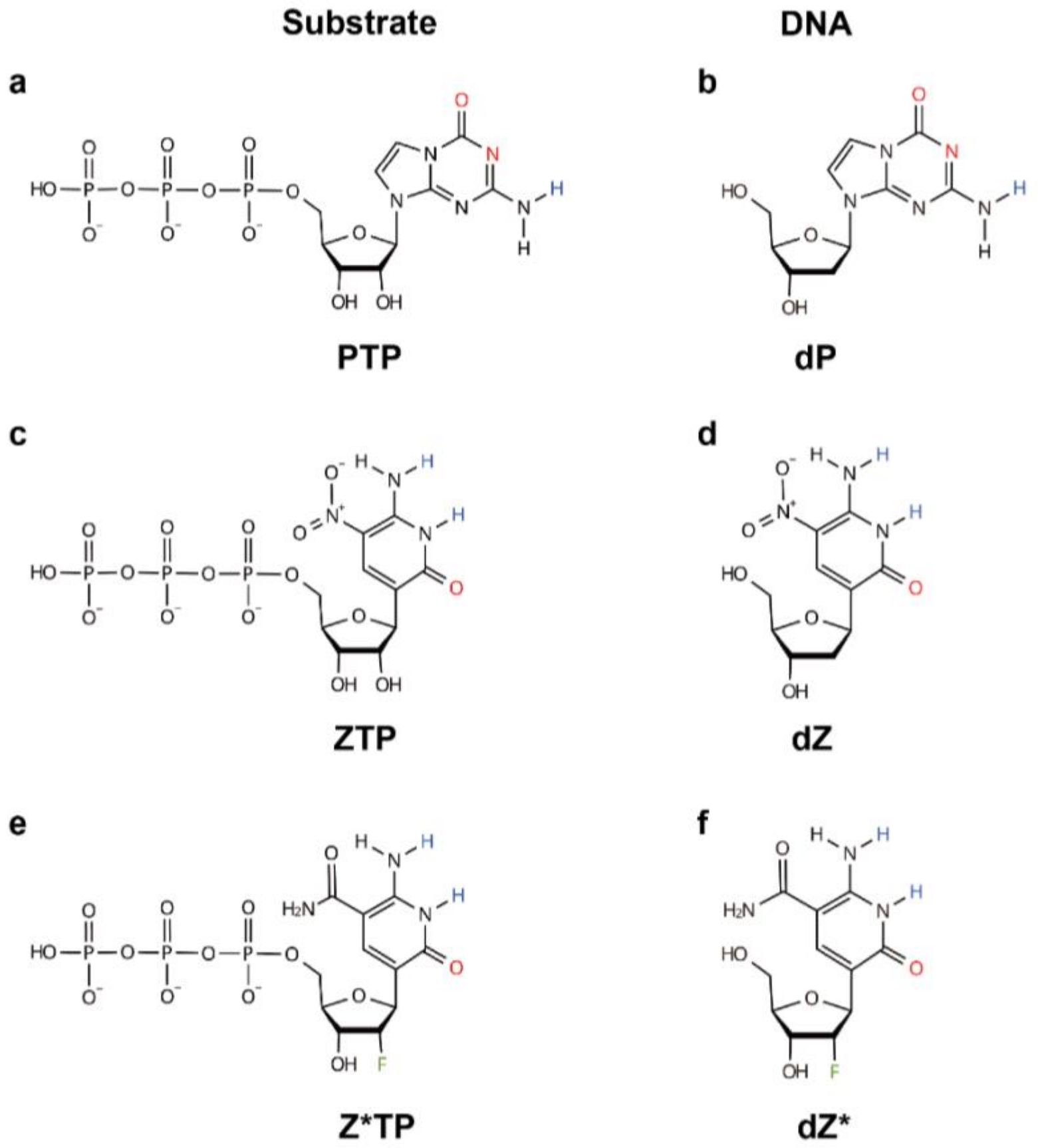

Supplementary Figure 3. Chemical structures of the unnatural nucleobases P, Z, and Z* in their NTP substrate and template DNA forms. a, c, e Complete chemical structures of the unnatural ribonucleoside triphosphates used as NTP substrates in this study: PTP (**a**), ZTP (**c**), and Z*TP (**e**). **b, d, f** Complete chemical structures of the corresponding template DNA nucleosides: dP (**b**), dZ (**d**), and dZ* (**f**). Hydrogen-bond donors and acceptors for Watson-Crick base pairing are colored blue and red, respectively. The 2′-F substituent of Z* is highlighted in green.

Supplementary Figure 4 (related to Fig. 2b)

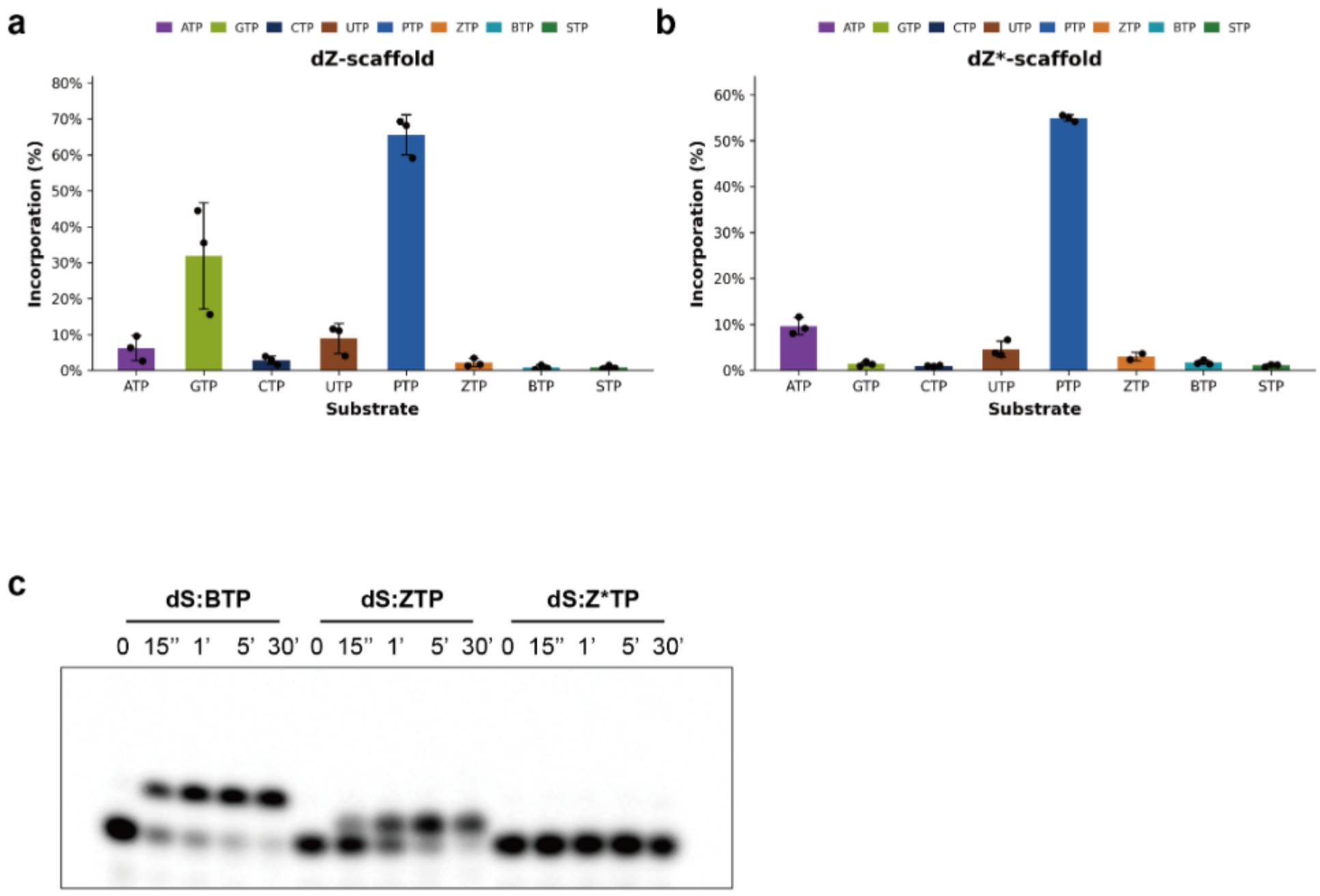

Supplementary Figure 4. Comparison of incorporation efficiency between dZ and dZ* scaffolds. a, b Incorporation rates were quantified from single-nucleotide transcription assays of Fig. 2b. Brackets connect the cognate substrate to the two NTPs with the highest misincorporation at 10 s. Data are presented as mean ± SD from three independent experiments (n = 3). **c** Single-nucleotide incorporation assay comparing BTP, ZTP, and Z*TP on the dS scaffold at the template i+1 position. *E. coli* RNAP elongation complexes were assembled on the dS scaffold and treated with 20 μM of each indicated substrate. Reactions were sampled at 15 s, 1 min, 5 min, and 30 min, resolved by 12% denaturing urea-PAGE, and visualized by autoradiography of p^{32}-labeled RNA. All assays were performed in two independent replicates.

Supplementary Figure 5 (related to Figs. 1 and 2)

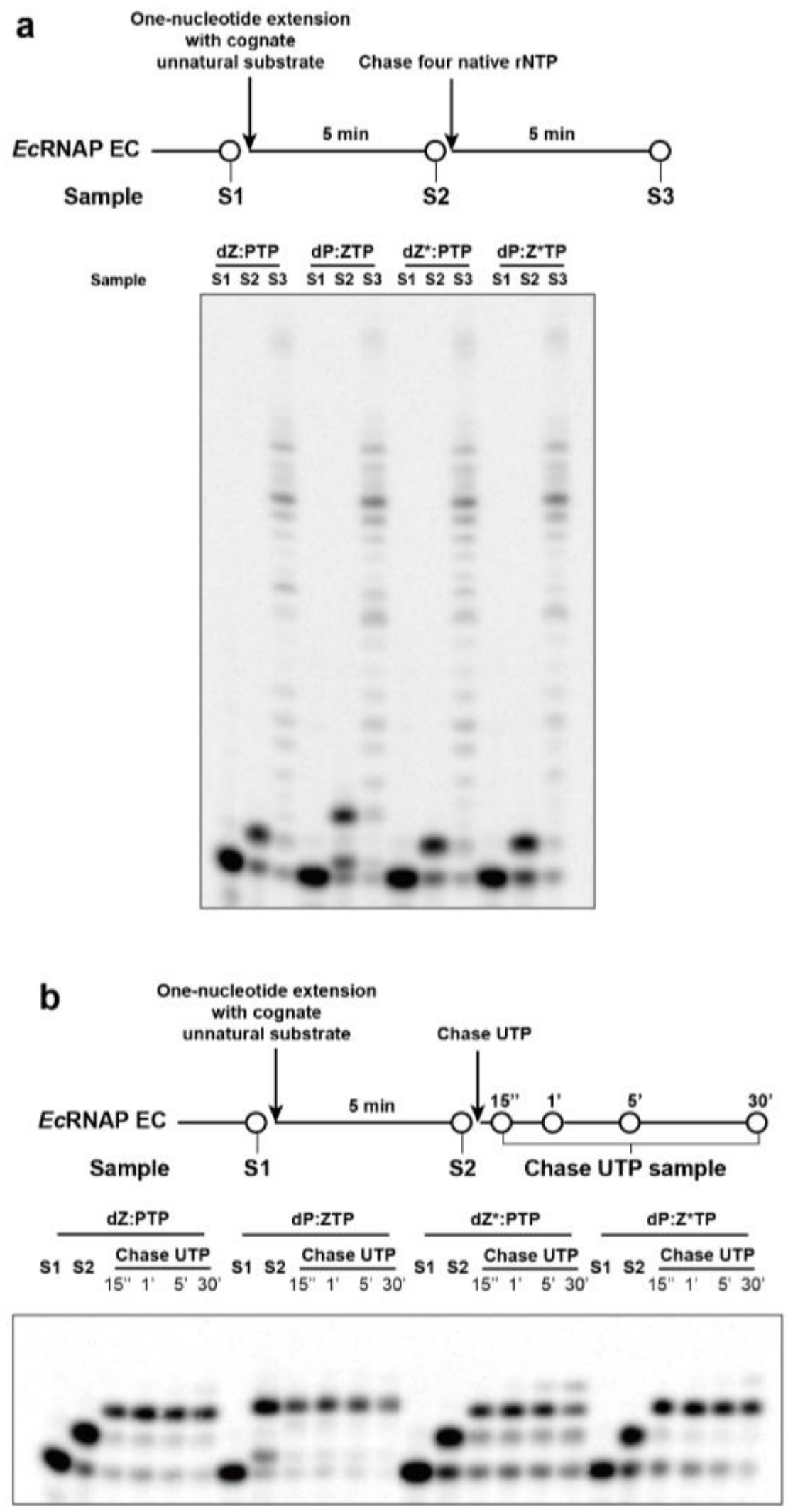

Supplementary Figure 5. NTP-chase assay demonstrating RNAP translocation and continued elongation after UBP incorporation. a *E. coli* RNAP elongation complexes were assembled on scaffolds containing dZ, dP, or dZ* at the template i+1 position. Four scaffold-substrate combinations were tested, including dZ:PTP, dP:ZTP, dZ*:PTP, and dP:Z*TP. For each configuration, the cognate UBP substrate was first added and incubated for 5 min to allow single-nucleotide extension at the i+1 position. All four natural NTPs (ATP, GTP, CTP, and UTP) were then added as a chase and incubated for a further 5 min. Three samples were collected for each scaffold-substrate pair: the initial elongation complex prior to substrate addition (S1), the complex after UBP incorporation (S2), and the complex after NTP chase (S3). Samples were resolved by 12% denaturing urea-PAGE and visualized by autoradiography of p^{32}-labeled RNA. The upper panel shows a schematic of the experimental procedure, and the lower panel shows representative gel images for all four scaffold-substrate configurations. **b** A single-nucleotide UTP-chase assay, performed under similar conditions to panel A, was conducted on three scaffold-substrate configurations (dZ:PTP, dP:ZTP, dZ*:PTP, and

dP:Z*TP). The dZ:PTP configuration generates a π-hole interaction via the dZ nitro group; the dP:ZTP, dP:Z*TP and dZ*:PTP configurations use dP and dZ* as the template base, in which this interaction is absent, and serve as controls. For each configuration, the cognate UBP substrate was first added and incubated for 5 min to allow single-nucleotide extension. UTP was then added to a final concentration of 20 μM and reactions were sampled at 15 s, 1 min, 5 min, and 30 min. Six samples were collected per configuration: the initial elongation complex prior to substrate addition, the complex after UBP incorporation, and the complex at each of the four UTP-chase time points. Samples were resolved by 12% denaturing urea-PAGE and visualized by autoradiography of p^{32}-labeled RNA. All assays were performed in two independent replicates.

Supplementary Figure 6 (related to Fig. 3)

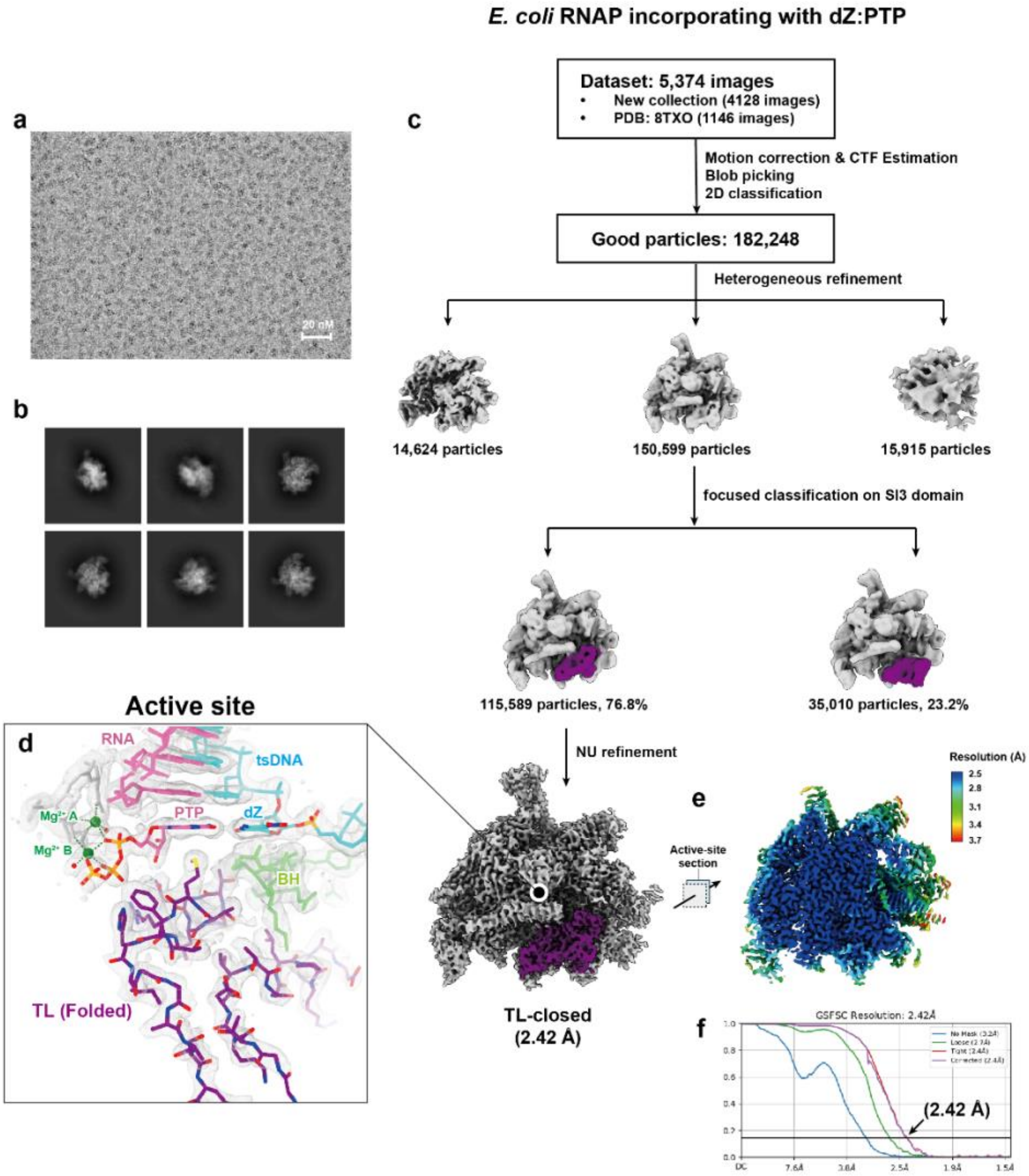

Supplementary Figure 6. Cryo-EM data processing of the *E. coli* RNA polymerase elongation complex containing dZ:PTP. a Representative raw micrographs of the *E. coli* RNAP EC containing dZ:PTP. **b** Reference-free 2D class averages of the *E. coli* RNAP EC containing dZ:PTP. **c** Cryo-EM data processing workflow for the *E. coli* RNAP EC. The SI3 domain is highlighted in purple. **d** Detailed view of the active site of the *E. coli* RNAP EC containing dZ:PTP. Key elements include RNA (hot pink), tsDNA (cyan), the P-loop (silver), TL (purple), two catalytic magnesium ions (green), and the bridge helix (BH; light green). The cryo-EM density is shown as a silver mesh contoured at 8-σ. **e** Final cryo-EM map of the *E. coli* RNAP EC, colored by local resolution. The map is displayed in a cross-sectional view through the active site to reveal internal structural features. **f** Gold-standard Fourier shell correlation (FSC) curves for the masked core region of the *E. coli* RNAP EC reconstruction.

Supplementary Figure 7

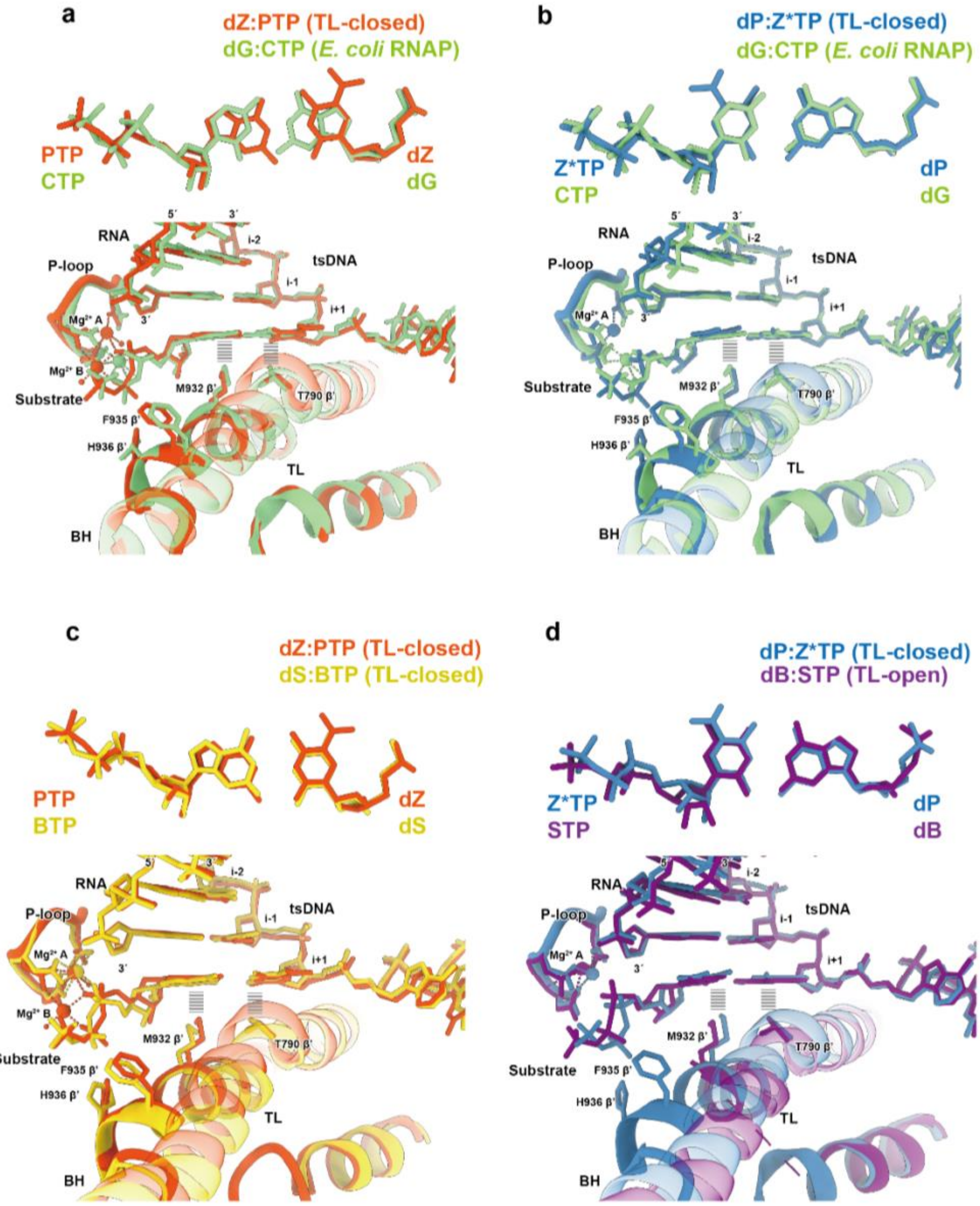

Supplementary Figure 7. Structural comparison between UBP-containing elongation complexes and the native *E. coli* RNAP EC at the active site. a Comparison of the active-site architecture between the dZ:PTP EC (orange red) and the native dG:CTP EC (light green; PDB: 6RH3[1]). Key structural elements—RNA, tsDNA, P-loop, trigger loop (TL), catalytic magnesium ions, and BH—are shown to illustrate the high degree of structural conservation between the UBP-containing and native complexes. **b** Comparison of the active site between the TL-closed state of the dP:Z*TP EC (steel blue) and the native dG:CTP EC (light green; PDB: 6RH3[1]). Despite the presence of the unnatural Z*TP substrate, the overall configuration of the active site remains closely aligned with that of the native complex.

Supplementary Figure 8

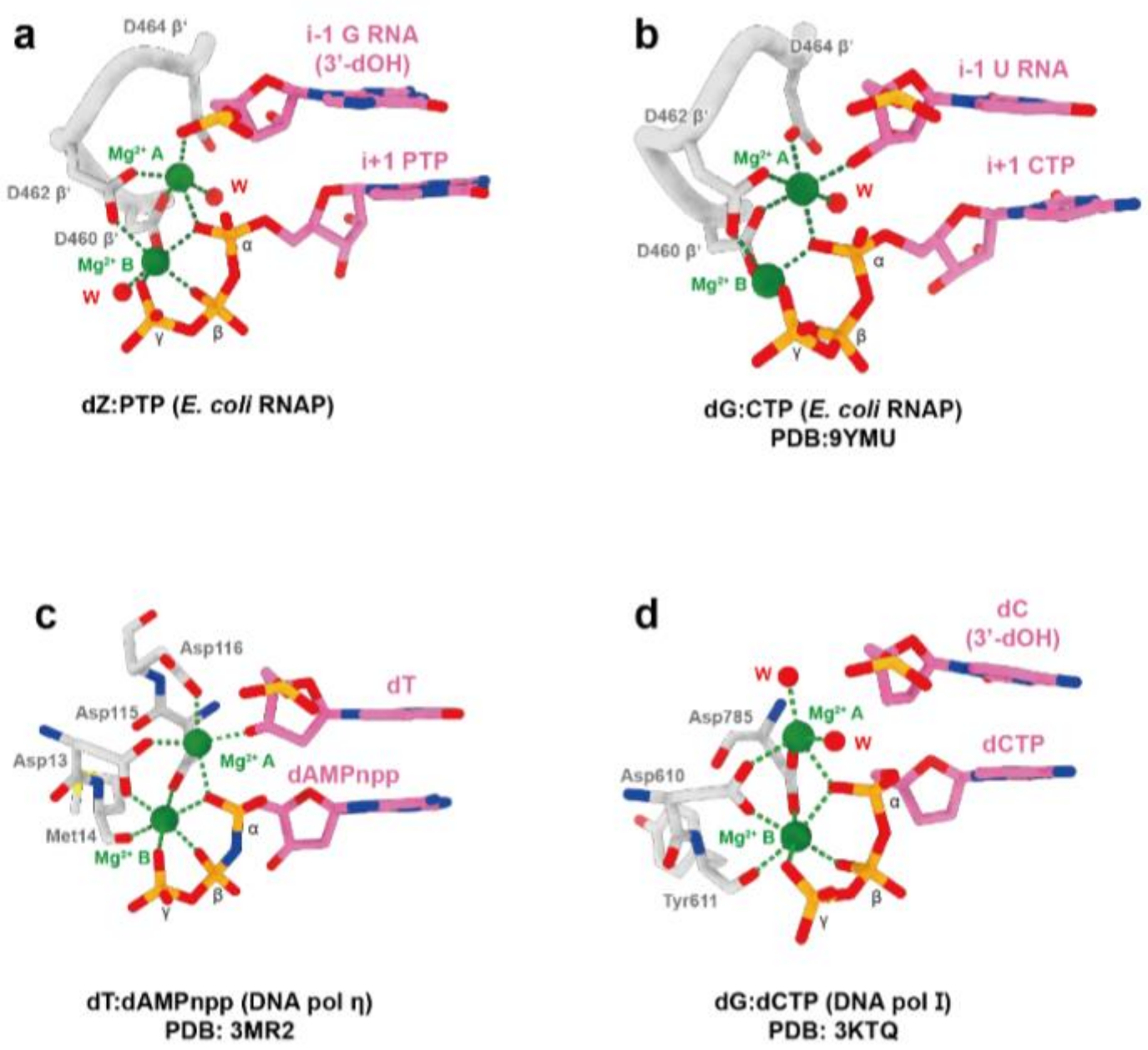

Supplementary Figure 8. Structural comparison between UBP-containing *E. coli* RNAP EC and DNA polymerases in two-metal-ion coordination. a, b Two-metal-ion coordination in the *E. coli* RNAP EC containing dZ:PTP and native dG:CTP (PDB: 9YMU[2]) at the i+1 position. Two catalytic Mg^{2+} ions (green) are coordinated by the three conserved aspartate residues of the P-loop (silver), two ordered water molecules (red), and the triphosphate moiety of the incoming NTP (hot pink). **c, d** Two-metal-ion coordination in DNA polymerase η (PDB: 3MR2[3]) (panel **c**) and DNA polymerase I (PDB: 3KTQ[4]) (panel **d**). Coordinating polymerase residues are shown as pink sticks, and DNA strands are colored yellow. Ordered water molecules are depicted as red spheres. In all panels, coordination bonds to Mg^{2+} are indicated by green dashed lines. The labels "α," "β," and "γ" denote the positions of the phosphate groups of the incoming nucleotide.

Supplementary Figure 9 (related to Fig. 4)

\

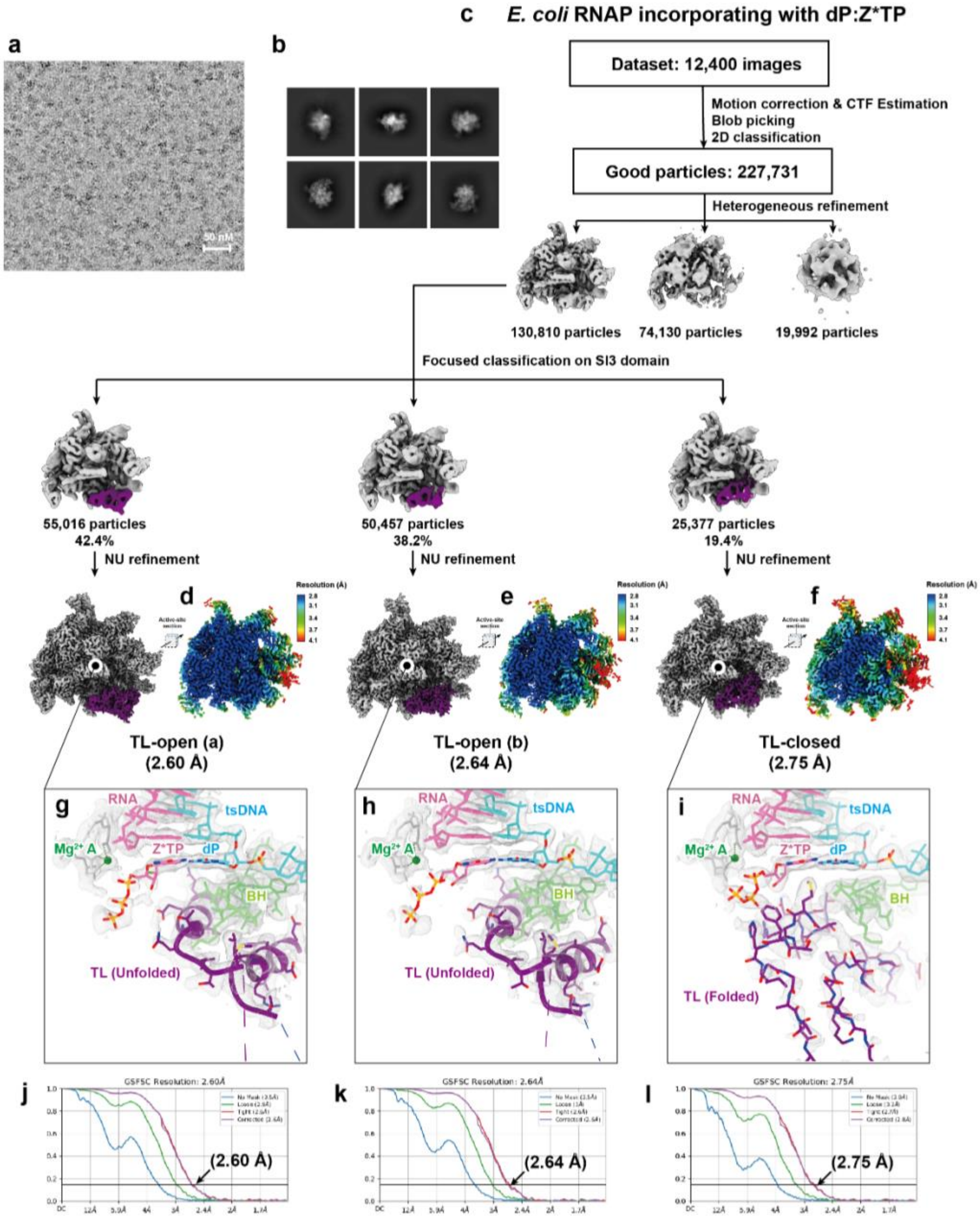

Supplementary Figure 9. Cryo-EM data processing of the *E. coli* RNA polymerase elongation complex containing dP:Z*TP. a Representative raw micrographs of the *E. coli* RNAP EC containing dP:Z*TP. **b** Reference-free 2D class averages of the *E. coli* RNAP EC containing dP:Z*TP. **c** Cryo-EM data processing workflow for the *E. coli* RNAP EC. The SI3 domain is highlighted in purple. Three conformational states—TL-open (a), TL-open (b), and TL-closed—were resolved from this dataset. **d–f** Final cryo-EM maps of the *E. coli* RNAP EC for the three states: TL-open (a) (panel **d**), TL-open (b) (panel **e**), and TL-closed (panel **f**). Each map is colored according to local resolution

and displayed in a cross-sectional view through the active site to reveal internal structural features.

g–i. Detailed active-site views of the *E. coli* RNAP EC containing dP:Z*TP for the three states: TL-open (a) (panel **g**), TL-open (b) (panel **h**), and TL-closed (panel **i**). Key structural elements include RNA (hot pink), tsDNA (cyan), P-loop (silver), TL (purple), catalytic magnesium ion (green), and BH (light green). The fluorine atom at the C2′ position of Z*TP is colored in lime green. Cryo-EM densities are shown as silver meshes contoured at 3.5-σ. **j–l** Gold-standard Fourier shell correlation (FSC) curves for masked core regions of the *E. coli* RNAP EC reconstructions corresponding to TL-open (a) (panel **J**), TL-open (b) (panel **k**), and TL-closed (panel **l**).

Supplementary Figure 10

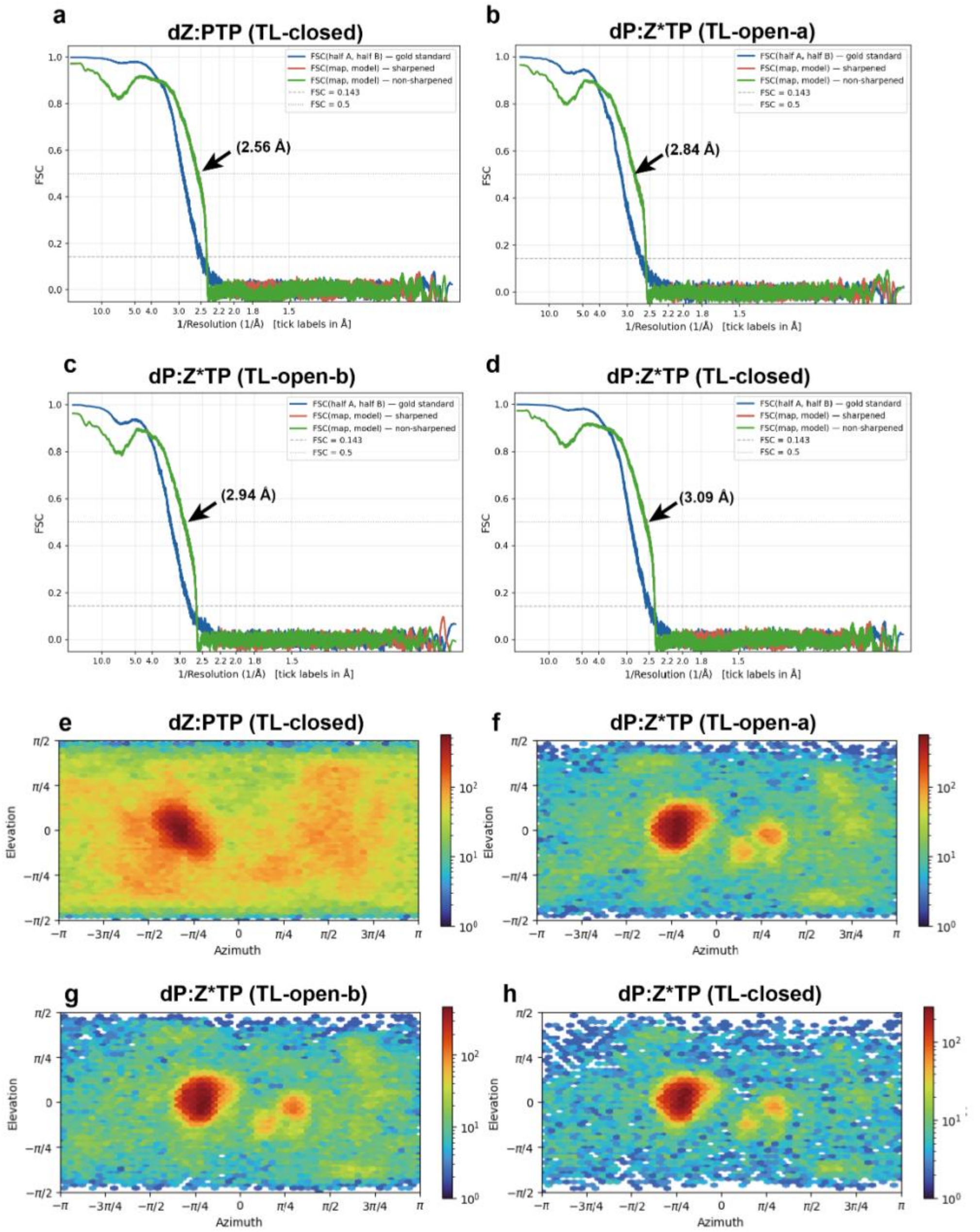

Supplementary Figure 10. Map-to-model Fourier shell correlation curves for the four cryo-EM structures. FSC curves were computed using phenix.mtriage. Each panel shows three curves: FSC (half A, half B) in blue, representing the gold-standard resolution estimate derived from two independently refined half-maps; FSC (map, model) for the sharpened map in red; and FSC (map, model) for the non-sharpened map in green. Horizontal lines indicate FSC thresholds of 0.143 (dashed) and 0.5 (dotted). The x-axis shows spatial frequency in 1/Å, with tick labels indicating the corresponding resolution in Å. **a** Map-to-model FSC for the dZ:PTP EC (TL-closed state). The map-to-model FSC (map, model) reaches 0.5 at 2.58 Å. **b** Map-to-model FSC for the dP:Z*TP EC in the TL-open (a) state. The map-to-model FSC (map, model) reaches

0.5 at 2.84 Å. **c** Map-to-model FSC for the dP:Z*TP EC in the TL-open (b) state. The map-to-model FSC (map, model) reaches 0.5 at 2.94 Å. **d** Map-to-model FSC for the dP:Z*TP EC in the TL-closed state. The map-to-model FSC (map, model) reaches 0.5 at 3.09 Å. **e–h** Angular distribution plots for the cryo-EM reconstructions of dZ:PTP (TL-closed), dP:Z*TP (TL-open-a), dP:Z*TP (TL-open-b), and dP:Z*TP (TL-closed), respectively. The plots were generated in cryoSPARC from the final particle sets used for each reconstruction. The horizontal and vertical axes represent azimuth and elevation, respectively. The color scale indicates the number of particle views contributing to each angular bin.

Supplementary Table 1

Cryo-EM data collection, refinement and validation statistics

**Dataset**	**dZ:PTP**			**dP:Z*TP**		
**States**	TL-closed			TL-open (a)	TL-open (b)	TL-closed
**PDB**	9PZ4			9PZ1	9PZ2	9PZ3
**EMDB**	EMD-74516			EMD-74513	EMD-74514	EMD-74515
Data source	EMPIAR-11797 (PDB: 8TXO)		New collection	New collection		
Microscope	FEI Titan Krios		Titan Krios G3i 300keV	Titan Krios G3i 300keV		
Detector	Gatan K3 BioQuantum		Falcon 4i Ceta 16M Ceta 2	Falcon 4i Ceta 16M Ceta 2		
Cs (mm)	2.7	2.7	2.7	2.7		
Pixel size (Å)	0.83	0.83	0.743	0.743		
Electron dose (e-/Å^2)	34.2	38.8	30	30		
Defocus range (µm)	-0.9 to -1.7	-1.1 to -2.5	-1.0 to -2.5	-1.0 to -2.5		
Tilt angle (°)	0	60	0	0		
Micrograph Number	619	527	4,128	12,400		
**Reconstruction**						
Software	cryoSPARC-4.5					
Particles refinement	115,589			55,016	50,457	25,377
Symmetry	C1			C1	C1	C1
Resolution (Å)	2.42			2.60	2.64	2.75
B-factor (Å^2)	61.6			74.5	76.1	68.2
**Refinement**						
**Model composition**						
Number of atoms	26,654			26,021	25,960	26,086
Protein residues	3214			3191	3193	3215
Nucleotides	41			37	37	37
Ligand	1 (PTP)			1 (Z*TP)	1 (Z*TP)	1 (Z*TP)
**Model resolution**						
0.5 FSC (Å)	2.56			2.84	2.94	3.09
**Bonds RMSD**						
Bonds lengths (Å)	0.0070			0.0053	0.0064	0.0056
Bonds angles (°)	0.58			0.67	0.77	0.68
**Validation**						
MolProbity score	1.23			1.45	1.51	1.54
Clash score	4.05			5.27	6.07	5.66
Rotamer outliers	0.07%			0.26%	0.26%	0.37%
C-beta outliers	1			3	0	1
**Ramachandran plot**						
Favored	97.81%			97.07%	96.98%	96.50%
Allowed	2.13%			2.87%	2.93%	3.34%
Outlier	0.06%			0.06%	0.09%	0.16%

Supplementary references

1. Abdelkareem, M. *et al.* Structural Basis of Transcription: RNA Polymerase Backtracking and Its Reactivation. *Molecular Cell* **75**, 298-309.e4 (2019).
2. Mueller, A. U. & Darst, S. A. Structural basis for multi-subunit DNA-dependent RNA polymerase catalytic activity. *Molecular Cell* https://doi.org/10.1016/j.molcel.2026.03.033 (2026) doi:10.1016/j.molcel.2026.03.033.
3. Biertümpfel, C. *et al.* Structure and mechanism of human DNA polymerase η. *Nature* **465**, 1044–1048 (2010).
4. Li, Y., Korolev, S. & Waksman, G. Crystal structures of open and closed forms of binary and ternary complexes of the large fragment of Thermus aquaticus DNA polymerase I: structural basis for nucleotide incorporation. *The EMBO Journal* **17**, 7514–7525 (1998).
