# Supplementary material for: Structural basis of transcription of the hachimoji eight-letter alphabet by E. coli RNA polymerase
Source: Nat Commun. 2026 Sep 2;17:9060. doi: 10.1038/s41467-026-76668-0 (PMC13538358; doi:10.1038/s41467-026-76668-0)
Supplement: Supplementary file 3 [file 41467_2026_76668_MOESM3_ESM.pdf]

nature portfolio

Peer Review File

Structural Basis of Transcription of the Hachimoji Eight-Letter Alphabet by E. coli RNA Polymerase

Corresponding Author: Professor Dong Wang

This file contains all reviewer reports in order by version, followed by all author rebuttals in order by version.

Version 0:

Reviewer comments:

Reviewer #1

(Remarks to the Author)
This manuscript explores the structure/function of E. coli RNAP (EcoRNAP) and its incorporation of synthetic, non-canonical nucleotides into RNA transcripts. The authors show that EcoRNAP activity is compatible with two orthogonal unnatural base pairs (dZ:PTP and dP:Z*TP, using the nomenclature of the manuscript), and determine high-resolution cryo-EM structures of the relevant elongating complexes (using a 3'-deoxy RNA primer to trap the incoming unnatural substrates). Overall, the work is interesting but has pros and cons:

Pros:

• As mentioned, the authors show that EcoRNAP activity is compatible with the two orthongonal unnatural base pairs dZ:PTP and dP:Z*TP (Fig. 1B).

• The top panel of Fig. 1B (where the template base is Z) shows that, as expected, RNAP is able to incorporate PTP by forming an unnatural base pair with the Z. However, the assay also shows that GTP has a strong tendency to misincorporate as well. The authors design and assay a modified base (Z*) that essentially eliminates this problem (Fig. 2).

• The structural work, which is excellent, establishes that the unnatural base pairs situate themselves in the RNAP active site in Watson-Crick geometry, and can induce the closure of the RNAP active site into a catalytically competent state.

Cons:

• In Fig. 2B, the authors establish that the new base dZ* is much less effective than dZ in directing the misincorporation of GTP, and that in turn dG does not direct the misincorporation of Z*. However, the possibility exists that the use of Z* introduces some other problem in specificity with the other nucleotides. The authors should perform the same comprehensive test as shown in Fig. 1B for Z* (not just for dZ* directing the misincorporation of GTP or dG directing the misincorporation of Z*).

• The results in Fig. 1B show that template DNA dZ can direct the misincorporation of GTP, and this issue is addressed by the authors (Fig. 2). However, there are other anomalies in the transcription results that are simiarly robust but are ignored by the authors. For instance:

• dZ also directs the misincorporation of ATP.

• dP directs the incorporation of ZTP (as expected), but what is the explanation for the higher band? dP also appears to direct the misincorporation of CTP, and UTP (weakly).

• dS directs the inorporation of BTP (as expected), but also directs the misincorporation of ZTP, and ATP (weakly).

• dB directs the incorporation of STP as expected, but also directs the misincorporation of UTP.
These anomalies need to be discussed. Also, I think it would be informative if the results shown in Fig. 1B, Fig. 2, etc., were quantitated.

• The results shown in Fig. 1B suggest that the RNAPs incorporation rate of the unnatural substrates is extremely slow - in the assay shown in Fig. 1B, it would be good to add a 'positive control', a normal scaffold to compare the rate of incorporating a canonical nucleotide.

• The authors show that, at least in principle, EcoRNAP can incorporate the unnatural NTP substrates when the appropriate template base is present. However, to produce an RNA transcript that uses unnatural bases, the RNAP must also be able to translocate after incorporation, and then incoprorate additional NTPs after the unnatural base pair. this important issue is ignored here.

Reviewer #2

(Remarks to the Author)
Li et al. utilized cryo-EM to study the P:Z pair previously identified as potential synthetic nucleotides for an extended genetic alphabet and obtain the structures of E. coli RNAP incorporating these nucleotides into a transcript. The authors use in vitro transcription assays to identify the specificity of pairing among the new synthetic nucleotides and native nucleotides. The results show that the unnatural letters P, Z, S, and B can each direct the incorporation of its complement. In addition, misincorporation reactions are observed: dZ directs the incorporation of GTP and to a lesser extend ATP; dP directs the incorporation of CTP and UTP; dS directs the incorporation of ZTP and to a lesser extent ATP, and B directs the incorporation of UTP. Pairing between Z and G is well understood as arising from deprotonation of Z, which renders it complementary to G. This fidelity issue gets corrected partially by replacing Z with 2′-F-α-137 carboxamide-Z (Z*), which raises the pKa of the heterocycle. The authors show that Z* has reduced pairing with G, leading to greater fidelity to P. The authors employ cryo-EM to obtain structures for two constructs of RNAP bound with different template DNA and complementary NTP to mimic transcription elongation: one containing dZ the template strand and PTP (dZ: PTP) as the incoming NTP, and another containing dP in the template strand and Z*TP (dP: Z*PT). The dZ: PTP construct was analyzed into one closed conformation RNAP structure showing PTP positioned in the active site and paired to dZ in a Watson-Crick fashion. The dP: Z*PT construct was analyzed into 3 total structures: 2 open conformations and 1 closed conformation. In all three structures Z*PT was paired in a Watson-Crick fashion with dP, with only the positioning of the RNAP trigger loop changing across the structures. All of this is in concurrence to natural substrates during transcription. The authors establish a difference in the distribution of particles in open and closed configurations between the two constructs, attributing this to Z’s nitro group creating a nitro-dependent π-hole interaction that “stabilizes” bridge-helix bending. Overall, the manuscript describes advances in the understanding of the use of synthetic nucleotides in bacterial transcription so that they may be used to create an extended genetic alphabet. This may lead to development of non-standard amino acids to be encoded and incorporated into proteins during ribosomal translation as well as developing non-coding RNA aptamers with expanded functions or stability. Continued advancement of this system has potential to impact multiple areas of study. This manuscript shows substantial work to identify key insights into the P: Z pair during transcription.

1. The cryoEM analysis has revealed interesting structural features, i.e. the nitro-dependent π-hole interaction and the relative population of open to closed particles observed in the dP: Z*TP complex versus dZ: PTP complex. However, there is no attempt to assess the impact of these features on the function. Does the π-hole interaction manifest in the affinity of the polymerase for the template/primer? Because the complex has an apparently higher probability of adopting the closed complex, is the Km and rate of the complementary NTP incorporation altered? Does the π-hole interaction impact the translocation or rate of incorporation of the next nucleotide (i.e. the interaction would presumably need to be broken to incorporate the next nucleotide). In addition to presenting the structure, the authors include an entire section in the discussion on this interaction and call it “an unexpected recognition principle” in RNAP. The functional consequences need to be assessed.

2. Similarly, for the observation that dP: Z*TP produces particles in three conformations, how does this compare to natural dN: NTP particles? If dP: Z*TP produces a greater proportion of particles in the open conformation, how does this affect the kinetics of incorporation?

3. The claims that P: Z and S: B can be used as native-like bases for expanding a genetic alphabet, while foreseeable may be a bit too optimistic at this stage. Fig 6 shows a model for the use of this system that implies a system that is already ready for use in these ways. This manuscript, along with the previous paper, shows that these pairs can function similarly to native base pairs in the context of bacterial RNA production; however, the mispairing of nucleotides beyond that of Z: G addressed in this paper remains a concern for their true ability to act as an extended genetic alphabet at this stage. I think most readers would be interested in understanding what currently limits realization of Fig.6. Figure 1 shows some mispairings beyond the accounted for Z: G mispair. Readers would be interested in knowing what fidelity problems need to be resolved to have AEGIS functioning in an organism. Regarding noncoding RNA, the 2’-OH plays important roles in structured RNAs. Z* lacks the 2’-OH, so technically RNA is not being produced. This is not addressed in the manuscript and the 2’F group is not shown in Figure 2, even though it is a key difference between the two nucleotides. Further regarding Fig. 6 and associated text, the decoding center of the ribosome uses recognition of the 2’-OH in the minor groove of the codon-anticodon interaction to ensure accurate translation. Would a 2’-F in the message block or cause errors in translation?

4. The structural comparisons made are between dP: dZ*PT and dZ: PTP. While this covers P and Z in both the template and the NTP substrate, it results in missing a direct comparison between the Z and Z* in the structure. A Z*: PTP would

allow for analysis of how Z and Z* are positioned in the active site and how they may produce different specificities. Is the 2'-F atom required for Z* and does this preclude its use as a templating nucleotide?

5. Regarding Figure 1, the mispairing of Z and G is well accounted for and pairing between B (isoG) and U/T is relatively well understood as arising from an isoG tautomer that is complementary to U. Less seems to be known about how Z incorporates A, how P incorporates C and U, or how S incorporates Z. Although beyond the scope of the current paper, cryoEM structural analysis of these mismatched pairs in the active site could provide important insights into solving fidelity problems.

Minor:
1. Fig 1 and Fig 2: The gels show that for some NTPs there is extension beyond the intended +1. While this is not necessarily a problem, the authors do not provide any explanation for why this occurs.
2. The authors produce both TL-open and TL-closed structures for dP: Z*TP but only produce a TL-closed structure for dZ: PTP. However, they indicate they collected particles for TL-open conformation of dZ: PTP and generated a map. Why were these structures not produced.
3. Comparisons to RNAP with native nucleotides were performed and useful, however it may be insightful to include comparisons to the structure of RNAP with B:S pairing to provide insights into the differences seen between these synthetic nucleotide pairs.
4. Fig 5b: The coloring between the post translocated RNAP model (gray) and the legend (blue) is incorrect.
5. Fig. S4: The coloring and labeling conventions of the RNAP and the nucleotides in A should be consistent with those in B and C.
6. Table S1 and Fig S2: Only 25k particles were used for the map for TL-closed dP:Z*TP, which seems like very few for a resolution of 2.75 Å. However, the FSC curves look fine, so that resolution is believable.
7. Table S1: The model resolution should be reported at 0.5 FSC (Å), not 0.143 FSC like the map. Additionally, it is always positive to report the model-map FSC graphs in their entirety.

Reviewer #3

(Remarks to the Author)
I co-reviewed this manuscript with one of the reviewers who provided the listed reports. This is part of the Nature Communications initiative to facilitate training in peer review and to provide appropriate recognition for Early Career Researchers who co-review manuscripts.

Reviewer #4

(Remarks to the Author)
This is an excellent and crisply written piece that continues to deepen the explorations from the Benner lab on artificial base-pairing, bio-orthogonal, biomimicking systems with potentially massively important applications in biotechnology.

The point here is to a) expand on fine-tuning of the Z-P base pair by changing the nitro to the carboxamide and b) to demonstrate how both of these are incorporated using cryo-EM. While I am not thoroughly qualified to evaluate the quality of the cryo-EM data, the figures are convincingly presented and provide fine-structure rationalization of how E.coli RNA polymerase can use these modified ribonucleotides.

The work nicely pairs structural work with in-vitro kinetic analysis of matched and mismatched incorporation studies.

A few minor revisions are required to enhance clarity.

The authors use what seems to be a primer extension reaction where they've hybridized an RNA primer to the template strand and then there's another DNA oligo listed as being part of the non-template strand. The authors need to properly reference this assay and explain how this represents an elongating complex. While it's been a while that I've looked at in vitro models of transcription, this doesn't address open-complexes or initiation complexes. Minimally a discussion as to where this assay was developed and to what extent it captures other aspects of transcription e.g. initiation would be appreciated. If the authors wanted to go further, they should run transcription on bona-fide strong procaryotic promoters like LacUV5 or Gal4. One might ask here (or maybe in future work) whether these Hachimoji bases can be inserted at start-sites of transcription and/or present in the promoter regions.

While these studies certainly demonstrate significant fidelity w/respect to E.coli RNA polymerase, multiple elongations and longer-transcripts with either sequence analysis or RT-PCR using DNA bases would be good to demonstrate multiple incorporations and scoring fidelity. Admittedly this might be the basis of a different paper.

In terms of showing the effects of the nitro-modified base, if the nitro results in undesired deprotonation, why was it used in the first place. This is unclear; I had to draw resonance structures to appreciate how a carbocyclic nucleoside that would otherwise possess the same functionalization as C would normally adopt a DAD tautomer except for the fact that the nitro is there. Reminding the reader as to how the nitro functions to alter the resonance forms by showing the non-aromatic nitronate form would help the reader appreciate why the nitro was chosen in the first place and why the amide serves a similar purpose.

When a mismatch in favor of Z-G is found, this is interesting since I would have thought that A would be favored given the possibility of forming the DAD tautomer although Figure 2 nicely shows how the deprotonated Z pairs with G.

To the authors' credit they show this misincorporation however this may overestimate its misincorporation since in the absence of P, the enzyme is forced to introduce a G.

Other minor issues:

Figure 1: show the H-bonds, don't write NH2

Version 1:

Reviewer comments:

Reviewer #1

(Remarks to the Author)
The authors have done an excellent job addressing all of the reviewer concerns...

Reviewer #2

(Remarks to the Author)
The authors have done a thorough job of addressing the comments. A few minor issues are as follows:
1. Line 181-182: Repeat "for dG template" (Similarly, for dG template, we also observed multiple bands of ZTP misincorporation for the dG template)
2. Line 191: Did you mean "substituent"
3. Fig. S7 is mentioned after Fig. S8 and S9
4. Fig. S8 why do they compare dZ:PTP to dG:CTP rather than dC:GTP (unless there is not a structure of that)
5. Line 453: Remove line
6. A statement clearly indicating that the Z:P pair needs further optimization. I think the paper succeeds at showing the viability of pKa engineering like that done here, so indicating that this is the first step of further improvement directly would be good.

Reviewer #3

(Remarks to the Author)
I co-reviewed this manuscript with one of the reviewers who provided the listed reports. This is part of the Nature Communications initiative to facilitate training in peer review and to provide appropriate recognition for Early Career Researchers who co-review manuscripts.

Reviewer #4

(Remarks to the Author)
I have had a chance to review the authors' response to reviewer #3 (me); their responses have been convincing, polite and thorough. I have read the revised publications with attention to the highlights and believe that this work can be published without further ado.

Open Access This Peer Review File is licensed under a Creative Commons Attribution 4.0 International License, which permits use, sharing, adaptation, distribution and reproduction in any medium or format, as long as you give appropriate credit to the original author(s) and the source, provide a link to the Creative Commons license, and indicate if changes were made.
In cases where reviewers are anonymous, credit should be given to 'Anonymous Referee' and the source.
The images or other third party material in this Peer Review File are included in the article's Creative Commons license, unless indicated otherwise in a credit line to the material. If material is not included in the article's Creative Commons license and your intended use is not permitted by statutory regulation or exceeds the permitted use, you will need to obtain permission directly from the copyright holder.
To view a copy of this license, visit https://creativecommons.org/licenses/by/4.0/

Point-by-point Response

Reviewer #1

This manuscript explores the structure/function of E. coli RNAP (EcoRNAP) and its incorporation of synthetic, non-canonical nucleotides into RNA transcripts. The authors show that EcoRNAP activity is compatible with two orthogonal unnatural base pairs (dZ:PTP and dP:Z*TP, using the nomenclature of the manuscript), and determine high-resolution cryo-EM structures of the relevant elongating complexes (using a 3'-deoxy RNA primer to trap the incoming unnatural substrates). Overall, the work is interesting but has pros and cons:

Pros:

• As mentioned, the authors show that EcoRNAP activity is compatible with the two orthongonal unnatural base pairs dZ:PTP and dP:Z*TP (Fig. 1B).

• The top panel of Fig. 1B (where the template base is Z) shows that, as expected, RNAP is able to incorporate PTP by forming an unnatural base pair with the Z. However, the assay also shows that GTP has a strong tendency to misincorporate as well. The authors design and assay a modified base (Z*) that essentially eliminates this problem (Fig. 2).

• The structural work, which is excellent, establishes that the unnatural base pairs situate themselves in the RNAP active site in Watson-Crick geometry, and can induce the closure of the RNAP active site into a catalytically competent state.

We thank the reviewer for positive feedback on our work.

Cons:

1. In Fig. 2B, the authors establish that the new base dZ* is much less effective than dZ in directing the misincorporation of GTP, and that in turn dG does not direct the misincorporation of Z*. However, the possibility exists that the use of Z* introduces some other problem in specificity with the other nucleotides. The authors should perform the same comprehensive test as shown in Fig. 1B for Z* (not just for dZ* directing the misincorporation of GTP or dG directing the misincorporation of Z*).

We thank the reviewer for emphasizing the importance of comprehensively evaluating the specificity of dZ*. We performed a new transcription assay with a site-specific dZ* or dZ template strand and examined nucleotide incorporation against all four natural NTPs (ATP, GTP, CTP, and UTP) and four unnatural substrates (PTP, ZTP, BTP, and STP) (Fig. R1). These new results have been incorporated into the revised manuscript (Fig. 2B and Fig. S4). Among all eight substrates we tested, PTP is the most efficient substrate to be incorporated against dZ and dZ* templates. Consistent with our original report, we observed a substantial reduction of GTP misincorporation against dZ* template in comparison with dZ template, confirming that the modification improves selectivity against GTP. At the same time, we found that both dZ and dZ* exhibit comparable minor misincorporation with ATP, which is much less than GTP misincorporation. No other detectable misincorporation was observed for other NTP substrates. Taken together, these results strengthen our original conclusion that dZ* significantly improves specificity by reducing GTP misincorporation. We have revised our manuscript to include these new results.

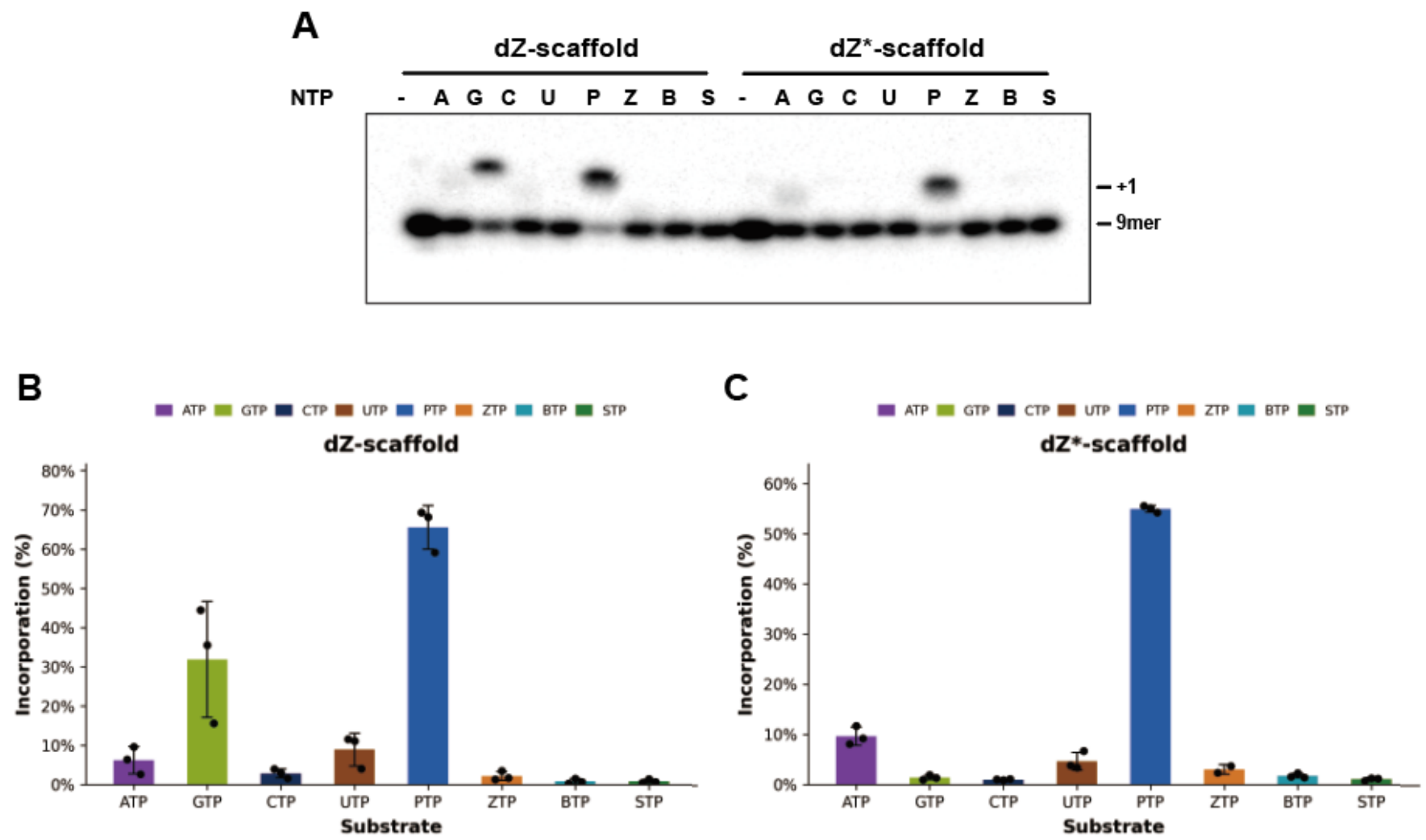

Fig. R1 | Single-nucleotide incorporation assay comparing the specificity profiles of dZ and dZ* scaffolds.
A: Single-nucleotide incorporation assay comparing scaffolds containing dZ or dZ* at the template i+1 position. *E. coli* RNAP elongation complexes were assembled on each scaffold and treated with 1 µM of the indicated NTP substrate, including all four natural NTPs (ATP, GTP, CTP, and UTP) and all four unnatural NTPs (PTP, ZTP, BTP, and STP). Reactions were sampled at 10 s, resolved by 12% denaturing urea-PAGE, and visualized by autoradiography of p^{32}-labeled RNA. All assays were performed in three independent replicates. B and C: Incorporation rates were quantified from Panel A and displayed as bar plots for each template scaffold (dP, dZ, dS, and dB). Band intensities were measured at the 10 s time point to calculate incorporation rate (%). The eight NTP substrates are displayed along the x-axis and colored as indicated in the legend. Data are presented as mean ± SD from three independent experiments (n = 3).

2. The results in Fig. 1B show that template DNA dZ can direct the misincorporation of GTP, and this issue is addressed by the authors (Fig. 2). However, there are other anomalies in the transcription results that are similarly robust but are ignored by the authors. For instance:

- dZ also directs the misincorporation of ATP.
- dP directs the incorporation of ZTP (as expected), but what is the explanation for the higher band? dP also appears to direct the misincorporation of CTP, and UTP (weakly).
- dS directs the incorporation of BTP (as expected), but also directs the misincorporation of ZTP, and ATP (weakly).
- dB directs the incorporation of STP as expected, but also directs the misincorporation of UTP. These anomalies need to be discussed. Also, I think it would be informative if the results shown in Fig. 1B, Fig.2, etc., were quantitated.
- The results shown in Fig. 1B suggest that the RNAPs incorporation rate of the unnatural substrates is extremely slow - in the assay shown in Fig. 1B, it would be good to add a 'positive control', a normal scaffold to compare the rate of incorporating a canonical nucleotide.

We thank the reviewer for the insightful comments. We focused on GTP for dZ template in our original manuscript, because it is the major misincorporation. We have reported major misincorporation on dS and dB templates in our previous work[1]. We have now included a description of other minor misincorporation in our revised manuscript.

Following the reviewer’s suggestion, we also quantified the extension band intensities from Fig. 1B and present the results as bar graphs (Fig. R2). Substrate discrimination between cognate and mismatch substrates is most pronounced at the early time point, indicating misincorporation is very slow in comparison with cognate substrate incorporation. Please note that in these single-nucleotide transcription assays, we “force” the misincorporation of single substrate in the absence of cognate substrate competition (polymerases have evolved to extend if they can possibly do so; they would rather misincorporate than to do nothing). Thus, the misincorporation frequencies observed here likely overestimate the actual error rates when all NTPs (including the cognate substrate) compete simultaneously (also acknowledged by Reviewer 4). The cognate substrates would kinetically outcompete rare and slow mismatches. We have revised the manuscript to include this discussion.

The quantitative analysis also reveals dS:ZTP misincorporation (Fig. R2D), which is consistent with the reviewer's concern. To assess whether the Z* modification could suppress this mispairing, we assembled elongation complexes on the dS scaffold and compared single-nucleotide incorporation of BTP, ZTP, and Z*TP under identical conditions (Fig. R2E). Z*TP substantially reduced misincorporation relative to ZTP on the dS scaffold, demonstrating that the carboxamide substitution improves substrate selectivity. These data are included in the revised manuscript as Fig. S4C.

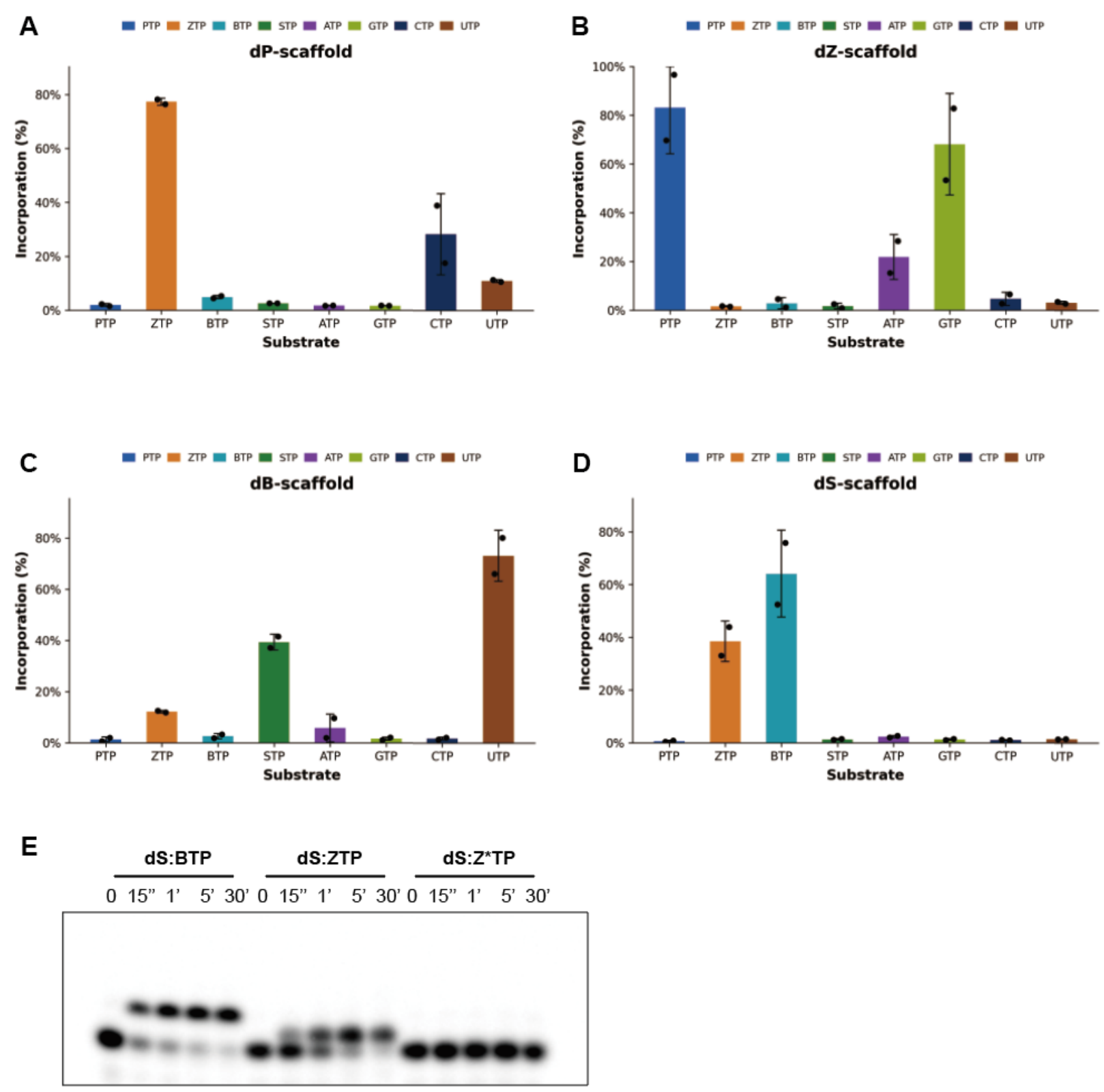

Fig. R2 | Incorporation efficiency of all eight Hachimoji NTP substrates across four UBP template scaffolds.
A–D: Incorporation rates were quantified from single-nucleotide transcription assays (Fig. 1B) and displayed as bar plots for each template scaffold (dP, dZ, dS, and dB). Band intensities were measured at the 15 s time point to calculate incorporation rate (%). The eight NTP substrates are displayed along the x-axis and colored as indicated in the legend. The 15 s time point was selected because substrate discrimination is most pronounced at early times, before misincorporation products have accumulated to significant levels. Data are presented as mean ± SD from two independent experiments (n = 2).

E: Single-nucleotide incorporation assay comparing BTP, ZTP, and Z*TP on the dS scaffold at the template i+1 position. *E. coli* RNAP elongation complexes were assembled on the dS scaffold and treated with 20 µM of each indicated substrate. Reactions were sampled at 15 s, 1 min, 5 min, and 30 min, resolved by 12% denaturing urea-PAGE, and visualized by autoradiography of p^{32}-labeled RNA. All assays were performed in two independent replicates.

To address the reviewer's concern that UBP incorporation rates might be extremely slow relative to natural nucleotides, we directly compare the incorporation rates for unnatural and natural base pairs. Specifically, we measured k_{obs} for dZ:PTP, dP:ZTP, and the natural dG:CTP pair under identical single-turnover conditions at 0.1 µM substrate (Fig. R3). The two UBP scaffolds represent the two orientations of the P:Z base pair, in which the template and substrate identities are switched. The k_{obs} values of PTP and ZTP incorporation are 0.09 s^{-1} and 0.10 s^{-1}, respectively. The natural dG:CTP control yielded a k_{obs} of 0.19 s^{-1} under the same conditions, approximately two folds higher than the UBP pair (Fig. R3). These data suggested that the UBP incorporation rates are comparable to canonical nucleotide incorporation under the same condition. We included this in the revised manuscript as Fig. S2.

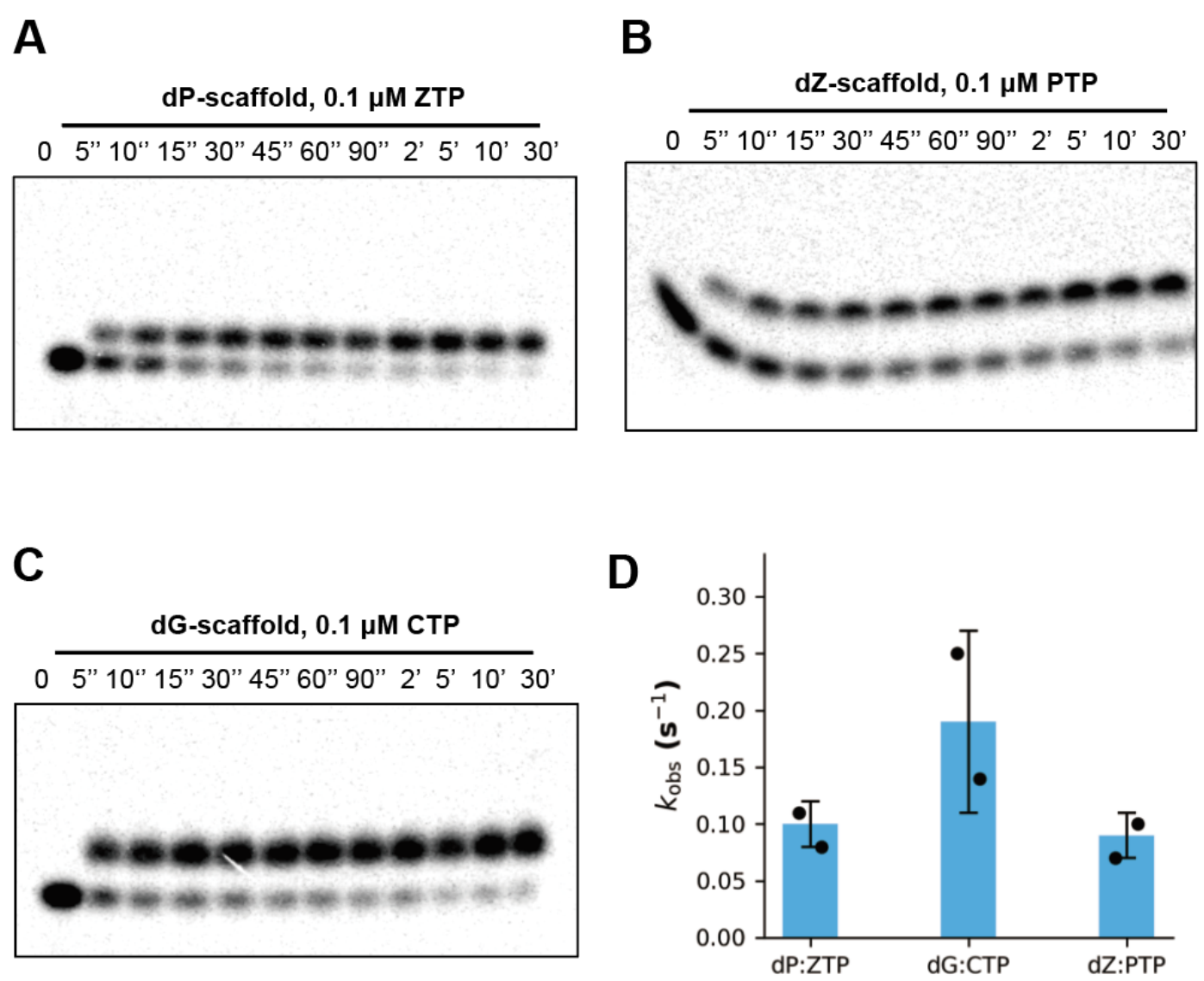

Fig. R3 | Single-nucleotide incorporation kinetics for dP, dZ, and dG scaffolds.
A–C: Single-nucleotide incorporation assay measuring observed rate constants (k_{obs}) for three scaffold-substrate combinations. *E. coli* RNAP elongation complexes were assembled on scaffolds containing dP, dZ, or dG at the template i+1 position and treated with 0.1 µM of the indicated NTP substrate (ZTP, PTP, or CTP). Reactions were sampled at 0 s, 5 s, 10 s, 15 s, 30 s, 45 s, 60 s, 90 s, 2 min, 5 min, 10 min, and 30 min, resolved by 12% denaturing urea-PAGE, and visualized by autoradiography of p^{32}-labeled RNA. All assays were performed in two independent replicates.
D: Bar plot of k_{obs} (s^{-1}) was derived by single-exponential fitting (Panel A–C). Data are presented as mean ± SD from two independent experiments (n = 2).

3. The authors show that, at least in principle, EcoRNAP can incorporate the unnatural NTP substrates when the appropriate template base is present. However, to produce an RNA transcript that uses unnatural bases, the RNAP must also be able to translocate after incorporation, and then incorporate additional NTPs after the unnatural base pair. This important issue is ignored here.

We thank the reviewer for this important consideration. The ability of RNAP to continue RNA synthesis beyond incorporated UBP is essential for evaluating the utility of the P:Z system in RNA production. To address this, we performed NTP-chase transcription assays using the scaffolds containing four different UBP configurations (dZ:PTP, dP:ZTP, dZ*:PTP, and dP:Z*TP), respectively. RNAP was first allowed to incorporate the cognate UBP substrate in a single-nucleotide extension reaction (5 min), and then chased with 1 mM of natural rNTP mixture (ATP, GTP, CTP, and UTP) for an additional 5 min. For all four UBP scaffold conditions tested (S3 lanes: chased with rNTP), we observed robust RNA transcript extension products beyond the UBP-containing i+1 position (Fig. R4, higher extension bands in S3 lanes). In addition, we also observed no strong pausing band at i+2 position, indicating that RNAP can rapidly translocate after incorporating a non-canonical base efficiently and remains catalytically competent for continued transcription elongation. Finally, we also chased with next cognate nucleotide addition (Fig. R6) and found that rapid UTP incorporation for both dP and dZ template (Fig. R6). Taken together, these results establish that UBP incorporation is not a terminating event and that the elongation complex can productively re-enter the nucleotide addition cycle following P:Z or P:Z* pairing. We have included this data as a new figure in the revised manuscript (Fig. S5A) and discussed their significance in the context of UBP-containing RNA synthesis.

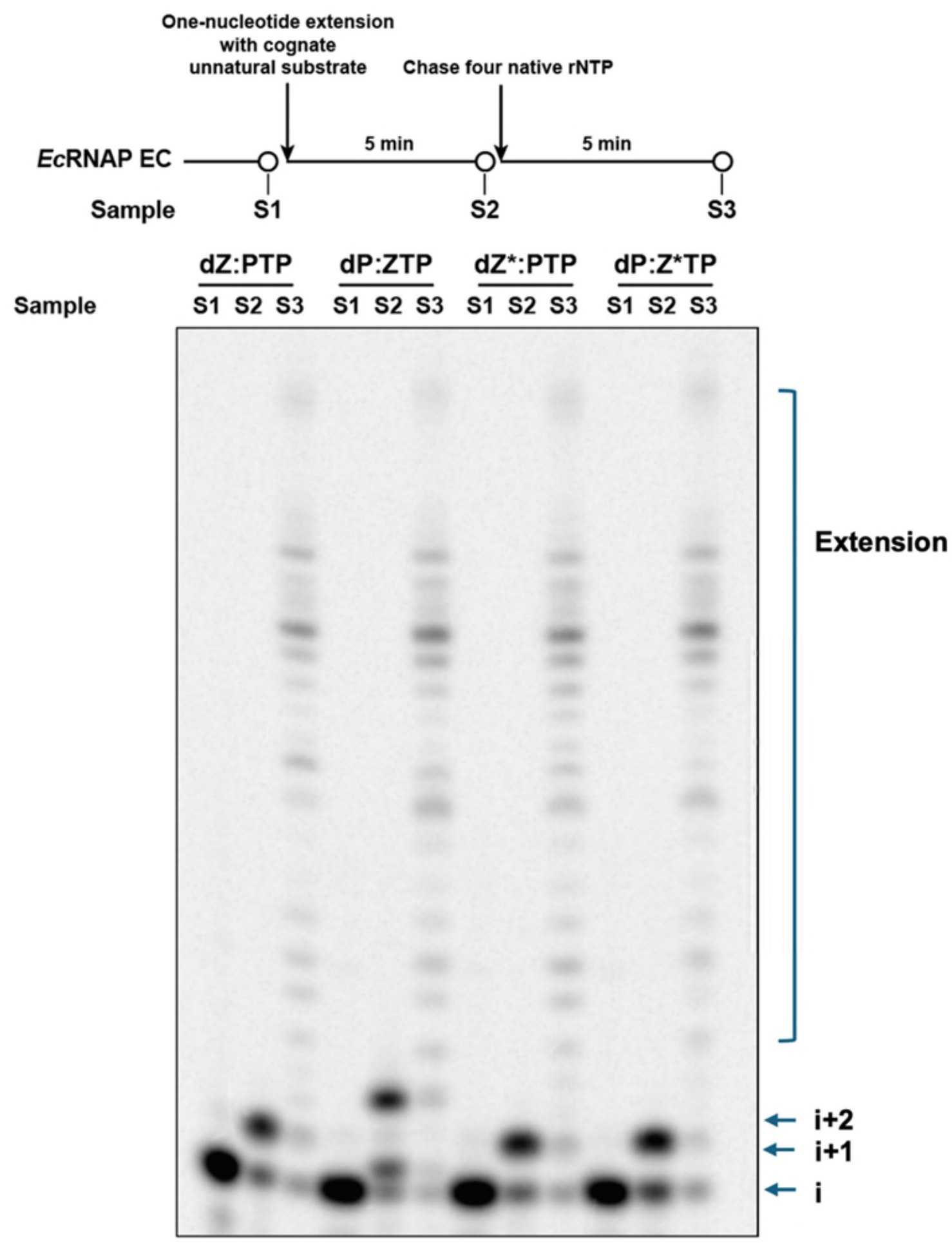

Fig. R4 | NTP-chase assay demonstrating RNAP translocation and continued elongation after UBP incorporation. *E. coli* RNAP elongation complexes were assembled on scaffolds containing dZ, dP, or dZ* at the template i+1 position. Four scaffold-substrate combinations were tested, including dZ:PTP,

dP:ZTP, dZ*:PTP, and dP:Z*TP. For each configuration, the cognate UBP substrate was first added and incubated for 5 min to allow single-nucleotide extension at the i+1 position. All four natural rNTPs (ATP, GTP, CTP, and UTP) were then added as a chase and incubated for a further 5 min. Three samples were collected for each scaffold-substrate pair: the initial elongation complex prior to substrate addition (S1), the complex after UBP incorporation (S2), and the complex after rNTP chase (S3). Samples were resolved by 12% denaturing urea-PAGE and visualized by autoradiography of p^{32}-labeled RNA. The upper panel shows a schematic of the experimental procedure, and the lower panel shows representative gel images for all four scaffold-substrate configurations. (Note: upper pausing bands are well-established, natural sequence-specific pausing bands that are irrelevant to Z/Z*:P pair). All assays were performed in two independent replicates.

Reviewer #2

Li et al. utilized cryo-EM to study the P:Z pair previously identified as potential synthetic nucleotides for an extended genetic alphabet and obtain the structures of E. coli RNAP incorporating these nucleotides into a transcript. The authors use in vitro transcription assays to identify the specificity of pairing among the new synthetic nucleotides and native nucleotides. The results show that the unnatural letters P, Z, S, and B can each direct the incorporation of its complement. In addition, misincorporation reactions are observed: dZ directs the incorporation of GTP and to a lesser extend ATP; dP directs the incorporation of CTP and UTP; dS directs the incorporation of ZTP and to a lesser extent ATP, and B directs the incorporation of UTP. Pairing between Z and G is well understood as arising from deprotonation of Z, which renders it complementary to G. This fidelity issue gets corrected partially by replacing Z with 2′-F-α-137 carboxamide-Z (Z*), which raises the pKa of the heterocycle. The authors show that Z* has reduced pairing with G, leading to greater fidelity to P. The authors employ cryo-EM to obtain structures for two constructs of RNAP bound with different template DNA and complementary NTP to mimic transcription elongation: one containing dZ the template strand and PTP (dZ: PTP) as the incoming NTP, and another containing dP in the template strand and Z*TP (dP: Z*PT). The dZ: PTP construct was analyzed into one closed conformation RNAP structure showing PTP positioned in the active site and paired to dZ in a Watson-Crick fashion. The dP: Z*PT construct was analyzed into 3 total structures: 2 open conformations and 1 closed conformation. In all three structures Z*PT was paired in a Watson-Crick fashion with dP, with only the positioning of the RNAP trigger loop changing across the structures. All of this is in concurrence to natural substrates during transcription. The authors establish a difference in the distribution of particles in open and closed configurations between the two constructs, attributing this to Z's nitro group creating a nitro-dependent π-hole interaction that "stabilizes" bridge-helix bending. Overall, the manuscript describes advances in the understanding of the use of synthetic nucleotides in bacterial transcription so that they may be used to create an extended genetic alphabet. This may lead to development of non-standard amino acids to be encoded and incorporated into proteins during ribosomal translation as well as developing non-coding RNA aptamers with expanded functions or stability. Continued advancement of this system has potential to impact multiple areas of study. This manuscript shows substantial work to identify key insights into the P: Z pair during transcription.

We thank the reviewer for the comprehensive summary and positive comments on our work.

1. The cryoEM analysis has revealed interesting structural features, i.e. the nitro-dependent π-hole interaction and the relative population of open to closed particles observed in the dP: Z*TP complex versus dZ: PTP complex. However, there is no attempt to assess the impact of these features on the function. Does the π-hole interaction manifest in the affinity of the polymerase for the template/primer? Because the complex has an apparently higher probability of adopting the closed complex, is the Km and rate of the complementary NTP incorporation altered? Does the π-hole interaction impact the translocation or rate of incorporation of the next nucleotide (i.e. the interaction would presumably need to be broken to incorporate the next nucleotide). In addition to presenting the structure, the authors include an entire section in the discussion on this interaction and call it "an unexpected recognition principle" in RNAP. The functional consequences need to be assessed.

We thank the reviewer for these important questions. For the questions regarding whether the π-hole interaction manifests itself in the affinity of the polymerase residues and the template/primer, the short answer is "unlikely". Structural studies (including ours) revealed that the affinity of the polymerase and the template/primer rely on extensive interactions between the polymerase residues and the template strand and primer strand, which spans over the entire scaffold including both downstream and upstream. Therefore, the single π-hole interaction at +1 position is unlikely to change the overall affinity between the polymerase residues and the template/primer.

To directly assess the effect of the π-hole interaction on NTP incorporation kinetics, we measured k_{obs} for dZ:PTP and dZ*:PTP incorporation under identical single-turnover conditions at 0.1 µM PTP (Fig. R5). Because dZ and dZ* differ only in their template base functional group (a nitro group in dZ versus a carboxamide in dZ*), this comparison directly reflects the kinetic contribution of the nitro-dependent π-hole interaction. The dZ scaffold yielded a k_{obs} of 0.09 s^{-1}, approximately 1.8-fold higher than the k_{obs} of 0.05 s^{-1} measured for the dZ* scaffold, indicating that the presence of the nitro group is associated with a measurable increase in PTP incorporation rate. One possible interpretation consistent with these data is that the π-hole interaction stabilizes the TL-closed state, shifting a greater fraction of elongation complexes into the catalytically competent conformation and thereby increasing the observed incorporation rate. These findings have been incorporated into the revised manuscript as Fig. S2.

To address the potential influence on subsequent nucleotide incorporation, we performed a UTP-chase experiment using four scaffold-substrate pairs: dZ:PTP, dP:ZTP, dZ*:PTP, and dP:Z*TP (Fig. R6). Among these, only the dZ:PTP configuration engages the nitro-dependent π-hole interaction through the dZ template base; the remaining three configurations lack this interaction and serve as controls. *Ec*RNAP EC was first loaded with the cognate UBP substrate and then supplemented with UTP at 15 s, 1 min, 5 min, and 30 min to monitor next-nucleotide addition kinetics following UBP loading. Comparing UTP incorporation kinetics across all three configurations revealed no significant difference, indicating that the π-hole interaction does not influence the rate of subsequent nucleotide addition. These results demonstrate that the elongation complex remains fully competent for continued RNA synthesis after UBP incorporation, regardless of whether the preceding template base engage a π-hole interaction. These results are included in the revised manuscript as Fig. S5B.

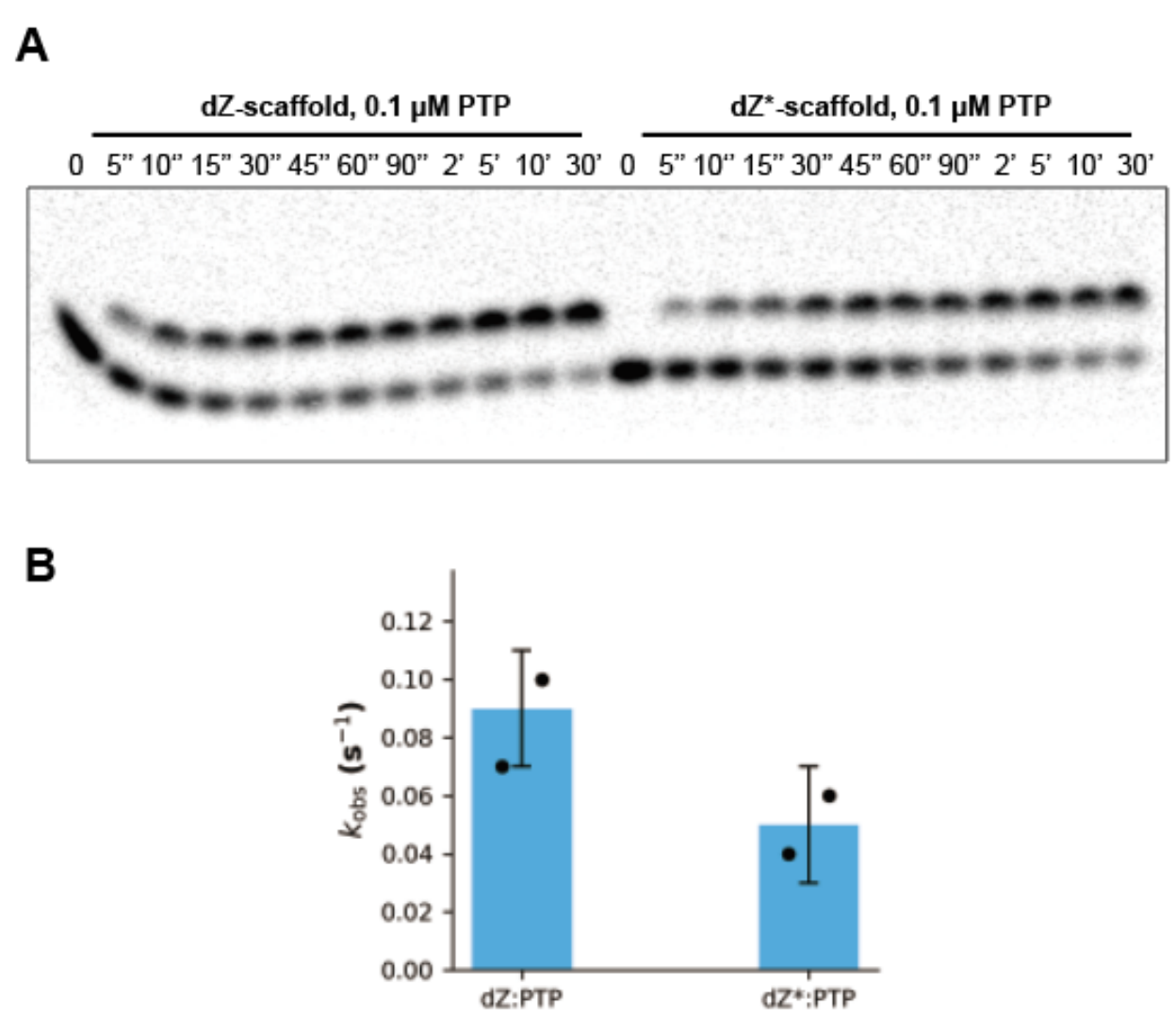

Fig. R5 | Effect of the nitro-dependent π-hole interaction on UBP incorporation kinetics.
A: Single-nucleotide incorporation assay measuring k_{obs} to compare dZ and dZ* as template scaffolds, both tested with 0.1 µM PTP. Because dZ and dZ* differ only in their template base functional group (a nitro group in dZ versus a carboxamide in dZ*), their k_{obs} comparison directly reflects the kinetic contribution of the nitro-dependent π-hole interaction. Elongation complexes were assembled on scaffolds containing dZ or dZ* at the template i+1 position and treated with 0.1 µM PTP. Reactions for both scaffolds were sampled at 5 s, 10 s, 15 s, 30 s, 45 s, 60 s, 90 s, 2 min, 5 min, 10 min, and 30 min. All assays were performed in two independent replicates.
B: Bar plot of k_{obs} (s^{-1}) was derived by single-exponential fitting (Panel A). Data are presented as mean ± SD from two independent experiments (n = 2).

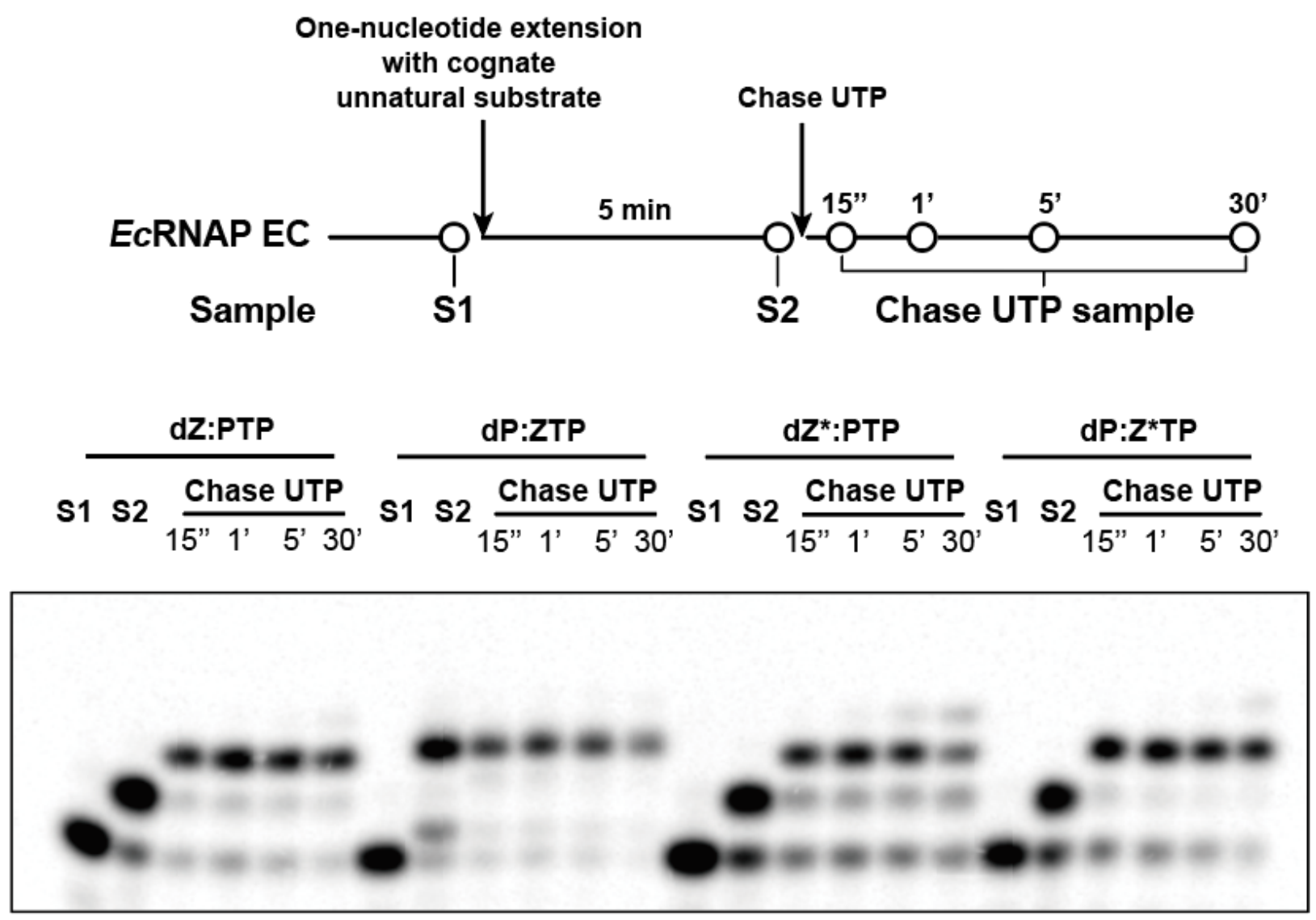

Fig. R6 | Effect of the nitro-dependent π-hole interaction on next-nucleotide (UTP) incorporation, assessed by UTP-chase assay. *E. coli* RNAP elongation complexes were assembled on four scaffold-substrate configurations (dZ:PTP, dP:ZTP, dZ*:PTP, and dP:Z*TP), each with the UBP positioned at the template i+1 site. The dZ:PTP configuration generates a π-hole interaction via the dZ nitro group; the dP:ZTP, dP:Z*TP and dZ*:PTP configurations use dP and dZ* as the template base, in which this interaction is absent, and serve as controls. For each configuration, the cognate UBP substrate was first added and incubated for 5 min to allow single-nucleotide extension. UTP was then added to a final concentration of 20 μM and reactions were sampled at 15 s, 1 min, 5 min, and 30 min. Six samples were collected per configuration: the initial elongation complex prior to substrate addition, the complex after UBP incorporation, and the complex at each of the four UTP-chase time points. Samples were resolved by 12% denaturing urea-PAGE and visualized by autoradiography of p^{32}-labeled RNA. All assays were performed in two independent replicates.

2. Similarly, for the observation that dP: Z*TP produces particles in three conformations, how does this compare to natural dN: NTP particles? If dP: Z*TP produces a greater proportion of particles in the open conformation, how does this affect the kinetics of incorporation?

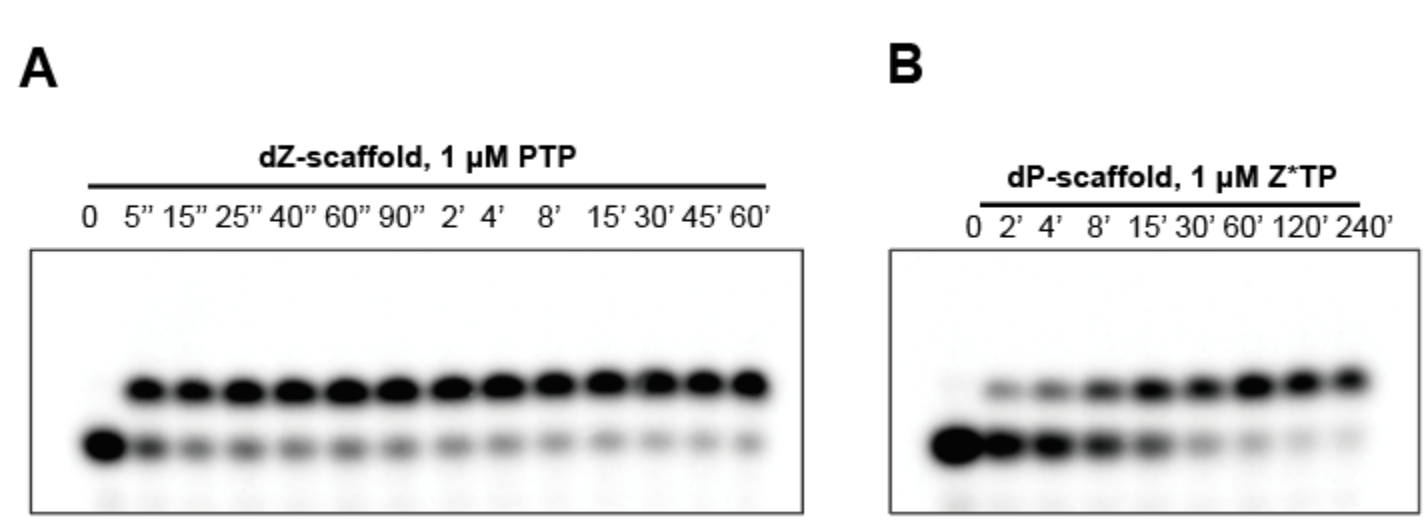

Fig. R7 | Single-nucleotide incorporation kinetics for dZ:PTP and dP:Z*TP. A–B: Single-nucleotide incorporation assay measuring k_{obs} for the dZ:PTP (A) and dP:Z*TP (B) scaffold-substrate pairs used in the cryo-EM elongation complexes reported in this study. *E. coli* RNAP elongation complexes were assembled on scaffolds containing dZ or dP at the template i+1 position and treated with 1 μM PTP or Z*TP, respectively. Given their differing incorporation rates, distinct time-course protocols were applied: reactions with dZ:PTP were sampled at 0 s, 5 s, 15 s, 25 s, 40 s, 60 s, 90 s, 2 min, 4 min, 8 min, 15 min, 30 min, 45 min, and 60 min, while reactions with dP:Z*TP were sampled at 0 s, 2 min, 4 min, 8 min, 15 min, 30 min, 120 min, and 240 min. Samples were collected and resolved by 12% denaturing urea-PAGE and visualized by autoradiography of p^{32}-labeled RNA. All assays were performed in two independent replicates.

We thank the reviewer for this insightful question. A recent cryo-EM study (Dhingra et al., bioRxiv 2026)[2] assembled a *Mycobacterium tuberculosis* RNAP elongation complex using the same 3'-deoxy RNA scaffold strategy employed in our cryo-EM experiments, with natural GTP loaded at the i+1 site. This system shares both the scaffold design and the bacterial RNAP class of our study, making it a well-matched structural benchmark. In that dataset, 70% of particles adopted the TL-closed conformation, closely comparable to the 76.8% observed in our dZ:PTP dataset. In contrast, the dP:Z*TP dataset showed a substantially lower TL-closed fraction of 19.4%, indicating that Z*TP is markedly less effective than PTP or a natural cognate NTP at stabilizing the TL-closed conformation.

To assess whether these conformational distributions are consistent with incorporation kinetics, we measured k_{obs} by single-nucleotide incorporation assay at 1 μM cognate NTP substrate (Fig. R7). The dZ:PTP scaffold yielded a k_{obs} of 0.28 s^{-1}, approximately 215-fold higher than the 0.0013 s^{-1} measured for dP:Z*TP. This large kinetic difference is consistent with the substantially higher TL-closed fraction in the dZ:PTP dataset relative to dP:Z*TP (76.8% versus 19.4%), supporting the interpretation that a higher proportion of TL-closed particles reflects more productive substrate positioning and active site closure. These data are included in the revised manuscript as Fig. S2.

3. The claims that P: Z and S: B can be used as native-like bases for expanding a genetic alphabet, while foreseeable may be a bit too optimistic at this stage. Fig 6 shows a model for the use of this system that implies a system that is already ready for use in these ways. This manuscript, along with the previous paper, shows that these pairs can function similarly to native base pairs in the context of bacterial RNA production; however, the mispairing of nucleotides beyond that of Z: G addressed in this paper remains a concern for their true ability to act as an extended genetic alphabet at this stage. I think most readers would be interested in understanding what currently limits realization of Fig.6. Figure 1 shows some mispairings beyond the accounted for Z: G mispair. Readers would be interested in knowing what fidelity problems need to be resolved to have AEGIS functioning in an organism. Regarding noncoding RNA, the 2'-OH plays important roles in structured RNAs. Z* lacks the 2'-OH, so technically RNA is not being produced. This is not addressed in the manuscript and the 2'F group is not shown in Figure 2, even though it is a key difference between the two nucleotides. Further regarding Fig. 6 and associated text, the decoding center of the ribosome uses recognition of the 2'-OH in the minor groove of the codon-anticodon interaction to ensure accurate translation. Would a 2'-F in the message block or cause errors in translation?

We thank the reviewer for careful reading and critical comments. We fully agree with the reviewer that there still is a lot of work needed to be optimized to use the AEGIS system for expanded genetic alphabet. Fig. 6 is rather an outlook for the system in future. We have revised the figure title and figure legend and included a paragraph for discussion of future outlook and limitations, including the fidelity limitations (as the reviewer correctly pointed out).

The question of additional misincorporation events observed in Figs. 1B and 2, including dZ:ATP, dP:CTP/UTP, and dS:ZTP, overlaps substantially with the concerns raised by Reviewer 1 (Reviewer 1-Question 2). In that response, we present a quantitative analysis of band intensities displayed as bar plots for each scaffold (Fig. R2). We refer the reviewer to our response for a full quantitative treatment. Briefly, polymerases have evolved to extend if possible; they would rather misincorporate than to do nothing. Thus, misincorporation in the absence of cognate nucleotide overestimates the misincorporation rates. Please note that an important intrinsic limitation of the single-nucleotide transcription assay (Figs. 1B and 2) is that each reaction contains only one NTP substrate in the absence of competing cognate substrates, which systematically overestimates error frequencies relative to any in vivo condition where all NTPs compete simultaneously for the active site. The misincorporation rate is expected to be substantially lower in the presence of cognate NTP, as fast cognate NTP incorporation will kinetically outcompete slow misincorporation.

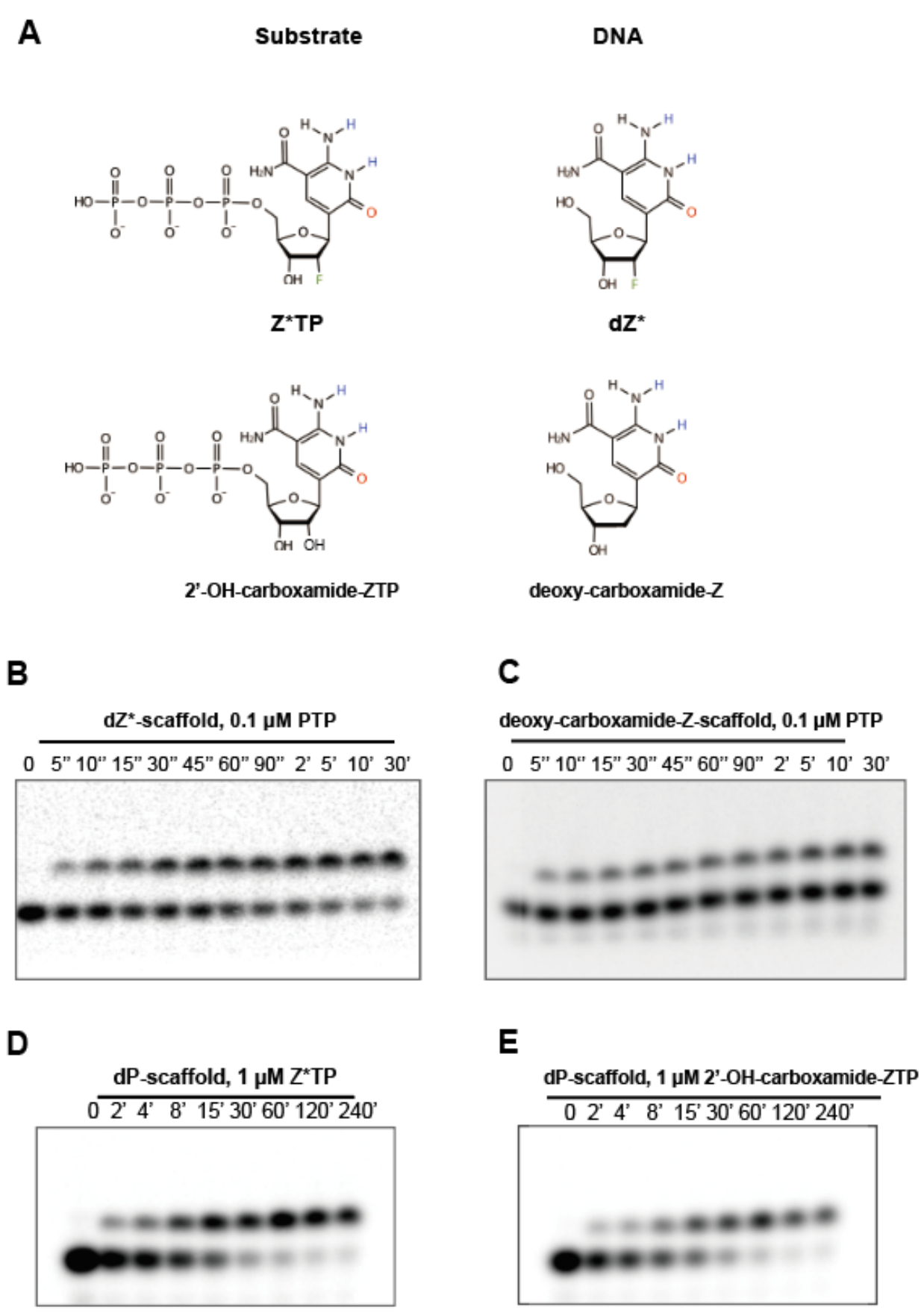

Fig. R8 | Kinetic comparison of carboxamide-Z analogs bearing 2'-F or 2'-OH modifications as template bases and NTP substrates. A: Chemical structures of carboxamide-Z analogs with 2'-F (Z*) or 2'-OH substitutions, shown as template DNA nucleosides and as incoming NTP substrates.
B–C: Single-nucleotide incorporation assay measuring k_{obs} for dZ*:PTP (B) and deoxy-carboxamide-Z:PTP (C) scaffold-substrate pairs. *E. coli* RNAP elongation complexes were assembled on scaffolds containing dZ* or deoxy-carboxamide-Z at the template i+1 position and treated with 0.1 μM PTP. Reactions were sampled at 0 s, 5 s, 10 s, 15 s, 30 s, 45 s, 60 s, 90 s, 2 min, 5 min, 10 min, and 30 min, resolved by 12% denaturing urea-PAGE, and visualized by autoradiography of p^{32}-labeled RNA.
D–E: Single-nucleotide incorporation assay measuring k_{obs} for Z*TP (D) and 2'-OH-carboxamide-ZTP (E) on the dP scaffold at the template i+1 position. Elongation complexes were treated with 1 μM of each

indicated substrate and reactions were sampled at 0 s, 2 min, 4 min, 8 min, 15 min, 30 min, 120 min, and 240 min, resolved by 12% denaturing urea-PAGE, and visualized by autoradiography of p^{32}-labeled RNA.

We thank the reviewer for noting that the 2'-F group of Z* was not shown in Fig. 2. We have added a new Fig. S3 to include it. As for the question on RNA synthesis, please note that for the dP template, we can also use ZTP (contains regular 2'-OH) (Fig 1B) or the recently developed carboxyamide-rZTP variant (contains 2'-OH) as cognate nucleotide substrates to support RNA synthesis opposite dP template (Fig. R8).

It is also worth noting now that many RNA polymerases are capable of using 2'-fluoro nucleosides as if they were ribonucleotides[3]. The presence of 2'-F in place of 2'-OH in Z*TP means that any RNA product containing Z* is not a conventional ribonucleotide, and the functional consequences of this substitution for downstream biological processes, including structured RNA folding and ribosomal decoding, represent important open questions for future studies. The related question of whether 2'-F in an mRNA codon would block or introduce errors in translation is similarly outside the scope of this work, which is focused exclusively on the transcription step catalyzed by *Ec*RNAP. The Benner group has conducted studies examining the behavior of AEGIS bases in translation contexts. Of course, we see this as an important concurrent/following step for the broader goal of deploying the Hachimoji alphabet in translation, both in vitro and living organisms.

4. The structural comparisons made are between dP: Z*TP and dZ: PTP. While this covers P and Z in both the template and the NTP substrate, it results in missing a direct comparison between the Z and Z* in the structure. A Z*: PTP would allow for analysis of how Z and Z* are positioned in the active site and how they may produce different specificities. Is the 2'-F atom required for Z* and does this preclude its use as a templating nucleotide?

The edge of nucleobase of Z and Z* that forms a Watson-Crick-like base pair with P remains the same, although Z* contains 2'-F in its ribose. The role of 2'-F of Z* is to confer epimerization stability: Kim et al. (ACS Chem Biol 2025)[4] demonstrated that the carboxamide-substituted Z analog without 2'-F undergoes rapid epimerization, and that introduction of 2'-F substantially stabilizes the β anomer by reducing ring oxygen basicity. We have clarified this point in the revised manuscript.

Our current structural datasets provide a comprehensive perspective of P:Z/Z* pairs in template and substrate: the dZ:PTP complex positions dZ in the template strand (we choose to use dZ rather than Z* as DNA template strand because the 2'-F ribose of Z* makes it more RNA-like than DNA-like), while the dP:Z*TP complex positions Z*TP as the incoming substrate, allowing us to characterize the Z* ribose environment at the active site. Indeed, as shown in our structures, P:Z and P:Z* form similar Watson-Crick-like base pair.

On the other hand, we should also point out that RNA polymerases can tolerate and bypass the natural ribonucleoside in its DNA template. Similarly, RNA polymerases can also tolerate and bypass 2'-F substituent ribonucleoside at the template strand, as shown in our biochemical assays.

5. Regarding Figure 1, the mispairing of Z and G is well accounted for and pairing between B (isoG) and U/T is relatively well understood as arising from an isoG tautomer that is complementary to U. Less seems to be known about how Z incorporates A, how P incorporates C and U, or how S incorporates Z. Although beyond the scope of the current paper, cryoEM structural analysis of these mismatched pairs in the active site could provide important insights into solving fidelity problems.

We agree that the mechanistic origins of misincorporation events observed in our gel assays remain incompletely understood. However, and again, we would like to point out that current primer extension

experiments (without the complementary nucleoside triphosphate) would force the polymerase to misincorporate. Therefore, the observed misincorporation rates are likely overestimated. We expect misincorporation rates would be substantially lower in the presence of cognate substrate competition. Elucidating the active-site geometry underlying each mismatch event will require dedicated structural studies, and we fully agree that such work could provide important insights for future fidelity engineering efforts. We have acknowledged this as a high-priority direction for future work in the revised Discussion.

Minor:

1. Fig 1 and Fig 2: The gels show that for some NTPs there is extension beyond the intended +1. While this is not necessarily a problem, the authors do not provide any explanation for why this occurs.

We thank the reviewer for this careful observation. The extension products beyond i+1 arise from misincorporation of the same NTP at the downstream template positions (i+2 and i+3). In the single-nucleotide transcription assay, each reaction contains only one NTP species, and if that NTP has misincorporation activity against the template bases at i+2 or i+3 positions in the scaffold sequence, RNAP can continue extending after the initial i+1 event. For example, in the dP-template reactions, ZTP exhibits detectable misincorporation opposite both A and G, and the downstream template positions happen to present dA and dG; consequently, ZTP can generate i+2 and i+3 extension products in those lanes. The same logic applies to other NTPs that show misincorporation against the downstream template sequence. These bands therefore reflect the same misincorporation chemistry discussed in our response to Review 1-Question 2, here manifesting as multi-nucleotide extension. We have added a brief explanation in the relevant figure legend in the revised manuscript.

2. The authors produce both TL-open and TL-closed structures for dP: Z*TP but only produce a TL-closed structure for dZ: PTP. However, they indicate they collected particles for TL-open conformation of dZ: PTP and generated a map. Why were these structures not produced.

We thank the reviewer for this question. For the dZ:PTP dataset, 3D classification focused on the SI3–TL region revealed that only 23.2% of particles adopted the TL-open conformation. While a map for this class was generated, the limited number and poor quality of these particles did not permit reliable atomic model building. We therefore focused our analysis on the higher-quality TL-closed reconstruction (76.8% of particles, 2.42 Å), which represents the dominant and functionally most relevant pre-catalytic state. We have clarified this decision in the revised Methods section.

3. Comparisons to RNAP with native nucleotides were performed and useful, however it may be insightful to include comparisons to the structure of RNAP with B:S pairing to provide insights into the differences seen between these synthetic nucleotide pairs.

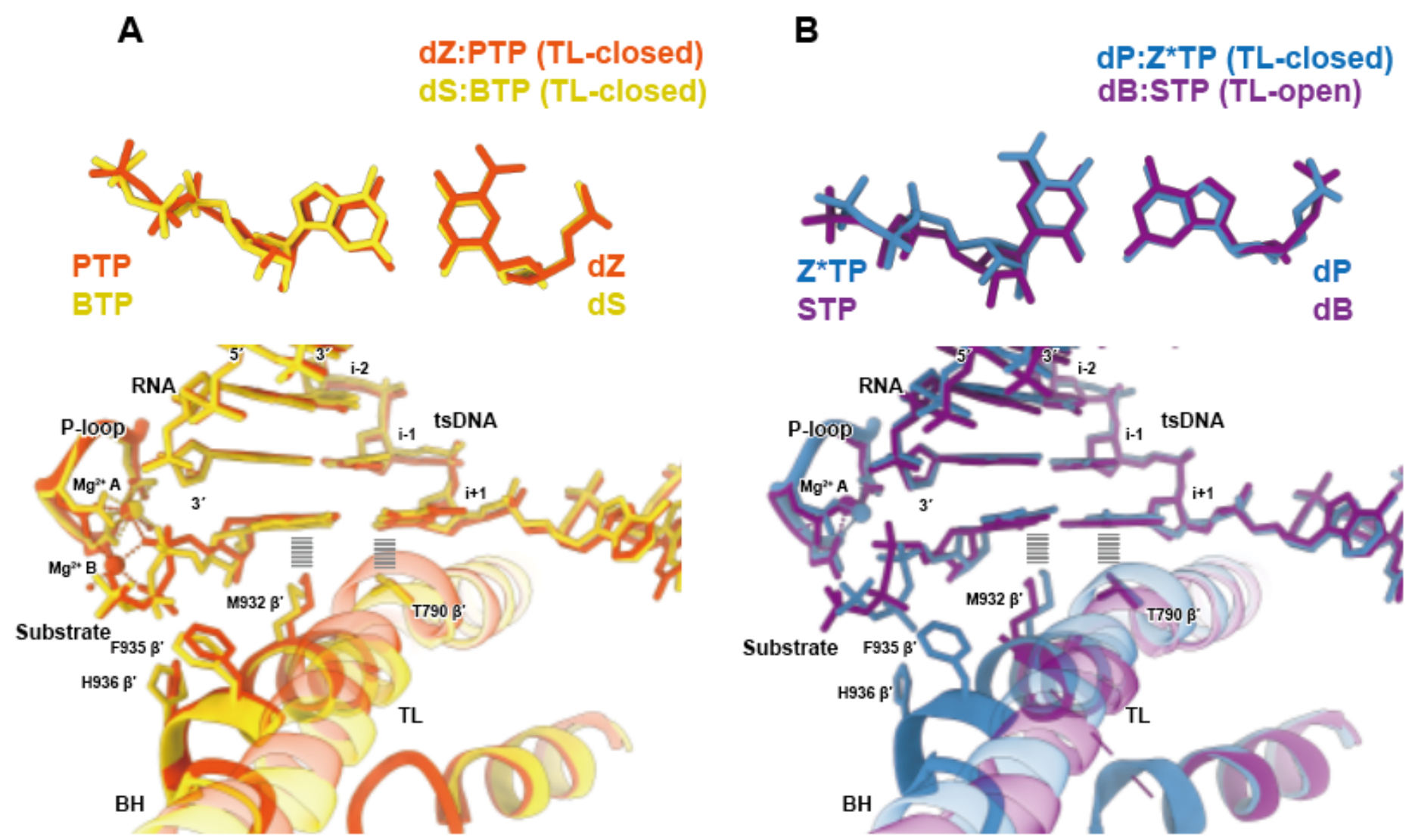

Fig. R9 | Structural comparison between *Ec*RNAP EC containing P:Z and B:S focusing on the base pairing and active site. A: Comparison between the dZ:PTP EC (orange red) and the dS:BTP EC (yellow; PDB: 8SY5). The upper panel focuses on the base pairing geometry; the lower panel focuses on the active site architecture. Key structural elements, including RNA, tsDNA, P-loop, trigger loop (TL), catalytic magnesium ions, and BH, are shown to illustrate the high degree of structural conservation between these two UBP-containing complexes. **B:** Comparison between the dP:Z*TP EC (steel blue) and the dB:STP EC (purple; PDB: 8SY7). The upper and lower panels follow the same format as panel A.

We thank the reviewer for this suggestion. We have added a brief structural comparison to the previously published B:S elongation complex[1], focusing on base pairing geometry and active site architecture (Fig. R9). To enable a size-matched comparison, structures were paired by substrate nucleobase size: dZ:PTP (purine substrate PTP) was compared with dS:BTP (purine substrate BTP; PDB: 8SY5), and dP:Z*TP (pyrimidine substrate Z*TP) was compared with dB:STP (pyrimidine substrate STP; PDB: 8SY7).

For base pairing geometry, both the P:Z and B:S pairs adopt canonical Watson-Crick geometries within the *E. coli* RNAP active site, with the nucleobases occupying nearly identical positions across all structures (Fig. R9). For active site architecture, the dZ:PTP complex adopts a configuration closely similar to the dS:BTP complex: both capture the TL-closed conformation, and key active site residues, including TL residues M932, F935, and H936 and BH residue T790, adopt similar positions and geometries. An analogous conservation is observed between dP:Z*TP and dB:STP, with nearly overlapping base pairing positions and active site geometry. Notably, the published dB:STP structure captures a substrate-loaded TL-open conformation. Although the TL state differs from the TL-closed configuration in our dP:Z*TP dataset, key active site residues M932 and T790 nonetheless adopt similar positions and conformations across the two structures. Together, these comparisons demonstrate that the P:Z and B:S unnatural base pairs are accommodated by *E. coli* RNAP in a structurally highly similar manner, with both pairs adopting canonical Watson-Crick geometry and engaging the same set of active site residues.

4. Fig 5b: The coloring between the post translocated RNAP model (gray) and the legend (blue) is incorrect.

We thank the reviewer for catching this error. We have corrected the legend in the revised Fig. 5b — the post-translocated RNAP model is shown in gray, and the legend now correctly reflects this.

5. Fig. S4: The coloring and labeling conventions of the RNAP and the nucleotides in A should be consistent with those in B and C.

We thank the reviewer for this observation. We have revised the original Fig. S4 (updated as Fig. S9 in the revised version) to ensure consistent coloring and labeling conventions across all panels.

6. Table S1 and Fig S2: Only 25k particles were used for the map for TL-closed dP:Z*TP, which seems like very few for a resolution of 2.75 Å. However, the FSC curves look fine, so that resolution is believable.

We thank the reviewer for this comment. The 2.75 Å resolution achieved from ~25,000 particles is consistent with the high signal-to-noise achievable from a well-ordered complex with the Titan Krios/Falcon 4i detector system at our data collection conditions. The gold-standard FSC curves, which cross 0.143 at 2.75 Å for both half-maps independently, validate this resolution estimate. No additional action is required, but we have added a brief note in the Methods to acknowledge the particle count and explain the high resolution achieved.

7. Table S1: The model resolution should be reported at 0.5 FSC (Å), not 0.143 FSC like the map. Additionally, it is always positive to report the model-map FSC graphs in their entirety.

We thank the reviewer for this correction. We have revised Table S1 to report model-to-map FSC resolution at the 0.5 threshold and included the complete model-map FSC curves as a new Fig. S10.

Reviewer #3

I co-reviewed this manuscript with one of the reviewers who provided the listed reports. This is part of the Nature Communications initiative to facilitate training in peer review and to provide appropriate recognition for Early Career Researchers who co-review manuscripts.

We thank this early career reviewer and fully support *Nature Communications* initiative to facilitate training in peer review and to provide appropriate recognition for Early Career Researchers who co-review manuscripts.

Reviewer #4

This is an excellent and crisply written piece that continues to deepen the explorations from the Benner lab on artificial base-pairing, bio-orthogonal, biomimicking systems with potentially massively important applications in biotechnology.
The point here is to a) expand on fine-tuning of the Z-P base pair by changing the nitro to the carboxamide and b) to demonstrate how both of these are incorporated using cryo-EM. While I am not thoroughly qualified to evaluate the quality of the cryo-EM data, the figures are convincingly presented and provide fine-structure rationalization of how E.coli RNA polymerase can use these modified ribonucleotides.

The work nicely pairs structural work with in-vitro kinetic analysis of matched and mismatched incorporation studies.

We thank the reviewer for the positive feedback and recognition of the significance of our work.

A few minor revisions are required to enhance clarity.
1.The authors use what seems to be a primer extension reaction where they've hybridized an RNA primer to the template strand and then there's another DNA oligo listed as being part of the non-template strand. The authors need to properly reference this assay and explain how this represents an elongating complex. While it's been a while that I've looked at in vitro models of transcription, this doesn't address open-complexes or initiation complexes. Minimally a discussion as to where this assay was developed and to what extent it captures other aspects of transcription e.g. initiation would be appreciated. If the authors wanted to go further, they should run transcription on bona-fide strong procaryotic promoters like LacUV5 or Gal4. One might ask here (or maybe in future work) whether these Hachimoji bases can be inserted at start-sites of transcription and/or present in the promoter regions.

We thank the reviewer for raising this point. In this study, we focus on transcription elongation. To bypass the low efficiency and heterogeneity of initiation steps (promoter melting, abortive transcription, sigma factor dissociation, initiation-to-elongation transition) from a promoter-dependent transcription system, we adopted a synthetic scaffold method to mimic functional elongation complexes (developed in von Hippel[5,6] and Kashlev labs[7]). These reconstituted EC methods are widely used in many labs in the field (including us) for studying bacterial RNAP elongation biochemistry and structural biology. It reproduces the structural state of RNAP during processive transcript elongation, as validated by high-resolution cryo-EM structures of *E. coli* RNAP assembled on equivalent synthetic scaffolds, including our previous structural study of the B:S base pair system using the same approach[1]. The recognition and incorporation of UBP substrates occur exclusively during the elongation phase, governed by the geometry of the RNAP active site at the i+1 position, which is fully and faithfully recapitulated in the synthetic scaffold EC. We have added a clarifying description of the system and its validation to the Methods section of the revised manuscript.

The reviewer's suggestion to test UBP incorporation on authentic prokaryotic promoters such as LacUV5 represents a valuable and important future direction that would demonstrate whether Hachimoji bases are tolerated during promoter-initiated transcription and multi-round elongation. We acknowledge this as a compelling extension of the current work and have added a comment to the Discussion accordingly. Gal4 is a eukaryotic activator and would require a eukaryotic RNA polymerase context; for bacterial systems, promoters such as LacUV5 or T7A1 would be appropriate candidates for follow-up experiments.

2. While these studies certainly demonstrate significant fidelity w/respect to *E.coli* RNA polymerase, multiple elongations and longer-transcripts with either sequence analysis or RT-PCR using DNA bases would be good to demonstrate multiple incorporations and scoring fidelity. Admittedly this might be the basis of a different paper.

We thank the reviewer for this constructive suggestion. The current study addresses a key aspect of continued elongation through the NTP-chase experiments presented in Fig. R4. In those experiments, *E. coli* RNAP was first loaded with the cognate UBP substrate at the i+1 position, after which the next natural NTP was added as a chase. Robust extension products were observed for all four UBP configurations tested (dZ:PTP, dP:ZTP, dZ*:PTP, and dP:Z*TP), demonstrating that RNAP translocates efficiently after UBP incorporation and remains catalytically competent for continued synthesis. These data establish that UBP incorporation is not a termination event and that the elongation complex can re-enter the transcription cycle following P:Z or P:Z* pairing.

The multi-UBP transcript scenario suggested by the reviewer, in which a template contains multiple UBP positions and the full-length RNA is verified by RT-PCR and sequencing, represents an important and compelling next step. This approach would provide a direct, sequence-level readout of transcriptional fidelity across multiple UBP sites in a single transcript. Conceptual support for this possibility is provided by our previous structural study of the B:S base pair system[1], which demonstrated that *E. coli* RNAP recognizes both the B:S and P:Z pairs through the same unified Watson-Crick geometry, suggesting that these AEGIS pairs are mechanistically compatible with processive elongation. We fully agree with the reviewer that demonstrating multi-UBP incorporation with sequence-level verification is an important future goal, and we have acknowledged it as such in the revised Discussion.

3. In terms of showing the effects of the nitro-modified base, if the nitro results in undesired deprotonation, why was it used in the first place. This is unclear; I had to draw resonance structures to appreciate how a carbocyclic nucleoside that would otherwise possess the same functionalization as C would normally adopt a DAD tautomer except for the fact that the nitro is there. Reminding the reader as to how the nitro functions to alter the resonance forms by showing the non-aromatic nitronate form would help the reader appreciate why the nitro was chosen in the first place and why the amide serves a similar purpose.

We thank the reviewer for this question, which touches on an important aspect of Z's chemistry that we agree deserves clearer explanation in the manuscript.

The development of Z variant has a long history. The three Watson-Crick hydrogen bonds of the Z:P base pair are formed by C6-NH_2 (donor), N1-H (donor), and C2=O (acceptor). Initially, this was implemented without any substituents at C6. This proved to be too sensitive to oxidation to be used in DNA synthesis, even in protected form. The pyrimidine (with an N replacing C5) had tautomeric ambiguity; we use it as a biversal base. Therefore, we made a pyrazine with the hydrogen bond donor-donor-acceptor pattern (with N replacing C4). This proved to epimerize too easily. Hence, the 5-nitro pyridine was added, to reduce the sensitivity of the aminopyridone to oxidation (Yang et al. Nucleic Acids Res 2006)[8]. This had a 15 year history, but expedients to minimize the Z:G mispairing required that we change the nitro group. This history is reviewed in Kim et al. (2025)[4]. In any case, the nitro and carboxyamido groups at C5 are oriented toward the major groove and do not participate directly in any of these three hydrogen bonds. We have also made the Z variant with a cyano group. Further, this is not a carbocyclic nucleoside. It is a C-glycoside. And yes, if it were an N-glycoside, it would not have the correct hydrogen bonding pattern.

The consequence of using the nitro group is, as the reviewer correctly identifies, a reduction in the pKa of the adjacent ring NH to approximately 7.8. At physiological pH, a fraction of Z therefore exists in the deprotonated form (Z^-), in which N1-H loses its proton and $N1^-$ converts from a hydrogen bond donor to an acceptor. This change creates a hydrogen bonding pattern complementary to guanine, generating the Z:G mismatch illustrated in Fig. 2A. In Z*, the nitro group is replaced by a carboxamide, which is a weaker electron-withdrawing substituent and raises the pKa above 10, effectively suppressing Z^- formation and

eliminating the Z:G mismatch. We have revised the manuscript text to make this chemical logic explicit, especially as shown in Kim et al. (2025)[4].

4.When a mismatch in favor of Z-G is found, this is interesting since I would have thought that A would be favored given the possibility of forming the DAD tautomer although Figure 2 nicely shows how the deprotonated Z pairs with G. To the authors' credit they show this misincorporation however this may overestimate its misincorporation since in the absence of P, the enzyme is forced to introduce a G.

We thank the reviewer for these observations. Regarding the expectation that A rather than G would be the mismatch partner: the Z:G mismatch arises specifically through the deprotonated form Z^{-}, which presents three hydrogen bond contacts complementary to guanine (as shown in Fig. 2A). The Watson-Crick face of adenine presents only two hydrogen bond contacts (N1 acceptor and N6-H donor), which would not support a three-hydrogen-bond interaction with Z^{-}. The Z:G mismatch is therefore geometrically and chemically favored over Z:A, consistent with the biochemical data in Fig. 1B and Fig. R1.

Regarding the potential overestimation of misincorporation frequency, the reviewer raises a valid point that we have addressed in detail in our response to Reviewer 1, Question 2 (Reviewer 1-Question 2). Briefly, the single-nucleotide transcription assay contains only one NTP species per reaction, with no competing substrates present. Under these conditions, RNAP incorporates whichever NTP is available, which systematically overestimates the misincorporation frequency relative to conditions where all NTPs compete simultaneously. In vivo, the high-affinity cognate substrate PTP would kinetically outcompete GTP, such that the Z:G mismatch rates observed here represent an upper bound rather than a physiologically accurate estimate. We have revised the manuscript to acknowledge this limitation explicitly.

Other minor issues:
Figure 1: show the H-bonds, don't write NH2

We thank the reviewer for this suggestion. We have revised Fig. 1 to display hydrogen bonds as dashed lines between donor and acceptor atoms, replacing the text label "NH2" with explicit hydrogen bond representations.

Reference:

1. Oh, J. *et al.* A unified Watson-Crick geometry drives transcription of six-letter expanded DNA alphabets by E. coli RNA polymerase. *Nat Commun* **14**, 8219 (2023).
2. Dhingra, Y., Landick, R., Campbell, E. A. & Darst, S. A. RNA polymerase inhibitors reveal active-site motions essential for the nucleotide-addition cycle. 2026.04.06.716786 Preprint at https://doi.org/10.64898/2026.04.06.716786 (2026).
3. Zhu, B. *et al.* Synthesis of 2′-Fluoro RNA by Syn5 RNA polymerase. *Nucleic Acids Res* **43**, e94 (2015).

4. Kim, H.-J., Wenta, A. J., Dobrzycki, L. M., Biondi, E. & Benner, S. A. Improving the Fidelity of Replication of a Six-Letter DNA Alphabet. *ACS Chem. Biol.* https://doi.org/10.1021/acschembio.5c00724 (2025) doi:10.1021/acschembio.5c00724.
5. Daube, S. S. & von Hippel, P. H. RNA displacement pathways during transcription from synthetic RNA-DNA bubble duplexes. *Biochemistry* **33**, 340–347 (1994).
6. Daube, S. S. & von Hippel, P. H. Functional transcription elongation complexes from synthetic RNA-DNA bubble duplexes. *Science* **258**, 1320–1324 (1992).
7. Sidorenkov, I., Komissarova, N. & Kashlev, M. Crucial role of the RNA:DNA hybrid in the processivity of transcription. *Molecular Cell* **2**, 55–64 (1998).
8. Yang, Z., Hutter, D., Sheng, P., Sismour, A. M. & Benner, S. A. Artificially expanded genetic information system: a new base pair with an alternative hydrogen bonding pattern. *Nucleic Acids Research* **34**, 6095–6101 (2006).

Point-by-point Response:
Reviewer #2

1. Line 181-182: Repeat “for dG template” (Similarly, for dG template, we also observed multiple bands of ZTP misincorporation for the dG template)
We thank the reviewer for catching this repetition and have corrected it accordingly.
2. Line 191: Did you mean “substituent”
We thank the reviewer for catching this typographical error; the intended word was "substituent" and we have corrected it.
3. Fig. S7 is mentioned after Fig. S8 and S9
We thank the reviewer for catching this inconsistency and have corrected the figure order accordingly.
4. Fig. S8 why do they compare dZ:PTP to dG:CTP rather than dC:GTP (unless there is not a structure of that)
We thank the reviewer for this observation, and agree that dC:GTP would be the more direct comparator; however, no published dC:GTP structure is available, so we used the available dG:CTP EC as the native Watson-Crick reference instead.
5. Line 453: Remove line
We thank the reviewer for this comment and have removed the line accordingly.
6. A statement clearly indicating that the Z:P pair needs further optimization. I think the paper succeeds at showing the viability of pKa engineering like that done here, so indicating that this is the first step of further improvement directly would be good.
We thank the reviewer for this suggestion. We have revised the text to state clearly that further refinement of the Z scaffold will be required to achieve a fully optimized P:Z pair, and to frame the pKa engineering strategy demonstrated in this work (Z*) as an initial step toward that goal.
